# Rare, Serious, and Comprehensively Described Suspected Adverse Drug Reactions Reported by Surveyed Healthcare Professionals in Uganda

**DOI:** 10.1371/journal.pone.0123974

**Published:** 2015-04-23

**Authors:** Ronald Kiguba, Charles Karamagi, Paul Waako, Helen B. Ndagije, Sheila M. Bird

**Affiliations:** 1 Department of Pharmacology and Therapeutics, Makerere University College of Health Sciences, Kampala, Uganda; 2 Clinical Epidemiology Unit, Makerere University College of Health Sciences, Kampala, Uganda; 3 National Pharmacovigilance Centre, National Drug Authority, Kampala, Uganda; 4 Medical Research Council Biostatistics Unit, Cambridge, United Kingdom; Carl von Ossietzky University of Oldenburg, GERMANY

## Abstract

**Background:**

Lack of adequate detail compromises analysis of reported suspected adverse drug reactions (ADRs). We investigated how comprehensively Ugandan healthcare professionals (HCPs) described their most recent previous-month suspected ADR, and determined the characteristics of HCPs who provided comprehensive ADR descriptions. We also identified rare, serious, and unanticipated suspected ADR descriptions with medication safety-alerting potential.

**Methods:**

During 2012/13, this survey was conducted in purposively selected Ugandan health facilities (public/private) including the national referral and six regional referral hospitals representative of all regions. District hospitals, health centres II to IV, and private health facilities in the catchment areas of the regional referral hospitals were conveniently selected. Healthcare professionals involved in prescribing, transcribing, dispensing, and administration of medications were approached and invited to self-complete a questionnaire on ADR reporting. Two-thirds of issued questionnaires (1,345/2,000) were returned.

**Results:**

Ninety per cent (241/268) of HCPs who suspected ADRs in the previous month provided information on five higher-level descriptors as follows: body site (206), drug class (203), route of administration (127), patient age (133), and ADR severity (128). Comprehensiveness (explicit provision of at least four higher-level descriptors) was achieved by at least two-fifths (46%, 124/268) of HCPs. Received descriptions were more likely to be comprehensive from HCPs in private health facilities, regions other than central, and those not involved in teaching medical students. Overall, 106 serious and 51 rare previous-month suspected ADRs were described. The commonest serious and rare ADR was Stevens-Johnson syndrome (SJS); mostly associated with oral nevirapine or cotrimoxazole, but haemoptysis after diclofenac analgesia and paralysis after quinine injection were also described.

**Conclusion:**

Surveyed Ugandan HCPs who had suspected at least one ADR in the previous month competently provided comprehensive ADR descriptions: more, indeed, than are received per annum nationally. Properly analyzed, and with local feed-back, voluntary ADR reports by HCPs could be an essential alerting tool for identifying rare and serious suspected ADRs in Uganda.

## Background

Adverse drug reactions (ADRs) contribute to the global burden of ill-health[1–3]. Efficient ADR detection and reporting systems in sub-Saharan Africa (SSA) could promote more effective management of medicines and foster improvements in the quality of healthcare[4]. The detection or suspicion of ADRs by healthcare professionals (HCPs) is a first step in ADR reporting[5]. The ability of HCPs to suspect or recognize ADRs and ably to differentiate them from patients’ illnesses and/or to attribute them across co-administered medications are, however, major challenges[6]. Ugandan HCPs involved in medical research and non-nurses were more likely to have suspected an ADR in the previous month, but previous-month receipt of a patient ADR complaint was the dominant factor in triggering ADR suspicion[5].

The spontaneous suspected ADR reporting system in Uganda currently requires completion, by HCPs, of a paper-based ADR report form, see S1 Appendix for details. Only a small proportion (13%) of previous-month suspected ADRs that surveyed Ugandan HCPs detected in routine clinical practice were reported[5], consistent with ADR under-reporting elsewhere[7]. National statistics for voluntary ADR reporting to the Ugandan National Pharmacovigilance Centre (NPC) indicate that only 0.44% of HCPs report suspected ADRs annually[5].

Lack of adequate detail in the majority (80%) of suspected ADR reports submitted to the NPC[8] is an additional challenge. ‘Completeness’, as defined by Uppsala Monitoring Centre (UMC)[9], is a country-level quantitative measure of how complete is the information on 12 key details, see S2 Appendix, in Individual Case Safety Reports (ICSRs) that UMC receives from NPCs. We defined a HCP-level comprehensive ADR description as having at least four of the following five higher-level descriptors: body site, drug class, route of administration, patient’s age, and ADR severity. Since we were testing the ability of HCPs to provide focal “free text” ADR descriptions, our target was to identify descriptions with, at most, one missing higher-level descriptor. Except for patient age, none of the other four basic descriptors is explicitly an ICSR key detail. Our higher-level descriptors could be the starting point for the completeness evaluation process by which NPCs elicit UMC’s 12 key details, which include the information ideally needed to assess the causality of suspected ADRs: temporal relationship to drug intake, response to drug withdrawal, event or laboratory test abnormality, presence/absence of disease process or other drugs that can explain the ADR, and re-challenge if possible[10]. The large proportion of reports with inadequate detail, as submitted to the NPC, poses a challenge for signal detection and causality assessment of suspected ADRs: underestimation of the true risks of patients’ medications is the result[11]. This study is focused on basic ADR description; we do not address ADR causality.

Ugandan HCPs are encouraged to submit comprehensive reports on suspected ADRs internally within the healthcare system and, ultimately, these reports ought to reach the NPC. Such submissions promote informed risk-benefit evaluation of medications and improve overall patient safety. We investigated how comprehensively Ugandan HCPs described their most recent previous-month suspected ADR, and determined the characteristics of HCPs who provided comprehensive ADR descriptions. We also identified rare, serious, and unanticipated suspected ADR descriptions with medication safety alerting potential.

## Methods

### Study design and sampling procedure

From 25 May 2012 to 28 February 2013, we conducted a cross-sectional study in Uganda in purposively selected, geographically diverse public and private health facilities. Details of the survey-design, anonymous self-completion of survey questionnaire, and sampling procedures have been described elsewhere[5]. Briefly, health facilities were selected to include the national referral hospital, and six regional referral hospitals which were selected to be representative of the Central, Eastern, Northern, Southern, and Western regions of Uganda. For practical purposes, district hospitals, health centres II to IV, and private health facilities (both for-profit and not-for-profit) in the catchment areas of the regional referral hospitals were conveniently selected. Healthcare professionals involved in prescribing, transcribing, dispensing medication orders, and administration of medicines to patients in the selected health facilities were eligible. Pre-trained research assistants approached HCPs and invited them to complete a pretested self-administered questionnaire. Healthcare professionals who accepted to participate gave separately-held written informed consent[5]. The self-completed questionnaires were tracked using serial numbers and they did not elicit identifying information on individual HCPs[5]. Two-thirds of the issued questionnaires were returned.

### Data collection and management

The self-administered questionnaire was used to obtain demographic and professional information; and description of the most recent previous-month suspected ADR[5], see S3 Appendix. Descriptions of the most recent suspected ADR were rated on the presence/absence of five higher-level descriptors: body site, drug class, route of administration, patient’s age, and ADR severity. Comprehensiveness of ADR description was defined by the presence of at least four descriptors.

We distinguished between a severe suspected ADR and a serious suspected ADR. A serious ADR is one that can result in death, is life-threatening, requires initial or prolonged hospitalization, or results in disability or incapacitation[12]. Severity of an ADR describes its intensity as: mild, moderate or severe[13]. Thus, a severe ADR (severity grade 3) is not synonymous with a serious ADR[13].

We defined rare ADRs as those which are reported to occur less often than in10 per 10,000 individuals on specified medication (≤ 0.1%)[14]; and unanticipated ADRs as those which, to our knowledge, are not supported by existing literature.

When invited to describe their most recent previous-month suspected ADR, some HCPs provided descriptions for two to three such cases. We used simple random sampling to retain one ADR description from each such set for analysis, see S4 Appendix.

All data were entered into a databank using EpiData 3.1.

### Analysis

Free-text descriptions for all ADRs and how we coded them for the five higher-level descriptors are provided in the S5 Appendix. Severity of ADRs, as reported by HCPs, was not altered by the study investigators in subsequent analyses.

Two investigators (RK & SMB) each independently assessed the comprehensiveness of the 241 ADR descriptions: disagreements were resolved by consensus, and the consensus judgement on the five higher-level descriptors is provided in the S5 Appendix.

Seriousness of ADRs was assessed by RK based on the definition by the WHO Uppsala Monitoring Centre (UMC) classification[12] while the assessment of rareness and unanticipated ADR was conducted by RK using the online British National Formulary as the key reference guide[15].

Questionnaire responses and higher-level ADR descriptors were summarized as frequencies and percentages. We used logistic regression to assess demographic and professional determinants of comprehensiveness in describing the most recent of past-month suspected ADRs. Regression coefficients were expressed as odds ratios (ORs) with 95% confidence intervals and were obtained using Stata 12.0[16]. We accounted for missing data using the missing-assigned approach where low-frequency missing data were meaningfully assigned to an existing category.

Descriptions of serious (incapacitating or life-threatening) suspected ADRs were tabulated by drug class, see S6 Appendix; and for similar listing of severe (grade 3 intensity) suspected ADRs, see S7 Appendix. For ease of assimilation, rare suspected ADRs were grouped by whether or not SJS was explicitly mentioned; if not, rare ADRs were listed by the named drug.

### Ethical clearance

The School of Medicine Research and Ethics Committee, Makerere University College of Health Sciences, and the Uganda National Council for Science and Technology provided ethical approval for the study.

## Results

### Study population

Overall return-rate for the survey was 67% (1,345/2,000) and the mean number of patients seen per day by HCPs was 41 [(SD = 46), median of 30 (interquartile range from 15 to 50 patients/day; n = 1,226 of 1,345)]. Only 21% (268/1,289) of HCPs reported that they had suspected an ADR in the previous month. The mean age of respondent HCPs who suspected an ADR in the previous 4-weeks was 32.2 years (SD = 8.5; n = 222 of 268). Twenty seven (10%) of the 268 HCPs who suspected an ADR in the previous month did not provide any ADR description. Of the 241 who did, 95 (39%) were nurses, 77 (32%) were doctors, 23 (10%) were pharmacists/pharmacy technicians and 46 (19%) were other cadres.

### Suspected ADRs

Of the 268 HCPs who had suspected an ADR in the previous month, 241 (90%) provided a free-text description of the most recently encountered previous-month suspected ADR with higher-level details given as follows: *body-site* [85%, 206/241; skin only reactions were the modal group (43%, 88/206)]; *drug class* [84%, 203/241; with the top three single classes cited as follows: antibacterials (70), antiretrovirals (45), and antimalarials (28)]; *route of administration* [(explicitly cited: 53%, 127/241), although, in addition, route could be inferred from the named-drug in a further 63 descriptions (hence, 79%); with 50 of 190 being by injection]; *patient’s age* [55%, 133/241; mean age 28.3 years (SD = 16.5), median of 28 years and inter-quartile range from 20 to 36 years]; and *ADR-severity* [53%, 128/241; with 46% (59/128) of citations being in respect of severe reactions], see Table 1 and S4 Appendix.

**Table 1:** Survey-description of the most recent past-month suspected ADR provided by 241 out of 268 HCPs who suspected ADRs in the previous month, Uganda, 2013.

**Body site distribution for 206 suspected ADR descriptions**			
**Body site of ADR**	**Frequency**	**Per cent**	**Per cent-body site reported**
Skin only	88	37%	43%
Central Nervous System (CNS) only	20	8%	10%
Gastrointestinal Tract (GI) only	18	7%	9%
Skin & CNS	6	3%	2%
Skin & GI	4	2%	2%
CNS & GI	4	2%	2%
Other	66	27%	32%
Not reported	35	14%	-
**Total**	**241**	**100%**	**100% **
**Drug class distribution for 203 suspected ADR descriptions**			
**Drug class**	**Frequency**	**Per cent**	**Per cent-drug class reported**
Antibacterials only	70	29%	34%
Antiretrovirals only	45	19%	22%
Antimalarials only	28	12%	14%
Antibacterials & Antiretrovirals	1	0%	1%
Antibacterials & Antimalarials	4	1%	2%
Other	55	23%	27%
Not reported	38	16%	-
**Total**	**241**	**100%**	**100%**
**Route of drug administration for 127 suspected ADR descriptions**			
Route of administration*	**Frequency**	**Per cent**	**Per cent-route reported**
Oral or Topical	83	34%	65%
Injectable	44	18%	35%
Not reported	114	47%	-
**Total**	**241**	**100%**	**100%**
**Patient’s age distribution for 133 suspected ADR descriptions**			
**Patient age (n = 133)**	**Mean (SD)**		**Median (IQR)**
Measure of central tendency	28.3 (16.5)		28 (20–36)
**ADR Severity distribution for 128 suspected ADR descriptions**			
**Severity of ADR**	**Frequency**	**Per cent**	**Per cent—severity**
Mild	27	11%	21%
Moderate	42	17%	33%
Severe	59	24%	46%
Not reported	113	47%	-
**Total**	**241**	**100%**	**100%**

*Route of administration could be inferred for a further 63 (6 were by injection & 59 were oral) ADR descriptions.

### Comprehensive survey-described suspected ADRs

Comprehensiveness of ADR description was achieved by 46% (124/268; 95% CI: 40% to 52%) of HCPs when only 85 would have been expected by independence of descriptors, see Table 2.

**Table 2:** Comprehensiveness of ADR-description of the most recent past-month suspected ADR(s) as reported by 241 out of 268 HCPs who suspected ADRs in the previous month, Uganda, 2013.

HIGHER-LEVEL DETAILS in addition to ADR	Body Site	Drug Class	Route	Age of patient	Severity	Observed number	Expected by independence
**All 5 provided**	**√**	**√**	**√**	**√**	**√**	**54**	**17**
	206/268	203/268	127/268	133/268	128/268		
**Combinations of 4**							
Combination 1	**√**	**√**	**√**	**√**		22	19
Combination 2		**√**	**√**	**√**	**√**	**10**	**5**
Combination 3	**√**	**√**		**√**	**√**	19	20
Combination 4	**√**		**√**	**√**	**√**	2	6
Combination 5	**√**	**√**	**√**		**√**	17	18
**TOTAL**						**124**	**85**

The more comprehensive ADR descriptions were received from HCPs in private health facilities [vs. public; private not-for-profit (OR = 3.4, 95% CI: 1.48 to 7.82) & private for-profit (OR = 2.6, 95% CI: 1.13–5.96)], and regions other than Central Uganda [Eastern (OR = 2.6, 95% CI: 1.20–5.73) & combined Western-Northern-Southern (OR = 6.4, 95% CI: 2.85–14.37)]. Comprehensive ADR description was less likely from HCPs who were involved in teaching medical students (OR = 0.5, 95% CI: 0.23–1.00) and was not significantly influenced by professional cadre (nurse versus non-nurse), life-time encountering of a fatal ADR, age of HCP, receipt of a previous-month patient ADR complaint, involvement in medical research, patient load, department, and knowing to whom to report ADRs, see Table 3.

**Table 3:** Personal and professional factors associated with comprehensive ADR description of most recent past-month suspected ADR for 241 healthcare professionals who suspected ADR(s) in the previous month and provided a description, Uganda, 2013.

Factor	Comprehensive ADR Description		Crude Analysis			Adjusted Analysis		
	Yes (%)	No (%)	OR	95%CI	P-value	OR	95%CI	P-value
Level of Health Facility								
Other	37 (56)	29 (44)	1.0			1.0		
Hospital	87 (50)	88 (50)	0.8	0.44–1.37	0.380	2.0	0.86–4.47	0.110
Type of Health Facility								
Public	49 (41)	71 (59)	1.0			1.0		
Private Not-For-Profit	31 (67)	15 (33)	3.0	1.46–6.13	0.003	3.4	1.48–7.82	0.004
Private For-Profit	44 (59)	31 (41)	2.1	1.14–3.70	0.016	2.6	1.13–5.96	0.024
Region of the country								
Central	49 (37)	82 (63)	1.0			1.0		
Eastern	34 (62)	21 (38)	2.7	1.42–5.18	0.003	2.6	1.20–5.73	0.015
Other	41 (75)	14 (25)	4.9	2.43–9.89	<0.001	6.4	2.85–14.37	<0.001
Professional Cadre								
Nurse	54 (57)	41 (43)	1.0			1.0		
Non-nurse	70 (48)	76 (52)	0.7	0.42–1.18	0.178	1.1	0.54–2.32	0.769
Age								
Less than 30 years	53 (50)	54 (50)	1.0			1.0		
Aged 30 years or older	71 (53)	63 (47)	1.1	0.69–1.91	0.594	1.7	0.92–3.09	0.090
Patient Load								
Greater than 30/day	59 (50)	58 (50)	1.0			1.0		
At most 30/day	65 (52)	59 (48)	1.1	0.65–1.80	0.757	1.4	0.78–2.66	0.241
Department								
Medicine	70 (52)	64 (48)	1.0			1.0		
Surgery	4 (31)	9 (69)	0.4	0.12–1.38	0.150	0.4	0.09–1.47	0.156
Paediatrics, OBS & Gyn	16 (43)	21 (57)	0.7	0.33–1.45	0.334	0.7	0.29–1.64	0.404
Other	34 (60)	23 (40)	1.4	0.72–2.53	0.347	1.0	0.48–2.04	0.973
Teaching medical students								
No	101 (57)	75 (43)	1.0			1.0		
Yes	23 (35)	42 (65)	0.4	0.23–0.73	0.003	0.5	0.23–1.00	0.050
Ever encountered fatal ADR								
No	98 (55)	79 (45)	1.0			1.0		
Yes	26 (41)	38 (59)	0.6	0.31–0.99	0.044	0.7	0.35–1.42	0.327
Involved in medical research								
No	75 (51)	71 (49)	1.0			1.0		
Yes	49 (52)	46 (48)	1.0	0.60–1.69	0.975	1.8	0.96–3.43	0.068
Gender								
Male	57 (47)	64 (53)	1.0			1.0		
Female	67 (56)	53 (44)	1.4	0.85–2.36	0.176	1.4	0.70–2.82	0.334
Suggestions for improved ADR reporting*								
No	21 (49)	22 (51)	1.0					
Yes	103 (52)	95 (48)	1.1	0.59–2.20	0.705			
Knows to whom to report*								
No	51 (45)	63 (55)	1.0					
Yes	73 (58)	54 (42)	1.7	1.00–2.78	0.049			
Patient ADR complaint*								
No	35 (55)	29 (45)	1.0					
Yes	89 (50)	88 (50)	0.8	0.47–1.49	0.546			

* Covariates not fitted into the final logistic regression model.

### Serious suspected ADRs

Serious suspected ADRs constituted 44% (106/241) of the ADR descriptions; see S6 Appendix for list of the 106 serious suspected ADRs. The most frequent body sites of serious ADRs were skin only (33%, 34/102), central nervous system (CNS) only (11%, 11/102), and gastrointestinal tract (GI) only (8%, 8/102), see Table 4. The most frequent *single* drug classes of serious ADRs were antiretrovirals (35%, 31/88), antibacterials (15%, 13/88) and antimalarials (14%, 12/88), see Table 4. Serious suspected ADRs accounted for only 24% (13/53: 95% CI: 13%- 36%) of antibacterial-only-linked ADR descriptions but for 82% (31/38: 95% CI: 70%- 94%) of antiretroviral-only-linked suspected ADR descriptions, and for 46% (12/26: 95% CI: 27%- 65%) of antimalarial-only-linked suspected ADRs [Chi (2 df) = 29.35; P < 0.001]. Severity (grade or intensity) was not reported for half (53/106) of the serious (incapacitating or life-threatening) ADRs.

**Table 4:** Seriousness of the most recent past-month suspected ADRs described by 241 out of 268 HCPs who suspected ADRs in the previous month, Uganda, 2013.

**Serious ADR distribution for 241 suspected ADR descriptions (n, %)**				
**Serious ADR**	**Body Site Specified**		**Drug Class Specified**	**Frequency**
Yes	102 (50)		88 (43)	106 (44)
No	93 (45)		84 (41)	97 (40)
Unassessable	11 (5)		31 (15)	38 (16)
**Total**	**206 (100)**		** 203 (100)**	**241 (100)**
**Serious ADR by Body Site for 206 suspected ADR descriptions**				
**Body Site**		**Serious ADR (n, %)**			
	**Yes**	**No**	**Unassessable**	**Total**
Skin only*	34 (33)	47 (51)	7 (64)	88 (43)
CNS only*	11 (11)	9 (10)	0 (0)	20 (10)
GI only*	8 (8)	9 (10)	1 (9)	18 (9)
Skin & CNS	3 (3)	3 (3)	0 (0)	6 (3)
Skin & GI	1 (1)	3 (3)	0 (0)	4 (2)
CNS & GI	2 (2)	2 (2)	0 (0)	4 (2)
Other	43 (42)	20 (21)	3 (27)	66 (31)
**Total**	**102 (100)**	**93 (100)**	**11 (100)**	**206 (100)**
**Serious ADR by Drug Class for 203 suspected ADR descriptions**				
**Drug Class**	**Serious ADR (n, %)**			
	**Yes**	**No**	**Unassessable**	**Total**
Antibacterials only*	13 (15)	40 (48)	17 (55)	70 (34)
Antiretrovirals only*	31 (35)	7 (8)	7 (23)	45 (22)
Antimalarials only*	12 (14)	14 (17)	2 (6)	28 (14)
Antibacterial & Antiretroviral	0 (0)	0 (0)	1 (3)	1 (1)
Antibacterials & Antimalarials	4 (4)	0 (0)	0 (0)	4 (2)
Other	28 (32)	23 (27)	4 (13)	55 (27)
**Totals**	**88 (100)**	**84 (100)**	**31 (100)**	**203 (100)**
**Rare ADR**	**Serious ADR (n, %)**			
	**Yes**	**No**	**Unassessable**	**Total**
Yes	33 (31)	18 (19)	0 (0)	51 (21)
No	46 (43)	57 (59)	6 (16)	109 (45)
Unknown	27 (25)	22 (22)	32 (84)	81 (34)
**Total**	**106 (100)**	**97 (100)**	**38 (100)**	**241 (100)**

Chi-square (2 df) = 6.42, P < 0.025 (if the “unassessable” column is excluded)

*Proportions of serious ADRs **(across rows)**, and their 95% confidence intervals (95% CI) for a) body site: skin only, **39% (95% CI: 29%- 49%);** CNS only, **55% (95% CI: 33%- 77%);** GI only, **44% (95% CI: 21%- 67%); &** other, **42% (95% CI: 30%- 54%);** and for, b) drug class: antibacterials only, **24% (95% CI: 13%- 36%)**; antimalarials only, **46% (95% CI: 27%- 65%);** antiretrovirals only, **82% (95% CI: 70%- 94%);** & other, **58% (95% CI: 45%- 71%)**

### Case reports of serious suspected ADRs

A 28-year-old patient received cotrimoxazole tablets and developed black patches on the skin (reported by a 35-year-old female registered midwife in a public health centre IV, Tororo, Eastern Uganda); severe haemoptysis (coughing up of blood) in a patient after two days on oral diclofenac (reported by a 28-year-old male doctor in a private for-profit hospital in Kampala, Central Uganda); and, a 17-year-old receiving intramuscular quinine developed severe post injection paralysis (reported by a 60-year-old female enrolled nurse in a private-not-for-profit hospital in Mbarara district, Southern Uganda), see S8 Appendix.

### Rare suspected ADRs

Fifty one rare suspected ADRs (which occur in ≤ 0.1% of medication users) were reported, of which 65% (33/51) were serious, see Tables 4 and 5. Among the rare ADR descriptions, Stevens-Johnson syndrome (SJS) was explicitly mentioned in 13 of 51 cases and implicated drugs were: antiretroviral therapy [five of 13 cases were nevirapine-linked, and one was linked to zidovudine-lamivudine post-exposure prophylaxis]; cotrimoxazole (septrin) [four]; ciprofloxacin [one]; carbamazepine [one]; and no drug mentioned [one].

**Table 5:** Survey-descriptions of 51 rare suspected Adverse Drug Reactions (ADRs) of Healthcare Professionals (HCPs) who suspected ADRs in the past 4 weeks.

Level/Facility	Type/Facility	district	Region	Cadre	Nurse-cadre	HCP-Gender	HCP-Age	Rare ADR Description	Source
**SJS Explicitly Mentioned**									
Public	Private Hospital	KAMPALA	Central	Doctor		Male	26	24YR/FEMALE KNOWN IMMUNOSUPPRESSED SYNDROME (ISS) PATIENT ON ANTI-TBS WHO REACTED TO COTRIMOXAZOLE—SJS & ALSO HAD TOXOPLASMOSIS	Cotrimoxazole; < 1 in 10,000—NHS, BNF
Private For-Profit	Private Hospital	TORORO	Eastern	Doctor		Female	28	A MAN DIAGNOSED WITH HIV TOOK COTRIMOXAZOLE AND GOT STEVENS-JOHNSON SYNDROME SEVERE	Cotrimoxazole; < 1 in 10,000—NHS, BNF
Private Not-for-Profit	District Hospital	MASINDI	Western	Nurse	Registered Nurse Midwife	Female	43	40YRS,SEPTRIN,ORALLY.SJS	Cotrimoxazole; < 1 in 10,000—NHS, BNF
Private Not-for-Profit	Private Hospital	TORORO	Eastern	Other		Female	25	38YR/MALE ON SEPTRIN DEVELOPED SJS—MODERATE	Cotrimoxazole; < 1 in 10,000—NHS, BNF
Private For-Profit	National Referral	KAMPALA	Central	Doctor		Female	32	36YR MALE ADMITTED WITH SJS FOLLOWING INITIATION OF ORAL NEVIRAPINE,SEVERE EVENT*	NVP; 0.1–0.3%—ehealthme (1 per 1000),Mittmann et al 2012 (1–2 per 1000)
Private For-Profit	Health Centre III	MASAKA	Southern	Other		Female	27	32YR OLD REACTED TO ORAL NEVIRAPINE—SJS. REACTION WAS MODERATE	NVP; 0.1–0.3%—ehealthme (1 per 1000), Mittmann et al 2012 (1–2 per 1000)
Private For-Profit	Private Hospital	WAKISO	Central	Doctor		Male	27	HIV+/FEMALE NEWLY ENROLLED ON HAART WITH NVP-BASED REGIMEN DEVELOPED SJS WHICH WAS SEVERE	NVP; 0.1–0.3%—ehealthme (1 per 1000), Mittmann et al 2012 (1–2 per 1000)
Private Not-for-Profit	District Hospital	KAMPALA	Central	Doctor		Female	33	28YR FEMALE ON ORAL DUOVIR-N (AZT/3TC/NVP), GOT RASH WHICH WORSENED & SHE DEVELOPED SJS	BNF
Public	District Hospital	TORORO	Eastern	Nurse	Registered Nurse	Female	48	32YR/FEMALE ISS PATIENT ON AZT/3TC/NVP FOR 3 MONTHS GOT SKIN RASH ALL OVER THE BODY—STEVEN JOHNSON SYNDROME—SEVERE	BNF
Public	Regional Referral	GULU	Northern	Pharm		Female	26	22YR/FEMALE LADY NURSE WHO GOT NEEDLE STICK INJURY & WAS INITIATED ON PEP WITH AZT/3TC-12 DAYS LATER GOT SEVERE RASH (SJS) & WAS ADMITED AND TREATED	Drugs.com: Database powered by Wolters Kluwer Health, American Society of Health- System Pharmacists, Cerner Multum and Thomson Reuters Micromedex. Not in BNF.
Public	Health Centre III	JINJA	Eastern	Pharm		Female	26	25YR OLD FEMALE ON CIPROFLOXACIN IV. GOT SJS.IT WAS SEVERE	BNF
Private For-Profit	National Referral	KAMPALA	Central	Doctor		Female	25	SJS SECONDARY TO CARBAMAZEPINE	BNF
Private Not-for-Profit	National Referral	KAMPALA	Central	Nurse	Registered Nurse	Female	27	SJS, MUCOSITIS (WHERE PATIENT CANNOT PUT ANYTHING IN THE MOUTH), DROP IN BLOOD COUNTS	No drug stated but SJS known to be a rare ADR
**Co-trimoxazole (Septrin)—Other Reactions**									
Private Not-for-Profit	Health Centre IV	BUIKWE	Central	Nurse	Enrolled Comprehensive	Male	25	58YR OLD ON ORAL COTRIMOXAZOLE GOT SEVERE URTICARIA AND SKIN RASHES ALL OVER THE BODY	BNF
Public	Health Centre III	GULU	Northern	Other		Male	26	32YR/FEMALE KNOWN ISS PATIENT ON COTRIMOXAZOLE CAME WITH SEVERE BODY RASHES & SLOUGHING*	BNF
Private For-Profit	National Referral	KAMPALA	Central	Other		Male	32	62YR/FEMALE ON COTRIMOXAZOLE ORAL ROUTE WITHIN TWO DAYS GOT MULTIPLE SKIN PATCHES, DEVELOPED SORES ON MUCOUS MEMBRANES WITH HIGH TEMPERATURE. GIVEN STEROIDS & SHE RECOVERED	BNF
Public	Health Centre III	MITOOMA	Western	Other		Male	27	HIV+ PATIENT WITH BODY ACHES, SUDDEN SKIN RASH, BURNT SKIN, OOZING OF SWEAT-LIKE FLUID AFTER COTRIMOXAZOLE ADMINISTRATION—MILD SYMPTOMS	BNF
Private For-Profit	Health Centre IV	TORORO	Eastern	Nurse	Registered Midwife	Female	35	28YR OLD PATIENT WAS ADMINISTERED WITH COTRIMOXAZOLE TABLETS DEVELOPED BLACK PATCHES ON SKIN—MODERATE	BNF
Private For-Profit	Other	KAMPALA	Central	Nurse	Registered Nurse	Male	33	42YR/FEMALE SERO-POSITIVE, NAIVE TO HAART WITH MILD ITCHY RASH, APPETITE LOSS, RISE IN BODY TEMPERATURE & ITCHING WHICH INTENSIFIED ON SWALLOWING SEPTRIN TABLETS FIVE WEEKS AGO. PATIENT RETURNED TO FACILITY & LAB INVESTIGATIONS SHOWED DERRANGED LIVER FUNCTION TEST VARIABLES	BNF
Public	Drug Shop	KAMULI	Eastern	Nurse	Enrolled Nurse	Female	28	OLD WOMAN REACTED TO SEPTRIN,GENERALIZED BODY SORES,GAVE HER PREDISOLONE & PAIN KILLER PLUS BETADERM TOPICAL (BETAMETHASONE)	BNF
Public	Health Centre III	JINJA	Eastern	Nurse	Enrolled Nurse	Female	20	26YR/MALE HIV+ ON ORAL SEPTRIN,GOT BURNT FACE & LIPS-GIVEN ORALDEXAMETHASONE FOR FIVE DAYS—SEVERE	BNF
Public	District Hospital	TORORO	Eastern	Nurse	Nursing Assistant	Female	50	A 20YR OLD TAKING SEPTRIN ORALLY GOT SEVERE SKIN RASH ALL OVER THE BODY	BNF
**Quinine only**									
Public	Health Centre III	KAMPALA	Central	Other		Male	32	28YR/FEMALE GOT BODY ITCHING AFTER TAKING ORAL QUININE. ADR WAS MODERATE	Itching = Pruritus; Drugs.com
Private For-Profit	Drug Shop	KAMULI	Eastern	Nurse	Nursing Assistant	Male	40	55YR OLD MALE ON QUININE TABS ORAL ROUTE GOT BODY ITCHING	Itching = Pruritus; Drugs.com
Private For-Profit	Pharmacy	JINJA	Eastern	Pharm		Female	35	PATIENT REACTED TO QUININE WITH ITCHING, TREATED IT WITH CETIRIZINE	Itching = Pruritus; Drugs.com
Public	Drug Shop	JINJA	Eastern	Nurse	Registered Midwife	Male	30	28YR OLD FEMALE REACTED TO QUININE IV LEADING TO MISCARRIAGE	Manufacturer's information leaflet: Drugs.com
Public	Private Hospital	MBRA	Southern	Nurse	Enrolled Nurse	Female	50	PATIENT ON ORAL QNN GOT SKIN RASH,TINNITUS,ABORTION,VERTIGO, NAUSEA, VOMITING, BLURRED VISION—SEVERE	Manufacturer's information leaflet-miscarriage = abortion: Drugs.com
**Quinine & Septrin**									
Private For-Profit	Private Hospital	TORORO	Eastern	Nurse	Enrolled Comprehensive	Female	27	8YR OLD GIRL REFERRED FROM A CLINIC AFTER RECEIVING IV QUININE & SEPTRIN DEVELOPED BLISTERS ALL-OVER THE BODY & DIED ON ADMISSION—SEVERE	BNF
Private Not-for-Profit	Private Hospital	TORORO	Eastern	Nurse	Enrolled Comprehensive	Female	30	5YR OLD GIRL FROM A CLINIC WHERE SHE WAS PUT ON IV QUININE & SEPTRIN. GOT BLISTERS ALL OVER THE BODY & DIED ON ADMISSION—SEVERE	BNF
Private For-Profit	Private Hospital	TORORO	Eastern	Nurse	Nursing Assistant	Female	29	5YR OLD GIRL WAS REFERRED FROM A CLINIC AFTER TAKING IV QUININE & SEPTRIN WITH BLISTERS ALL-OVER THE BODY. SHE DIED ON ADMISSION—SEVERE	BNF
**Coartem**									
Private Not-for-Profit	Health Centre IV	KAMPALA	Central	Other		Male	34	SWELLING OF FACE IN 16YR PATIENT AFTER SWALLOWING COARTEM (ORALLY)-MODERATE	Drugs.com
Private For-Profit	Regional Referral	LIRA	Northern	Nurse	Registered Nurse	Female	27	26YR/MALE ON COARTEM ORAL ROUTE,RASH ON BOTH HANDS, ITCHING & SWELLING	Drugs.com
Public	Health Centre IV	JINJA	Eastern	Doctor		Male	24	52YR OLD FEMALE ON COARTEM ORAL ROUTE DEVELOPED SORES ON THE WHOLE BODY	Drugs.com
**Mefloquine**									
Private Not-for-Profit	Health Centre IV	MASINDI	Western	Doctor		Male	62	62YR/FEMALE ON ORAL MEPHAQUINE GOT SEVERE HEADACHE WITH MENTAL CONFUSION & INSOMNIA	Drugs.com
**Fansidar**									
Private Not-for-Profit	Health Centre IV	TORORO	Eastern	Other		Male	24	36YR OLD ON ORAL FANSIDAR (SULPHADOXINE/PYRIMETHAMINE) DEVELOPED HYPERPIGMENTATION OF THE SKIN & THE THROAT—MILD	Drugs.com
**Diclofenac**									
Private Not-for-Profit	Private Hospital	KAMPALA	Central	Doctor		Male	28	ORAL DICLOFENAC 50MG, HAEMOPTYSIS AFTER 2 DAYS—WAS SEVERE*	Yiannakopoulou EC 2011, Van Renterghemet al 2012
Private For-Profit	Private Hospital	TORORO	Eastern	Nurse	Registered Comprehensive	Female	29	65YR/FEMALE PATIENT'S BLOOD PRESSURE LOWERED FROM 105/60 TO 86/50 MMHG & STARTED SWEATING AT A TEMPERATURE OF 35.0 DEGREES CELSIUS DUE TO DYNAPAR (IV DICLOFENAC INFUSION)—MILD	Drugs.com
**NVP-based HAART Regimen**									
Public	National Referral	KAMPALA	Central	Doctor		Female	39	45YR PATIENT ON ORAL TDF/3TC/NVP GOT SEVERE ABDOMINAL PAIN WHICH WAS IN COLICKY FORM	Drugs.com: Abdominal pain rare for Tenofovir
Public	National Referral	KAMPALA	Central	Doctor		Male	29	ISS PATIENT WITH SKIN RASH,SCALING,ULCERATIONS FOR 2WKS THAT STARTED 1WK AFTER ART INITIATION OF COMBIVIR-NEVIRAPINE (AZT/3TC/NVP)	BNF
**Efavirenz**									
Public	District Hospital	BUIKWE	Central	Nurse	Registered Nurse	Female	25	MILD ADR.PATIENT ON ORAL EFV HAD GYNAECOMASTIA AND JERKS	BNF
**Ceftriaxone**									
Public	Private Hospital	KAMPALA	Central	Pharm		Female	24	REACTION TO CEFTRIAXONE WITH MILD INFLAMATION &SWELLING AT POINT OF INJECTION*	BNF
**Gentamicin**									
Public	Regional Referral	KAMPALA	Central	Doctor		Male	28	26YR ANAEMIC FEMALE WITH SEVERE PRE-ECLAMPSIA FINALLY DELIVERED & WAS MANAGED POST-OPERATIVELY WITH GENTAMICIN-SUSTAINED ACUTE RENAL FAILURE WITH ANAEMIA FOR 5 DAYS & OTHER COMPLICATIONS	BNF
**Ciprofloxacin**									
Private For-Profit	District Hospital	MASINDI	Western	Other		Male		28YR/MALE—ALLERGIC REACTION TO ORAL CIPROFLOXACIN—SEVERE URTICARIA—SUBSTITUTED FOR CEPHALEXIN & CETIRIZINE*	BNF
Public	Health Centre III	WAKISO	Central	Doctor		Female	30	CHILD ON IV CIPROFLOXACIN GOT BODY ITCH, SWELLING AROUND THE FACE & AROUND SITE OF THE INJECTION	BNF
Public	National Referral	KAMPALA	Central	Nurse	Registered Nurse	Female	26	PATIENT WAS ON CIPRO AND GOT SWOLLEN HANDS	BNF
**Metronidazole**									
Private For-Profit	Other	MASAKA	Southern	Nurse	Enrolled Nurse	Female	28	23YR OLD,FLAGYL,ITCHING & NAUSEA,MILD	BNF
**Omeprazole**									
Public	National Referral	KAMPALA	Central	Nurse	Registered Nurse Midwife	Female	31	SEVERE PALPITATIONS DUE TO OVER DOSE OF OMEPRAZOLE	Drugs.com
**Paracetamol**									
Private Not-for-Profit	National Referral	GULU	Northern	Doctor		Female	35	41YR/MALE GIVEN PANADOL (PARACETAMOL), STARTED SHAKING & SWEATING 10 MINUTES LATER.WAS PUT ON A DRIP AND HE BECAME FINE.	Drugs.com: Database powered by Wolters Kluwer Health, American Society of Health- System Pharmacists, Cerner Multum and Thomson Reuters Micromedex. Not in BNF.
Public	Regional Referral	LIRA	Northern	Doctor		Female	40	10YR/MALE REACTED TO PANADOL (PARACETAMOL) WHEN TOOTH WAS REMOVED, BECAME DIZZY-MINOR	Mescape—frequency not defined
**Phenobarbitone**									
Public	District Hospital	TORORO	Eastern	Nurse	Registered Mental Health	Female	32	18YR/MALE MAN TAKING ORAL PHENORBABITONE GOT RASHES ALL OVER THE BODY SEVERE	BNF
**Piperazine**									
Public	Pharmacy	MBRA	Southern	Other		Male		24YR/FEMALE DEVELOPED AN INFLAMATORY ITCHY RASH ALL-OVER AFTER TAKING ORAL PIPERAZINE,STOPPD IT AND PATIENT IMPROVED—MODERATE	BNF

**KEY:** ADR = ADVERSE DRUG REACTION; ART = ANTIRETROVIRAL THERAPY; AZT = ZIDOVUDINE; BNF = BRITISH NATIONAL FORMULARY; EFV = EFAVIRENZ; FLAGYL = METRONIDAZOLE; HAART = HIGHLY ACTIVE ANTIRETROVIRAL THERAPY; ISS = IMMUNOSUPRESSED SYNDROME; IV = INTRAVENOUS; MEPHAQUINE = MEFLOQUINE; NVP = NEVIRAPINE; SEPTRIN = COTRIMOXAZOLE; SJS = STEVENS-JOHNSON SYNDROME; TDF = TENOFOVIR; 3TC = LAMIVUDINE;

* = CHECK APPENDIX S4 FOR TWO OR MORE DESCRIBED ADVERSE DRUG REACTIONS; PHARM = PHARMACIST/PHARMACY TECHNICIAN.

Additional ADR descriptions involving skin ulcerations, blistering, sloughing/peeling, and black skin patches; burnt face and lips; sore mucous membranes or general body sores; and death were linked to cotrimoxazole [12 of 38 cases] and nevirapine [1 of 38 cases] administration. Severe urticaria, swelling of the face, hands, and injection site were associated with ciprofloxacin use [3 of 38 cases]. Death, miscarriage, skin blistering and body itching were associated with quinine use [8 of 38 cases]. Severe palpitations due to overdose of omeprazole [1 case]; haemoptysis (coughing up of blood) after oral administration of diclofenac [1 case]; and mental confusion due to mefloquine [1 case] were also reported; for 11 others cases, see Table 5.

### Unanticipated suspected ADRs

Six unanticipated suspected ADRs (not backed by existing literature) were described: generalized body sores after oral coartem (artemether-lumefantrine); hypotension and sweating after intravenous diclofenac; reduced blood pressure and low pulse after chloramphenicol injection; severe burning sensation in the private parts immediately after intravenous hydrocortisone; irritability after intravenous quinine; and shaking and sweating for 10 minutes after receiving paracetamol, see S4 Appendix.

### Summary of suspected ADRs

In summary, 106 serious, 51 rare, and six unanticipated past-month ADRs were described by 1,345 respondent HCPs, who, on average, saw 41 patients daily (SE = 1.3). Including overlaps between the patients seen, the reporting period accounts for over 1.5 million patient-days (1,345*41*28 = 1,544,060 patient-days). If our respondents can be considered representative, and for future planning, we estimate that, per 4-weeks, 100 Ugandan HCPs may encounter eight (8) serious, four (4) rare, and fewer than one (0.5) unanticipated suspected ADRs.

## Discussion

Ninety percent (241/268) of surveyed HCPs who suspected an ADR in the previous month described the most recent past-month suspected ADR they had encountered. Although the proportion of HCPs who provided these ADR description(s) was high, elsewhere we have reckoned[5] that only a small proportion (13%) of previous-month suspected ADRs that our surveyed Ugandan HCPs detected in routine clinical practice was formally reported. Moreover, the number of ADR reports (241 reports from 1,345 HCPs) in our study was higher than the Ugandan National Pharmacovigilance Centre’s annual mean number of ADR reports (207 reports from 46,566 HCPs) over the previous six and a half years[5]. Our study, therefore, demonstrated a high level of motivation to describe the most recent previous-month suspected ADRs amongst the surveyed HCPs; however, the impetus for voluntary reporting of suspected ADRs in routine clinical practice is still very low[5].

Half of the ADR descriptions provided (124/241) were comprehensive. The extent of comprehensive ADR description is laudable given the fact that we elicited information on the most recent previous-month suspected ADR by recall and without prior sensitization of the HCPs about this survey. Half of the reports explicitly cited route of administration but route could nonetheless be inferred from the named drug in a further 26% (63/241) of descriptions. To eliminate errors in analysis of reported suspected ADRs, HCPs ought always to report drug route clearly.

Completeness and relevance are VigiBase database measures coined by the Uppsala Monitoring Centre (UMC) to assess the amount and quality of detail in Individual Case Safety Reports (ICSRs) received from National Pharmacovigilance Centres[9]. Completeness is quantitative and it measures to what level an ICSR is complete after NPC has liaised with the HCP-reporter, see S2 Appendix, while relevance is qualitative and can be used to assess causal relationships between a drug and an ADR[9]. The relevance algorithm, however, is still being developed[9]. Our findings could enhance the process of developing algorithms used to assess the level of detail and quality of information in ADR reports, such as need for explicit route of administration. It is the responsibility of individual countries to improve the quality of ADR reports that they receive from HCPs.

Comprehensive “free text” suspected ADR description provides additional detailed qualitative information that could enhance “completeness” and “relevance” assessment of ICSRs by NPCs, and generates stronger medication safety signals which, in turn, might promote proper characterization of reported suspected ADRs. More certainty about the nature of suspected ADRs and efficiency in taking appropriate measures to prevent these medication safety threats to patients is the result. Determining the characteristics of HCPs who are less likely to provide comprehensive ADR reports can help to identify HCPs to whom interventions aimed at improving the comprehensiveness (and ultimately overall quality) of ADR reporting can be targeted while at the same time utilizing the skills of the more comprehensive ADR reporters to strengthen/support improvements in the overall quality of suspected ADR reporting in Uganda.

Published literature which gives an in-depth account of the level of detail in ADR descriptions reported by HCPs in sub-Saharan Africa (SSA) was lacking. This study, therefore, provided empirical data on HCP-described ADRs in diverse health facilities in a Ugandan setting. The aim was to stimulate further research and intellectual discourse on comprehensiveness of ADR reporting in SSA in order to inspire similar, periodic but prospectively designed studies, to understand and improve the overall quality of ADR-reporting by HCPs in limited-resource settings where the use of technology-aided electronic ADR signal detection in daily clinical practice is not yet widely implemented. We assessed five higher-level descriptors provided in free-text recalled descriptions, but acknowledge that suspected ADR reports ought also to have relevant information to facilitate ADR causality assessment[10] and risk-benefit evaluation of medicines used in daily clinical practice[17]. For this reason, we recommend that a prospective version of our survey-approach to recording suspect ADRs be put in place by hospitals: for example, in two randomly selected months every year. Prospective recording during the survey months not only allows HCPs’ multiple reports of the same suspected ADR to be identified but also allows contemporary consideration of causality and of the ward’s medication-frequency for implicated drugs.

Working at private health facilities was associated with more comprehensive ADR descriptions. Thus, whereas past-year ADR reporting was lower in private-for-profit health facilities as compared with the public health sector[5], survey ADR descriptions from HCPs in private settings were three times more likely to be comprehensive. Any future interventions aimed at raising the ADR reporting rate by HCPs in both private and public health facilities should take into account both stimulation of, and the quality of, submitted ADR reports.

Healthcare professionals in regions other than in central Uganda had a higher likelihood to report comprehensive ADR descriptions. This finding might reflect the intensive NPC-led pharmacovigilance training focused on HCPs in regional referral hospitals when regional pharmacovigilance centres were first established in Uganda.

Healthcare professionals who were involved in the teaching of medical students were less likely to provide comprehensive ADR descriptions and yet it is these HCPs who bear the responsibility of introducing, to the medical students, the basic principles of pharmacovigilance during the formative years of the students’ medical training. Targeted interventions to improve the comprehensiveness of ADR reports received from HCPs in teaching hospitals as well as lower level health facilities are essential to the strengthening of grassroots pharmacovigilance in Uganda. To stimulate more comprehensive ADR reporting, tables of serious and rare ADRs, such as those reported in this paper, should be provided both locally and nationally as feedback to HCPs. These tables group the received ADR reports meaningfully and indicate the rarity of the described ADR, where available, according to conventional references.

Lifetime encountering of a fatal ADR was not related to comprehensive ADR reporting. Thus, whereas occurrence of a fatal ADR might stimulate ADR reporting[5], it may not be an important factor in determining the comprehensiveness of reporting on suspected ADRs. Professional cadre, age of HCP, receipt of a previous-month patient ADR complaint, involvement in medical research, patient load, department, and knowing to whom to report ADRs were not statistically significantly associated with the reporting of comprehensive ADR descriptions among the surveyed HCPs.

The comparative advantage of post-marketing pharmacovigilance over randomized control trials (RCTs) is the ability to detect rare, serious, unanticipated, and/or delayed suspected ADRs that occur after a new drug product has been licensed for use on the open market. Moreover, RCTs are conducted in fewer subjects and in an experimental environment that may not adequately reflect the conditions of the “real word” healthcare setting[18, 19]. Spontaneous and voluntary ADR reporting systems provide an inexpensive post-marketing pharmacovigilance system that can be used to identify and prevent ADRs (e.g. rare and serious ones) and other drug-related problems.

Severity (grade or intensity) was not reported for half of the serious (incapacitating or life-threatening) ADRs suggesting that Ugandan HCPs rate seriousness higher than severity or that they do not take reporting of severity to be as important.

Miscarriage, post-injection paralysis, and child deaths were examples of serious ADRs associated with quinine use. Post-injection paralysis has previously been found to be associated with the bad clinical practice of inappropriate injection of quinine into the sciatic nerve by inadequately trained HCPs[20], which necessitated Uganda’s policy change of the quinine injection site from the gluteus muscle to the thigh[21]. Adult doses of oral and intravenous quinine can be administered safely in pregnancy[15] but high doses may cause miscarriage[22].

Rare ADRs occur in [1–10] per 10,000 individuals (0.01% to 0.1%) and very rare ADRs occur in fewer than 1 in 10,000 (< 0.01%) individuals receiving a specified medicine[14]. Two-thirds (65%, 95% CI: 33/51) of rare suspected ADRs were serious thus underlining the importance of pharmacovigilance in tracking medication safety since reported rare ADRs are usually serious (incapacitating or life-threatening)[17]. Stevens-Johnson syndrome (SJS), a rare but serious skin-associated ADR, was the most frequently reported, and it was mostly linked to the use of the antimicrobials (antibacterials and antiretrovirals), specifically nevirapine and cotrimoxazole. Similarly, antimicrobials were the most likely cause of SJS in a multicentre retrospective study conducted in India where nevirapine [9 of 32 cases] and cotrimoxazole [7 of 32 cases] also contributed most to the frequency of observed SJS[23]. The one case of diclofenac-associated haemoptysis described in this survey corroborates the findings of two recently documented case reports: one in Switzerland[24] and another in Belgium[25]. Rare and potentially fatal suspected ADRs are more likely to be identified after drugs have been licensed for use on the open market and voluntary ADR reporting by HCPs is a cornerstone of generating alerting adverse reaction signals[17].

One limitation to this study is that some suspected ADR(s) were described by more than one HCP(s)[5]. Due to differences in the level of detail provided in the various ADR descriptions, we were unable to establish the full extent of multiply described incidents, a challenge that may be encountered by any voluntary ADR reporting system but one that can be mitigated if ADRs are described comprehensively. A second limitation is recall bias owing to the use of self-report but we mitigated this challenge by restricting the recall period for the ADR description to the previous one month. A third limitation is that neither the reporters nor the lead author performed ADR causality assessment to confirm causal relationships between the suspected ADRs and the implicated drugs. Although the rare, serious or unanticipated ADRs reported in this paper should be interpreted cautiously, the study investigators cross-checked each suspected ADR against authoritative reference texts using the online British National Formulary as the main reference[15]. We also maintain that HCPs need only to suspect and subsequently to report suspected adverse reactions to pharmacovigilance (PV) units without the prior requirement for formal causality assessment. Thus, NPCs should have the skilled personnel to be able to perform ADR causality assessment. Moreover, it is in this voluntary system of ADR reporting that a number of ADRs relating to post-marketed drugs are first identified.

Our study has generated key positive insights into how comprehensive and how alerting are the suspected ADRs reported by surveyed Ugandan HCPs.

## Conclusions

Surveyed Ugandan HCPs who had suspected at least one ADR in the previous month competently provided comprehensive ADR descriptions: more, indeed, than are received per annum nationally. Properly analyzed, and with local feed-back, voluntary ADR reports by HCPs could be an essential alerting tool for identifying rare and serious suspected ADRs in Uganda.

## Supporting Information

S1 Appendix Ugandan suspected ADR form and reporting scheme. (PDF)

S2 Appendix Completeness quantification of ICSRs (PDF)

S3 Appendix Study Questionnaire (PDF)

S4 Appendix List of 241 ADR Descriptions (PDF)

S5 Appendix Comprehensiveness of 241 ADR Descriptions (PDF)

S6 Appendix List of 106 serious ADR Descriptions (PDF)

S7 Appendix List of 59 severe ADR Descriptions (PDF)

S8 Appendix Case reports of suspected ADRs (PDF)

## References

[1] Patel KJ, Kedia MS, Bajpai D, Mehta SS, Kshirsagar NA, Gogtay NJ. Evaluation of the prevalence and economic burden of adverse drug reactions presenting to the medical emergency department of a tertiary referral centre: a prospective study. BMC Clinical Pharmacology. 2007;7:8 . Pubmed Central PMCID: PMC1963321. Epub 2007/07/31. eng. 17662147 10.1186/1472-6904-7-8 PMC1963321

[2] Davies EC, Green CF, Taylor S, Williamson PR, Mottram DR, Pirmohamed M. Adverse drug reactions in hospital in-patients: a prospective analysis of 3695 patient-episodes. PLoS One. 2009;4(2):e4439 Epub 2009/02/12. eng. 10.1371/journal.pone.0004439 19209224 PMC2635959

[3] Tumwikirize WA, Ogwal-Okeng JW, Vernby A, Anokbonggo WW, Gustafsson LL, Lundborg SC. Adverse drug reactions in patients admitted on internal medicine wards in a district and regional hospital in Uganda. Afr Health Sci. 2011 3;11(1):72–8. . Epub 2011/05/17. eng. 21572860 PMC3092317

[4] WHO Draft Guidelines for Adverse Event Reporting and Learning Systems, (2005).

[5] Kiguba R, Karamagi C, Waako P, Ndagije HB, Bird SM. Recognition and reporting of suspected adverse drug reactions by surveyed healthcare professionals in Uganda: key determinants. BMJ Open. 2014;4(11):e005869 10.1136/bmjopen-2014-005869 25421337 PMC4244492

[6] Pradel Editions. Adverse drug reactions: a practical guide to diagnosis and management Baffins Lane, Chichester, West Sussex PO19 1UD, England: John Wiley and Sons Ltd; 1994 [cited 2014 19 September]. Available: http://eu.wiley.com/WileyCDA/WileyTitle/productCd-0471942111.html#.

[7] Hazell L, Shakir SA. Under-reporting of adverse drug reactions: a systematic review. Drug Safety. 2006;29(5):385–96. . Epub 2006/05/13. eng. 16689555 10.2165/00002018-200629050-00003

[8] National Pharmacovigilance Centre. Working together with the Public Health Programmes: a regulator’s perspective for addressing the minimum requirements for Pharmacovigilance Kampala, Uganda: National Drug Authority; 2010 [cited 2013 21 June]. Available: http://www.who.int/medicines/areas/quality_safety/regulation_legislation/icdra/WG-2_2Dec.pdf.

[9] World Health Organization (WHO)—Uppsala Monitoring Centre (UMC). Documentation grading—how complete are the reports?: The Uppsala Monitoring Centre; 2011 [cited 2014 1 October]. Available: http://www.monitoringmedicines.org/graphics/26755.pdf.

[10] World Health Organization (WHO) Uppsala Monitoring Centre (UMC). Causality Assessment of Suspected Adverse Reactions [20 September 2011]. Available: http://who-umc.org/Graphics/24734.pdf.

[11] Gavaza P, Brown CM, Lawson KA, Rascati KL, Wilson JP, Steinhardt M. Influence of attitudes on pharmacists' intention to report serious adverse drug events to the Food and Drug Administration. Br J Clin Pharmacol. 2011 7;72(1):143–52. Epub 2011/02/22. eng. 10.1111/j.1365-2125.2011.03944.x 21332572 PMC3141196

[12] World Health Organization Collaborating Centre for International Drug Monitoring (UMC). Safety Monitoring of Medicinal Products: Guidelines for Setting Up and Running a Pharmacovigilance Centre 2000 [cited 2015 15 January]. Available: http://apps.who.int/medicinedocs/en/p/printable.html.

[13] Division of AIDS. Table for Grading the Severity of Adult and Pediatric Adverse Events 2009 [cited 2014 31 July]. Available: http://rsc.tech-res.com/Document/safetyandpharmacovigilance/Table_for_Grading_Severity_of_Adult_Pediatric_Adverse_Events.pdf.

[14] World Health Organization (WHO) Uppsala Monitoring Centre (UMC). Definitions [cited 2014 4 August]. Available: http://www.who.int/medicines/areas/quality_safety/safety_efficacy/trainingcourses/definitions.pdf.

[15] British National Formulary. London: BMJ Group and Pharmaceutical Press; 2013.

[16] StataCorp. Stata Statistical Software: Release 12. College Station, TX: StataCorp LP; 2011.

[17] Council for International Organizations of Medical Sciences (CIOMS). Benefit-Risk Balance for Marketed Drugs: Evaluating Safety Signals. Geneva1998 [cited 2014 4 August]. Available: http://www.cioms.ch/publications/g4-benefit-risk.pdf.

[18] Etminan M, Gill S, Fitzgerald M, Samii A. Challenges and opportunities for pharmacoepidemiology in drug-therapy decision making. J Clin Pharmacol. 2006 1;46(1):6–9. . 16397278 10.1177/0091270005283285

[19] Talisuna AO, Staedke SG, D'Alessandro U. Pharmacovigilance of antimalarial treatment in Africa: is it possible?. Malar J. 2006;5:50 . Epub 2006/06/20. eng. 16780575 10.1186/1475-2875-5-50 PMC1523354

[20] Ekure J. Gluteal Fibrosis. A report of 28 cases from Kumi Hospital, Uganda. East and Central African Journal of Surgery. 2006 4 2006;12(1):144–7.

[21] National Pharmacovigilance Centre. What is current practice of Pharmacovigilance in Uganda? Kampala, Uganda: National Drug Authority; 2012 [cited 2013 11 December]. Available: http://africapv2012.files.wordpress.com/2012/04/day-2_5_country-presentations-nras_h-ngadije-uganda-compatibility-mode.pdf.

[22] Actavis UK Ltd. Summary of Product Characteristics—Quinine Sulphate Tablets BP 300mg Whiddon Valley, Barnstaple, Devon, EX32 8NS, UK: Datapharm; 2013 [updated 22 April; cited 2014 19 September]. Available: http://www.medicines.org.uk/emc/medicine/24467.

[23] Barvaliya M, Sanmukhani J, Patel T, Paliwal N, Shah H, Tripathi C. Drug-induced Stevens-Johnson syndrome (SJS), toxic epidermal necrolysis (TEN), and SJS-TEN overlap: a multicentric retrospective study. Journal of Postgraduate Medicine. 2011 Apr-Jun;57(2):115–9. Epub 2011/06/10. eng. 10.4103/0022-3859.81865 21654132

[24] Yiannakopoulou EC. Hemoptysis under diclofenac and antiplatelet doses of aspirin. Pharmacology. 2011;87(1–2):1–4. . Epub 2010/12/15. eng. 21150234 10.1159/000321728

[25] Van Renterghem D, Depuydt C. Hemoptysis and Pulmonary Edema in a Scuba Diver Using Diclofenac. Pharmacology. 2012;89(1–2):103–4. 22354373 10.1159/000336059

