# Supplementary material for: Rare, Serious, and Comprehensively Described Suspected Adverse Drug Reactions Reported by Surveyed Healthcare Professionals in Uganda
Source: PLoS One. 2015 Apr 23;10(4):e0123974. doi: 10.1371/journal.pone.0123974 (PMC4408100; doi:10.1371/journal.pone.0123974)
Supplement: S1 Appendix — (PDF) [file pone.0123974.s001.pdf]

Appendix S1: Ugandan suspected ADR form and reporting scheme

REPUBLIC OF UGANDA
MINISTRY OF HEALTH

UGANDA NATIONAL DRUG AUTHORITY

CONFIDENTIAL

(See Reverse for instructions)

REPORT ON SUSPECTED ADVERSE DRUG REACTIONS

A. Patient's details

Surname

Other Names

Age | Sex | Weight (Kgs)

OPD No. | Health Facility name

District

B. Drug Details

(Including vaccines and other health care products)

Suspected Drug Brand Name ➡ | Generic Name ➡

Indication: Why was the drug prescribed or taken | Manufacturing / Expiry date ➡

Route ➡

Date Started ➡ | Date Stopped ➡ | Daily Dose ➡ | Diluent details ➡

Prescribed? YES ☐ NO ☐ | **OTC?** YES ☐ NO ☐ | Lot / Batch No. ➡

C. Reaction Details

- Headache ☐
- Shock / Collapse ☐
- Skin reaction ☐
- Diarrhea ☐
- Nausea or vomiting ☐
- Convulsions ☐
- Anaphylaxis ☐
- Severe local reaction ☐
- Injection site abscess ☐
- Other(specify)

Date Reaction Started ➡

Date of notification of Reaction ➡

Date Reaction Stopped ➡

Treatment given: Yes ☐ No ☐ If yes specify

Was patient admitted? YES ☐ NO ☐ Duration of admission (days) ➡

Outcome: Continuing ☐ Recovered ☐ Fatal ☐ Date of death

D. Other Drugs Used

(Including self medication, vaccines, herbal preparations) **Tick box** ☐ **if no drug taken**

	1	2	3	4	5
Name of drug					
Indication					
Daily dose					
Date started					
Date ended					

Comments: Please use this space to record other information e.g. relevant history, allergies, failure of efficacy, counterfeit, test result, follow up data etc.

F. Reporter Details

Name: | Designation:

Postal address: | Telephone / Fax:

Signature: | **Date:**

If you would like information about other adverse reactions associated with the suspected drug, please tick this box ☐

Thank you for your cooperation.

Guide on filling the Adverse Drug Reactions (ADR) reporting form

1. For the purpose of the pharmacovigilance programme, a drug or medicine is defined as, "Any substance administered to human beings for the prophylaxis, diagnosis or therapy of disease, or for the modification of physiological function". This definition includes prescribed medicines, over-the-counter medicines, vaccines, herbal medicines and biologicals including blood and blood-related products e.g. serum, plasma etc.
2. All health professionals in Uganda can report any suspected adverse drug reaction directly to NDA.
3. Please note that identities of the patient, reporting Doctor, Pharmacist, Nurse and health facility will be kept strictly confidential.
4. Please report any adverse reaction experienced by a patient even if you are not certain the product caused the adverse reaction or even if you do not have all the details.
5. This form can also be used to report product quality problems such as suspected contamination, questionable stability, defective components, poor packaging / labeling and/or therapeutic failures.
6. Please note that submission of a report does not imply that health professional or the product caused or contributed to the adverse reaction.
7. The completed suspected ADR report should be sent to the office of the District Director of Health Services (DDHS) to be forwarded to National Drug Authority at the address given below.
8. Date of Notification: Date a patient reports to the health facility.
9. The form must be forwarded to the next level (HSD/DDHS) within 24 - 48 hours of notification

HSD	Health Sub - District
DDHS	District Director of Health Services
UNEPI	Uganda National Expanded Program on Immunisation
ESD/MOH	Epidemiological Surveillance Division / Ministry of Health

The Executive Secretary / Registrar
National Drug Authority
National Pharmacovigilance Centre
Plot 46 - 48 Lumumba Avenue
P.O. Box 23096 Kampala, UGANDA
Tel: 255665 / 347391 / 2 / Hotline: 344052 Fax: 255758
Email: ndaug@nda.org.ug
Web site: www.nda.or.ug

Uganda's ADR reporting scheme.

The ADR form requests for details of the **patient** (name, age, sex, weight, health facility name whether patient was admitted – and, if so, duration of hospital stay, and district), **drug** (name, indication, manufacturing and expiry date, date drug was started and when stopped, dose, route of administration, diluents if any, whether prescribed or over-the-counter, other drugs used, and batch number), **suspected ADR** (nature of reaction, date reaction started and when it stopped, and outcome of ADR), and **reporter** (name, designation, postal address, signature and telephone). Healthcare professionals can report suspected ADRs either directly to the NPC or to Regional Pharmacovigilance Centres (RPCs) within their respective health facilities (RPCs). In peripheral health facilities, HCPs should deliver completed ADR reports, through a PV liaison, to one of the 14 RPCs housed at regional referral hospitals which, in turn, relay the ADR reports to the NPC. Scanned ADR reports can be emailed to the NPC but there is no official internet-based platform for online submission of suspected ADRs."
