# Supplementary material for: Rare, Serious, and Comprehensively Described Suspected Adverse Drug Reactions Reported by Surveyed Healthcare Professionals in Uganda
Source: PLoS One. 2015 Apr 23;10(4):e0123974. doi: 10.1371/journal.pone.0123974 (PMC4408100; doi:10.1371/journal.pone.0123974)
Supplement: S2 Appendix — (PDF) [file pone.0123974.s002.pdf]

<u>Appendix S2: Completeness Quantification of ICSRs</u>

National pharmacovigilance centres (NPCs) requested for feedback from Uppsala Monitoring Centre (UMC) on their submitted suspected ADR reports. As a part of this, the UMC Research Department developed the Documentation grading Completeness score, a measure of the amount of information on Individual Case Safety Reports (ICSRs).

Completeness is a VigiBase™ database measure coined by the Uppsala Monitoring Centre (UMC) to assess the amount of detail in ICSRs received from National Pharmacovigilance Centres[1]. Completeness is quantitative and it measures to what level an ICSR is complete [1]. The completeness score (0 to 1) utilizes information on 12 key details, namely; patient gender, report type [International Conference on Harmonization (ICH)-format or old WHO-format], primary source (cadre), time to ADR onset, patient age at onset, ADR date of onset, drug start date, outcome (recovery with/without sequelae, or death), indication [International Classification of Diseases-10 (ICD10) or Medical Dictionary for Regulatory Authorities (MedDRA) codes], free text, ADR date of onset consistency, and drug start date consistency[1].

Some general points to note regarding the Completeness score are:

- The score measures the information on ICSRs as they appear in VigiBase™, rather than as they appear in the Uganda National database. Thus, the score can identify issues in the process of extracting and sending ICSRs to UMC.
- The score can also identify changes that occur when a country changes its ICSR database or report format (for example, from INTDIS to E2B).
- The score is most meaningful when a country reports regularly (at least every quarter) to UMC.
- The score is difficult to interpret when only a small number of reports have been sent in a quarter.

We defined a <u>comprehensive ADR description</u> as having at least four of five higher-level descriptors: body site, medication class, route of administration, patient's age, and ADR severity. Except for patient age, none of the other four basic descriptors are explicitly mentioned in the 12 key ICSR details. These higher-level descriptors could form part of the evaluation process by which NPCs elicit UMC's 12 key details.

References

1. Documentation grading - how complete are the reports? Uppsala Reports 54. The Uppsala Monitoring Centre.
