# Supplementary material for: Rare, Serious, and Comprehensively Described Suspected Adverse Drug Reactions Reported by Surveyed Healthcare Professionals in Uganda
Source: PLoS One. 2015 Apr 23;10(4):e0123974. doi: 10.1371/journal.pone.0123974 (PMC4408100; doi:10.1371/journal.pone.0123974)
Supplement: S3 Appendix — (PDF) [file pone.0123974.s003.pdf]

Appendix S3: Pharmacoepidemiologic Assessment of Medication Errors and Adverse Drug Reactions among patients in Uganda

Investigator: ____________________ **District:** ____________________

An **Adverse Drug Reaction** (ADR) is any response to a drug which is harmful and unintended, and which occurs at doses normally used by patients.

HEALTH FACILITY CHARACTERISTICS

1. **Type of health facility** (*Tick one only*)<br>[1] Public<br>[2] Private Not-for-Profit<br>[3] Private For-Profit	2. **Level of health facility** (*Tick one only*)<br>[1] National Referral [5] Health Centre III<br>[2] Regional Referral [6] Health Centre II<br>[3] District Hospital [7] Private Hospital<br>[4] Health Centre IV [8] Other…………..

SOCIO-DEMOGRAPHIC CHARACTERISTICS OF PARTICIPANT

3. **Gender**<br>[1] Male<br>[2] Female	4. **How old are you (in complete years)?** ………..

PROFESSIONAL CHARACTERISTICS OF PARTICIPANT

5. **In which sector(s) do you practice**? (*Tick all that apply*)<br>[1] Public health facility<br>[2] Private Not-for-Profit health facility<br>[3] Private For-Profit health facility	6. **In which department are you?** (*Tick one only*)<br>[1] Medicine<br>[2] Surgery<br>[3] Paediatrics<br>[4] Obstetrics & Gynaecology<br>[5] Dentistry<br>[6] Pharmacy<br>[7] Other (Specify)……….
7. **What is the approximate number of patients you see per day**? ……………	8. **For how long have you been working in this health facility?** .... Months (If less than 1 year)<br>….. Completed Years
9. **What is your highest academic qualification?** (*Tick one only*)<br>[1] Certificate<br>[2] Diploma<br>[3] First Degree<br>[4] Masters Degree<br>[5] PhD	10. **For how long have you been practicing since you qualified with your highest academic training?**<br>…… Months (If less than 1 year)<br>…… Completed Years
11. **Do you teach medical students?**<br>[1] Yes [2] No (If no, **go to 13**)	12. **If yes, duration of practice in a teaching hospital**<br>…… Months (If less than 1 year)<br>…… Completed Years

**13. Are you actively involved in medical research?**<br><br>[1] Yes [2] No	**14. Professional Cadre** (*Tick one only*)<br><br>[1] Doctor (**go to 15**)<br>[2] Pharmacist (**go to 22**)<br>[3] Nurse (**go to 19**)<br>[4] Clinical officer (**go to 23**)<br>[5] Pharmacy Technician (**go to 22**)<br>[6] Other (Specify)………………………
**15. Position/Level of Doctor** (*Tick one only*)<br><br>[1] Senior Consultant<br>[2] Consultant<br>[3] Medical Officer Special Grade<br>[4] Medical Officer<br>[5] Senior House Officer<br>[6] Intern Doctor<br>[7] Other (specify)………….	**16. For how long have you been prescribing?**<br><br>…… Months (If less than 1 year)<br>..…. Completed Years
**17. What is the approximate number of prescriptions you write per day?**.............	**18. Have you given verbal prescriptions/orders to the attending nurse in the past 12 months?**<br><br>[1] Yes [2] No<br>(***Skip to 23***)
**19. Which of the following cadre category describes your qualification?** (*Tick one only*)<br><br>[1] Enrolled Midwife<br>[2] Enrolled Nurse<br>[3] Enrolled Mental Health Nurse<br>[4] Enrolled Comprehensive Nurse<br>[5] Registered Midwife<br>[6] Registered Nurse<br>[7] Registered Nurse/Midwife<br>[8] Registered Mental Health Nurse<br>[9] Registered Comprehensive Nurse<br>[10] Other (specify)……………………	**20. In some health facilities, nurses usually write out (transcribe) drug prescriptions from patients' medical records to medication charts. Are you required to transcribe prescriptions in your health facility?**<br><br>[1] Yes [2] No
**21. In practice, do you regularly transcribe prescriptions?**<br><br>[1] Yes [2] No<br>(***Skip to 23***)	**22. If pharmacist or pharmacy technician, area of practice** (*Tick all that apply*)<br><br>[1] Hospital [3] Academia<br>[2] Industry [4] Community/Private
**SUSPECTED ADVERSE DRUG REACTION (ADR) REPORTING PROGRAM**	
**23. Have you received any complaint of adverse drug reactions (ADRs) from patients in the last 4 weeks?**<br><br>[1] Yes [2] No (If no, **go to 25**)	**24. If yes, how many complaints of ADRs have you received in the last 4 weeks?** …….

25. Have you suspected an ADR in the last 4 weeks?

[1] Yes [2] No (If no, **go to 28**)

26. If yes, how many ADRs have you suspected in the last 4 weeks? ....

27. Briefly describe the most recent suspected ADR you encountered providing information on patient age, drug involved & route of administration, outcome of ADR & its severity (mild, moderate, severe); e.t.c.

………
………
………
………
………………………………………………………………………………………………

28. Have you ever encountered a fatal ADR that might have led to a patient's death?

[1] Yes [2] No

29. Have you reported any suspected ADR in the last 12 months?

[1] Yes [2] No (If No, **go to 35**)

30. If yes, please indicate the period within which you reported the most recent suspected ADR

[1]	[2]	[3]	[4]	[5]
**4 weeks**	**5-8 weeks**	**9-12 weeks**	**4-6 mo**	**7-12 mo**

31. To which authorities did you report the most recent of these ADRs?
(*Tick all that apply*)

[1] National Drug Authority (NDA)

[2] AIDS Treatment Information Centre (ATIC)

[3] Drug Manufacturer

[4] Medical Superintendent

[5] District Director of Health Services (DDHS)

[6] Institutional Review Board (IRB)

[7] Other (specify)………………................

32. What motivated you to report the suspected ADR?

………………………………….
………………………………….
………………………………….

33. Did you get any feedback about the ADR report(s) you submitted?

[1] Yes [2] No

34. Have you reported an ADR to the National Drug Authority in the past 12 months?

[1] Yes [2] No

35. Have you wanted to report an ADR in the past 12 months but did not have the ADR report form?

[1] Yes [2] No

36. Have you had an ADR suspicion in the past 12 months but did not fill the ADR report form even when you had it?

[1] Yes [2] No

37. Did you ever fill the ADR report form but failed to send it for any reason?

[1] Yes [2] No (If no, **go to 39**)

38. If yes, what was the reason(s) that you did not send the form on the most recent occasion?

……………………………….....
……………………………….....
……………………………….....
……………………………….....

39. Which of the following health workers are qualified to report adverse drug reactions? (Tick all that apply)

[1] Medical doctors [4] Pharmacists
[2] Dentists [5] Clinical Officers
[3] Nurses

**40. Pharmacovigilance relates to a reporting system for adverse effects of medicines. Have you ever heard about Pharmacovigilance?**<br>[1] Yes [2] No (If no, **go to 42**)	**41. If yes, please state the source(s) of your information** (Tick all that apply)<br>[1] Books/Journals<br>[2] Internet/e-communication<br>[3] Trainings/Seminars/courses attended<br>[4] Television<br>[5] Outdoor adverts<br>[6] Professional colleague<br>[7] Others (Specify)………………………….
**42. Are you aware of the existence of a National Pharmacovigilance Centre (NPC) in Uganda?**<br>[1] Yes [2] No (If no, **go to 44**)	**43. If yes, do you know where the NPC office is located?**<br>[1] Yes [2] No
**44. Have you ever seen the ADR form used for reporting ADRs to the NPC?**<br>[1] Yes [2] No (If no, **go to 48**)	**45. If yes, have you ever filled out the NPC ADR form?**<br>[1] Yes [2] No (If no, **go to 47**)
**46. Was the information on the NPC ADR form clear to you about what to report?**<br>[1] Yes [2] No	**47. Have you ever filled out any ADR form different from that of the NPC?**<br>[1] Yes [2] No
**48. Have you ever submitted an ADR report to the NPC?**<br>[1] Yes [2] No	**49. Do you know where to obtain the NPC ADR forms in this health facility?**<br>[1] Yes [2] No
**50. Do you know to whom to report ADRs in your health facility?**<br>[1] Yes [2] No (If no, **go to 52**)	**51. If yes, please specify in your health facility to whom you would report an ADR if you had to?**<br>…………………………………...<br>…………………………………...
**52. An ADR reporting system should;** (*Tick all that apply*)<br>[1] be compulsory<br>[2] be voluntary<br>[3] provide financial incentives to the reporter<br>[4] hide the identity of the prescriber<br>[5] hide the identity of the reporter<br>[6] hide the identity of the patient	**53. Have you ever been trained on how to report ADRs with the ADR form?**<br>[1] Yes [2] No
**54. Please suggest possible ways of improving ADR reporting**<br>……<br>……<br>……<br>……	

Instructions
In the left column are questions that will be the subject of your evaluation and in the right column is a gradual scale where you should mark with **X** the place along the scale where, according to your opinion, represents your degree of agreement with the text comment. The **extreme left** side indicates **total disagreement** while the **extreme right** indicates **total agreement**. Agreement increases as you move across from left to right
Please indicate whether you agree or disagree with the following statements
(1 = Strongly disagree; 2 = Slightly disagree; 3 = Neutral; 4 = Slightly agree; 5 = Strongly agree)

Strongly Disagree ⟷ Strongly Agree

	**Statement**	1	2	3	4	5
55	Serious ADRs are well documented by the time a drug is marketed					
56	It is nearly impossible to determine whether a drug is responsible for a particular adverse reaction					
57	I would only report an ADR if I was sure that it was related to the use of a particular drug					
58	The one case of an ADR that an individual health worker might see makes no significant contribution to medical knowledge					
59	I read articles about adverse drug reactions with interest					
60	I have a professional obligation to report ADRs					
61	Reporting ADRs puts my career at risk					
62	It is only necessary to report serious or unexpected ADRs					
63	I do not have time to complete an ADR report form					
64	I do not have the time to actively look for ADRs while at work					
65	I do not know how information reported in ADR form is used					
66	I talk with pharmaceutical companies about possible ADRs with their drugs					
67	I think that the best way to report ADRs is by publishing in medical literature					
68	I should be financially reimbursed for providing the ADR service					
69	I would be more likely to report ADRs if there were an easier method					

Part B: Medication Error Reporting System (MERS)

A **Medication Error** (ME) is defined as any error in the process of prescribing, dispensing, or administration of medication irrespective of whether such error leads to patient harm or not.

70. Do you think we should have a national system for reporting Medication Errors (MEs)?

[1] Yes [2] No

71. The Medication Error Reporting System should;

[1] be integrated with the ADR reporting system
[2] stand alone

72. Reporting of Medication Errors should be;

[1] Mandatory [2] Voluntary

73. Should medication error reporting relate only to fatal adverse events?

[1] Yes [2] No

74. Should patients participate in the reporting of MEs? [1] Yes [2] No

Instructions

In the left column are questions that will be the subject of your evaluation and in the right column is a gradual scale where you should mark with **X** the place along the scale where, according to your opinion, represents your degree of agreement with the text comment. The **extreme left** side indicates **total disagreement** while the **extreme right** indicates **total agreement**. Agreement increases as you move across from left to right
Please indicate whether you agree or disagree with the following statements

(1 = Strongly disagree; 2 = Slightly disagree; 3 = Neutral; 4 = Slightly agree; 5 = Strongly agree)

Strongly Disagree ⟷ Strongly Agree

	**Statement**	1	2	3	4	5
75	Reported MEs should be used to find the root causes of the MEs					
76	I would recommend a non-disciplinary approach to reporting to encourage health workers to report MEs					
77	There is lack of time for reporting MEs					
78	There is a culture of blame within healthcare					
79	There is need for organizational leadership and support in reporting MEs					
80	Personal details of the person who made an error are required to be reported					
81	The system should report both actual and potential MEs					
82	Medication errors are handled appropriately in this setting					
83	The culture in this health facility makes it easy to learn from the MEs of others					
84	I am more likely to make MEs in tense or hostile situations					
85	I have seen others make MEs that had the potential to harm patients					
86	Disruptions in continuity of patient care, such as shift changes, can be detrimental to patient safety					
87	I have made MEs that had the potential to harm patients					
88	Important issues are well communicated at shift changes					
89	I may hesitate to use a reporting system for MEs because I am concerned about being identified					
90	I should be financially rewarded for reporting MEs					
91	Minor medication errors should not be reported					
