# Supplementary material for: Rare, Serious, and Comprehensively Described Suspected Adverse Drug Reactions Reported by Surveyed Healthcare Professionals in Uganda
Source: PLoS One. 2015 Apr 23;10(4):e0123974. doi: 10.1371/journal.pone.0123974 (PMC4408100; doi:10.1371/journal.pone.0123974)
Supplement: S4 Appendix — (PDF) [file pone.0123974.s004.pdf]

Appendix S4: Survey-descriptions of 241 suspected Adverse Drug Reactions (ADRs) from 268 Healthcare Professionals (HCPs) who suspected ADRs in the past 4 weeks										
file	id	Level H/Facility	Type H/Facility	district	Region	Cadre	Nurse-cadre	HCP-Gender	HCP-Age	Serious ADR Description
		**Antibacterials Only**								
327	843	Private For-Profit	Health Centre III	KAMPALA	Central	Doctor		Male	39	35YR/FEMALE HYPERSENSITIVITY TO PENICILLINS
304	1653	Public	District Hospital	KAYUNGA	Central	Nurse	Registered Midwife	Female	42	28YRS, COTRIMOXAZOLE, SEVERE
466	464	Private For-Profit	Health Centre III	GULU	Other/NK	Other		Male	26	32YR/FEMALE KNOWN ISS PATIENT ON COTRIMOXAZOLE CAME WITH SEVERE BODY RASHES & SLOUGHING. 18YR/MALE GIVEN IV FORTIFIED PROCAINE PENICILLIN (PPF) & SHE
	1214	Public	National Referral	KAMPALA	Central	Doctor		Female	28	PATIENT REACTED TO IV CEFTRIAXONE GOT PRURITUS AND ANGIOEDEMA INVOLVING THE MANDIBULAR AREA
365	103	Private Not-for-Profit	Health Centre IV	BUIKWE	Central	Other		Male	27	20YR/FEMALE ON PENICILIN GOT MILD REACTION
	183	Public	National Referral	KAMPALA	Central	Nurse	Registered Nurse	Female	26	PATIENT WAS ON CIPRO AND GOT SWOLLEN HANDS
165	982	Private Not-for-Profit	Private Hospital	KAMPALA	Central	Doctor		Male	35	BODY ITCHING,SWELLING &DISCOMFORT DUE TO CEPHALEXIN CAPS
313	1662	Public	District Hospital	KAYUNGA	Central	Nurse	Registered Nurse	Female	34	MILD ADR.ELDERLY MALE PATIENT GOT ITCHING AFTER BENZYL PENICILIN BY IV ROUTE
417	1692	Private For-Profit	Health Centre IV	KAMPALA	Central	Nurse	Enrolled Nurse	Male	25	ADULT WITH UTI GIVEN ORAL CIPROFLOXIN GOT ARTHRALGIA, MODERATE ABDOMINAL PAIN,VOMITING & RESTLESSNESS. SEVERE ADR.
	20	Public	National Referral	KAMPALA	Central	Doctor		Male	26	HIV+ PATIENT REACTED TO SEPTRIN PROPHYLAXIS AND GOT GENERALIZED SKIN RASH
243	1254	Private Not-for-Profit	Other	KAMPALA	Central	Nurse	Registered Nurse	Male	33	,RISE IN BODY TEMPERATURE & ITCHING WHICH INTENSIFIED ON SWALLOWING SEPTRIN
279	80	Public	National Referral	KAMPALA	Central	Doctor		Male	35	LOOSE STOOL,BODY ITCHING & URTICARIA WITH BLISTERING FOLLOWING USE OF PENICILINS
909	1201	Private Not-for-Profit	Private Hospital	MBRA	Other/NK	Pharm		Female	32	60YR-OLD ON ORAL COTRIMOXAZOLE - RASHES,NAUSEA & SEVERE INVISIBLE SWEATS,MODERATE RXN
	1601	Public	National Referral	KAMPALA	Central	Doctor		Male	36	DRUG REACTIONS TO ORAL SEPTRIN
1043	1074	Public	Health Centre IV	TORORO	Eastern	Nurse	Registered Midwife	Female	35	28YR OLD PATIENT WAS ADMINISTERED WITH COTRIMOXAZOLE TABLETS DEVELOPED BLACK PATCHES ON SKIN - MODERATE
205	949	Public	Regional Referral	KAMPALA	Central	Doctor		Male	28	26YR ANAEMIC FEMALE WITH SEVERE PRE-ECLAMPSIA FINALLY DELIVERED & WAS MANAGED POST-OPERATIVELY WITH GENTAMICIN-SUSTAINED ACUTE RENAL FAILURE WITH ANAEMIA FOR 5 DAYS & OTHER COMPLICATIONS
325	2034	Private For-Profit	Private Hospital	WAKISO	Central	Nurse	Enrolled Comprehensive	Female		18YR/FEMALE REACTED TO INTRAVENOUS CEFTRIAXONE BY SWELLING OF HAND THROUGH WHICH DRUG WAS GIVEN & SEVERE BACK PAIN-MILD
1040	1071	Private For-Profit	Private Hospital	TORORO	Eastern	Doctor		Female	28	A MAN DIAGNOSED WITH HIV TOOK COTRIMOXAZOLE AND GOT STEVENS-JOHNSON SYNDROME - SEVERE
185	2006	Private For-Profit	Health Centre III	KAMPALA	Central	Other		Male	31	5YR OLD CHILD ON ORAL AMPICLOX (AMPICILLIN & CLOXACILLIN) DEVELOPED GENERALIZED RASHES. IT WAS MODERATE
450	447	Public	Health Centre III	GULU	Other/NK	Other		Male	30	ADULT FEMALE ON ORAL COTRIMOXAZOLE GOT SWELLING AND ITCHY FACE.MILD
1163	888	Private For-Profit	Other	MASAKA	Other/NK	Nurse	Enrolled Nurse	Female	28	23YR OLD,FLAGYL,ITCHING & NAUSEA,MILD
1061	1092	Public	District Hospital	TORORO	Eastern	Nurse	Nursing Assistant	Female	50	A 20YR OLD TAKING SEPTRIN ORALLY GOT SEVERE SKIN RASH ALL OVER THE BODY
809	708	Private Not-for-Profit	Private Hospital	JINJA	Eastern	Nurse	Nursing Assistant	Female	27	WOMAN ON SEPTRIN REACTED BY DEVELOPING GENERALIZED BODY RASH
750	649	Public	Health Centre IV	JINJA	Eastern	Other		Male		25YR/MALE REACTED TO IV AMPICILLIN.MODERATE.WAS GIVEN HYDROCORTISONE IV
74	42	Private Not-for-Profit	Private Hospital	KAMPALA	Central	Other		Male	26	30YR HIV+ MALE REACTED MODERATELY TO ORAL SEPTRIN PROPHYLAXIS
758	657	Public	Health Centre III	JINJA	Eastern	Pharm		Female	26	25YR OLD FEMALE ON CIPROFLOXACIN IV. GOT SJS.IT WAS SEVERE
274	2025	Public	National Referral	KAMPALA	Central	Doctor		Male	50	2YR OLD, CEFTRIAXONE, INTRAVENUS, MILD

606	824	Public	District Hospital	MASINDI	Other/NK	Other		Male	24	5YR OLD ON COTRIMOXAZOLE REACTED TO THE DRUG & WAS CHANGED TO CAP AMOXYCILLIN. MODERATE
94	923	Public	National Referral	KAMPALA	Central	Nurse	Other	Female	48	2.5YR OLD BOY REACTED TO SEPTRIN
957	142	Public	Health Centre III	MITOOMA	Other/NK	Other		Male	27	HIV+ PATIENT WITH BODY ACHES, SUDDEN SKIN RASH, BURNT SKIN, OOZING OF SWEAT-LIKE FLUID AFTER COTRIMOXAZOLE ADMINISTRATION - MILD SYMPTOMS
791	690	Private For-Profit	Health Centre IV	JINJA	Eastern	Doctor		Male	30	21YR PATIENT REACTED TO PPF WITH VOMITING.TREATMENT CHANGED TO ERYTHOMYCIN 500MG
919	1211	Private For-Profit	Health Centre III	MBRA	Other/NK	Doctor		Male	47	32YR OLD HIV+ MALE STARTED ON COTRIMOXAZOLE ORALLY. IT WAS SEVERE.RECOVERED
1096	1116	Public	District Hospital	TORORO	Eastern	Nurse	Registered Nurse	Female	29	IV/IM PENICILLINS - MODERATE RASHES AND ITCHING
343	1687	Private For-Profit	Health Centre III	KAMPALA	Central	Nurse	Enrolled Comprehensive	Female	20	26YR OLD REACTED TO SEPTRIN. MODERATE REACTION
	814	Public	National Referral	KAMPALA	Central	Doctor		Female	24	PATIENT GOT RASH AFTER TAKING SEPTRIN PROPHYLAXIS
550	2041	Public	National Referral	KAMPALA	Central	Other		Male	32	62YR/FEMALE ON COTRIMOXAZOLE ORAL ROUTE WITHIN TWO DAYS GOT MULTIPLE SKIN PATCHES, DEVELOPED SORES ON MUCOUS MEMBRANES WITH HIGH TEMPERATURE. GIVEN STEROIDS & SHE RECOVERED
597	815	Public	District Hospital	MASINDI	Other/NK	Other		Male	35	ADULT FEMALE GOT EXTENSIVE SKIN RASH,WAS ON STARTER SEPTRIN PROPHYLAXIS
484	1314	Private For-Profit	Private Hospital	KAMPALA	Central	Nurse	Registered Nurse	Female	35	ADULT PATIENT HAD ABDOMINAL CRAMPS & URTICARIA RASH DUE TO IV VANCOMYCIN - MODERATE
784	683	Private For-Profit	Health Centre IV	JINJA	Eastern	Nurse	Nursing Assistant	Male	25	29YR/FEMALE REACTED TO SEPTRIN - RASHES, GAVE HER DEXAMETHASONE AND PANADOL TO TREAT THE RASHES
587	805	Public	District Hospital	MASINDI	Other/NK	Nurse	Registered Midwife	Female	32	SEPTRIN
876	1168	Public	Health Centre III	MBRA	Other/NK	Other		Male	32	COTRIMOXAZOLE ORAL, MILD ADR
608	826	Public	District Hospital	MASINDI	Other/NK	Other		Male		28YR/MALE - ALLERGIC REACTION TO ORAL CIPROFLOXACIN - SEVERE URTICARIA - SUBSTITUTED FOR CEPHALEXIN & CETIRIZINE. 30YR-OLD FEMALE REACTED TO NEVIRAPINE - MODERATE & WAS SUBSTITUTED FOR EFV
	1618	Public	National Referral	KAMPALA	Central	Doctor		Male	26	TWO PATIENTS GOT ITCHY RASH 20 MINUTES AFTER TRAMADOL INJECTION, 1 PATIENT DEVELOPED ITCHY RASH AFTER IM DICLOFENAC, 1 PATIENT GOT RASH AFTER IV CIPROFLOXACIN
991	1023	Private Not-for-Profit	Private Hospital	TORORO	Eastern	Nurse	Enrolled Comprehensive	Male	20	A MAN 35YRS WITH GENERALIZED SKIN RASH AFTER TAKING ORAL SEPTRIN - SEVERE
76	48	Private Not-for-Profit	Private Hospital	KAMPALA	Central	Pharm		Male	27	40YR MALE REACTED TO PENICILINS (FLUCLOXACILLIN-AMOXICILLIN COMBINATION) - GOT RASHES AFTER FIRST DOSE, REACTION WAS MODERATE
53	906	Private For-Profit	Private Hospital	KAMPALA	Central	Doctor		Male	45	REDUCED BLOOD SUGAR & LOW PULSE AFTER TAKING CHLORAMPHENICOL INJECTION
396	854	Private For-Profit	Private Hospital	KAMPALA	Central	Pharm		Female	24	REACTION TO CEFTRIAXONE WITH MILD INFLAMATION &SWELLING AT POINT OF INJECTION. REACTION TO CIPROFLOACIN WITH SWELLING OF TONGUE.
349	86	Private For-Profit	Health Centre IV	BUIKWE	Central	Nurse	Enrolled Comprehensive	Male	25	58YR OLD ON ORAL COTRIMOXAZOLE GOT SEVERE URTICARIA AND SKIN RASHES ALL OVER THE BODY
904	1196	Public	Health Centre IV	MBRA	Other/NK	Nurse	Registered Nurse Midwif	Female	50	29YR-OLD ON COTRIMOXAZOLE ORALLY, REACTION WAS MODERATE
990	1022	Private Not-for-Profit	Private Hospital	TORORO	Eastern	Other		Female	25	38YR/MALE ON SEPTRIN DEVELOPED SJS - MODERATE
1002	1033	Private For-Profit	Health Centre IV	TORORO	Eastern	Other		Male	27	8YR OLD BOY DEVELOPED ITCHY BODY RASH AND SWOLLEN EYES AFTER RECEIVING BENZYL PENICILLIN - MODERATE. RESOLVED WITHIN 1HR AFTER GIVING HYDROCORTISONE

523	496	Public	Regional Referral	LIRA	Other/NK	Doctor		Female	35	80YR OLD WOMAN REACTED TO COTRIMOXAZOLE.SKIN CHANGED BUT WAS A MILD CASE
331	847	Private For-Profit	Private Hospital	KAMPALA	Central	Pharm		Male	30	24YR PATIENT REACTED TO PENICILLIN ORAL ROUTE,GOT GENERALIZED RASH AND BODY ITCHING
61	914	Public	National Referral	KAMPALA	Central	Doctor		Male	28	REACTION TO SEPTRIN PROPHYLAXIS IN A NEWLY DIAGNOSED HIV PATIENT STARTING SEPTRIN DOSE
154	1868	Private For-Profit	Health Centre IV	KAMPALA	Central	Other		Male	29	9YR OLD MALE GOT SKIN RASH AFTER TAKING ORAL PENICILLIN.ONSET WAS SUDDEN BUT RESOLVED AFTER USE OF TOPICAL HYDROCORTISONE CREAM
1058	1089	Public	District Hospital	TORORO	Eastern	Nurse	Registered Nurse	Female	50	30YR OLD - SULPHUR REACTION-ORAL COTRIMOXAZOLE, SKIN RASH - MILD ; 40YR OLD NEVIRAPINE REACTION - SEVERE
614	832	Public	District Hospital	MASINDI	Other/NK	Nurse	Registered Nurse Midwif	Female	43	40YRS,SEPTRIN,ORALLY.SJS
465	463	Private For-Profit	Private Hospital	GULU	Other/NK	Other		Female	32	72YR/FEMALE REACTED TO BENZYL PENICILLIN WITH SHIVERS,ITCHY BODY RASHES & PROFUSE SWEATING IMMEDIATELY AFTER INJECTION - MODERATE
700	598	Private For-Profit	Drug Shop	KAMULI	Eastern	Nurse	Enrolled Nurse	Female	28	OLD WOMAN REACTED TO SEPTRIN,GENERALIZED BODY SORES,GAVE HER PREDISOLONE & PAIN KILLER PLUS BETADERM TOPICAL (BETAMETHASONE)
953	138	Public	Health Centre III	SHEEMA	Other/NK	Other		Male	38	FEMALE 39YRS IV CEFTRIAXONE - MILD
1008	1039	Private For-Profit	Private Hospital	TORORO	Eastern	Nurse	Nursing Assistant	Female	20	18YR OLD GIVEN IV CEFTRIAXONE. DRUG INJECTED VERY FAST & THE PATIENT DEVELOPED SEVERE VOMITING
724	623	Private For-Profit	Drug Shop	KAMULI	Eastern	Other		Male	36	PATIENT ON SEPTRIN ORAL ROUTE,DEVELOPED RASH ALL OVER BODY, TREATMENT WAS STOPPED
411	1371	Private For-Profit	Health Centre IV	KIRYANDO	Other/NK	Nurse	Enrolled Comprehensive	Male	32	28YR OLD ON COTRIMOXAZOLE TAB TAKEN ORALLY,MODERATE ADR (HYPERSENSITIVITY)
738	637	Public	Health Centre III	JINJA	Eastern	Nurse	Enrolled Nurse	Female	20	26YR/MALE HIV+ ON ORAL SEPTRIN,GOT BURNT FACE & LIPS-GIVEN ORAL DEXAMETHASONE FOR FIVE DAYS - SEVERE
87	1626	Public	National Referral	KAMPALA	Central	Doctor		Male	30	1YR OLD GOT MILD REACTION TO COTRIMOXAZOLE FOR URTI
	14	Public	National Referral	KAMPALA	Central	Doctor		Male	27	SKIN REACTIONS FOLLOWING IV CEFTRIAXONE
329	845	Private Not-for-Profit	Health Centre III	KAMPALA	Central	Other		Male	26	30YR/MALE ON ORAL TINIDAZOLE GOT HEADACHE & DIZZINESS.IT WAS MILD
	2011	Private For-Profit	Health Centre III	WAKISO	Central	Doctor		Female	30	CHILD ON IV CIPROFLOXACIN GOT BODY ITCH, SWELLING AROUND THE FACE & AROUND SITE OF THE INJECTION
182	1263	Private Not-for-Profit	Other	KAMPALA	Central	Doctor		Female	25	24 YR PATIENT, VOMITING AFTER ORAL DOXYCYCLINE, MODERATE
59	912	Public	National Referral	KAMPALA	Central	Doctor		Male	28	VOMITING,IV CEFTRIAXONE-MODERATE
		**Antiretrovirals Only**								
183	818	Public	National Referral	KAMPALA	Central	Nurse	Registered Nurse	Female	32	50YR FEMALE PATIENT GOT NEVIRAPINE (ORAL) HYPERSENSITIVITY INVOLVING ALL MUCUS MEMBRANES,SEVERE BUT RESOLVED
69	922	Public	National Referral	KAMPALA	Central	Nurse	Registered Nurse Midwif	Female		30YR PATIENT REACTED TO ART ADMINISTERED ORALLY. PATIENT LOST SKIN & MUCUS MEMBRANES-SEVERE
191	2004	Public	National Referral	KAMPALA	Central	Doctor		Female	32	36YR MALE ADMITTED WITH SJS FOLLOWING INITIATION OF ORAL NEVIRAPINE,SEVERE EVENT. 17YR OLD FEMALE ON ORAL AZITHROMYCIN GOT SKIN RASHES NECESSITATING ADMISSION-MODERATE
192	2005	Public	National Referral	KAMPALA	Central	Other		Female	30	SKIN RASH IN A 45YR FEMALE PATIENT ON CBV/EFV/CTX FOR 2YRS TAKEN ORALLY. MODERATE. WAS REFFERED TO DERMATOLOGIST
	825	Public	National Referral	KAMPALA	Central	Doctor		Male	38	PT ON ALUVIA (LOPINAVIR & RITONAVIR) COMPLAINED OF DRY MOUTH AND THROAT DISCOMFORT
	1807	Public	National Referral	KAMPALA	Central	Doctor		Male	29	ISS PATIENT WITH SKIN RASH,SCALING,ULCERATIONS FOR 2WKS THAT STARTED 1WK AFTER ART INITIATION OF COMBIVIR-NEVIRAPINE (AZT/3TC/NVP)

39	1625	Public	National Referral	KAMPALA	Central	Doctor		Male	28	PATIENT ON POST-EXPOSURE-PROPHYLAXIS (CBV/EFV) PRESENTS WITH EUPHORIA & DECREASED SLEEP: EFV-INDUCED NEUROPSYCHIATRIC MOOD
	2019	Private Not-for-Profit	Private Hospital	WAKISO	Central	Doctor		Male	27	HIV+/FEMALE NEWLY ENROLLED ON HAART WITH NVP-BASED REGIMEN DEVELOPED SJS WHICH WAS SEVERE
1159	884	Public	Regional Referral	MASAKA	Other/NK	Pharm		Female		ORAL NEVIRAPINE, MODERATE REACTION, DRUG WITHHELD & PATIENT STABILIZED
403	1287	Public	Health Centre III	KAMPALA	Central	Doctor		Female	39	PERIPHERAL NEUROPATHY DUE TO AZT
370	109	Private Not-for-Profit	District Hospital	BUIKWE	Central	Nurse	Registered Nurse	Female	25	MILD ADR.PATIENT ON ORAL EFV HAD GYNAECOMASTIA AND JERKS
1034	1065	Private Not-for-Profit	District Hospital	TORORO	Eastern	Nurse	Registered Nurse	Male	25	MOTHER REPORTED CONVULSIONS WHENEVER THE CHILD TOOK HIS ORAL ARVS - MODERATE
486	1289	Public	National Referral	KAMPALA	Central	Doctor		Female	29	4YR OLD,EFAVIRENZ-ORAL ROUTE-MODERATE ADR
402	1273	Private For-Profit	Private Hospital	KAMPALA	Central	Pharm		Male	25	30YR/FEMALE ON AZT/3TC/NVP GOT BODY RASH &ITCHING AFTER 2WEEKS.MODERATE ADR
887	1179	Private For-Profit	Private Hospital	MBRA	Other/NK	Pharm		Male	30	35YRS ORAL EFV LED TO MODERATE MENTAL DISTURBANCE, SUBSTITUTN REDUCED IT
409	1368	Private For-Profit	Private Hospital	KIRYANDO	Other/NK	Other		Female	27	35YR/FEMALE, WITH CD4 100 CELLS/DL ON AZT/3TC/NVP,AFTER 2WEEKS SHE CAME BACK WITH A SEVERE GENERALIZED BODY RASH
1157	882	Public	Health Centre III	MASAKA	Other/NK	Other		Female	27	32YR OLD REACTED TO ORAL NEVIRAPINE - SJS. REACTION WAS MODERATE
63	916	Private Not-for-Profit	Health Centre IV	KAMPALA	Central	Doctor		Female	32	NVP SKIN HYPERSENSITIVITY
430	426	Private Not-for-Profit	Private Hospital	GULU	Other/NK	Nurse	Registered Nurse	Female	36	27YR/FEMALE ON PEP ORAL ROUTE GOT BLISTERS 3 DAYS LATER WITH BURNING SENSATION ALL-OVER THE BODY.MODERATE
	402	Public	National Referral	KAMPALA	Central	Pharm		Male		SKIN RASH DUE TO NEVIRAPINE TABLETS
1057	1088	Public	District Hospital	TORORO	Eastern	Nurse	Registered Nurse	Female	48	32YR/FEMALE ISS PATIENT ON AZT/3TC/NVP FOR 3 MONTHS GOT SKIN RASH ALL OVER THE BODY - STEVEN JOHNSON SYDROME - SEVERE
723	622	Private For-Profit	Drug Shop	KAMULI	Eastern	Doctor		Male	28	27YR/FEMALE ON ORAL DUOVIR-N (AZT/3TC/NVP) DEVELOPED SWELLING & ITCHING. WAS MODERATE
495	863	Private Not-for-Profit	Private Hospital	KAMPALA	Central	Doctor		Male	24	38YR OLD FEMALE WITH SEVERE NEVIRAPINE HYPERSENSITIVITY
628	526	Public	District Hospital	KAMULI	Eastern	Other		Male	30	37YR OLD GOT SJS AFTER TAKING ARVS. WAS LATER GIVEN IV FLUID ANTIBIOTICS, STEROID DRUGS & SUPPORTIVE TREATMENT
1009	1040	Private For-Profit	Other	TORORO	Eastern	Other		Male	32	45YR/FEMALE DEVELOPED DEEP JAUNDICE (HEPATOTOXICITY) AFTER RECEIVING ORAL NEVIRAPINE. WAS GIVEN EFV AS ALTERNATIVE DRUG AND SHE IMPROVED
187	2008	Public	District Hospital	KAMPALA	Central	Doctor		Female	33	28YR FEMALE ON ORAL DUOVIR-N (AZT/3TC/NVP), GOT RASH WHICH WORSENED & SHE DEVELOPED SJS
910	1202	Private For-Profit	Private Hospital	MBRA	Other/NK	Pharm		Male	27	24 YR-OLD WITH SKIN PEELING OFF DUE TO NEVIRAPINE, REACTION WAS SEVERE
326	2035	Private For-Profit	Private Hospital	WAKISO	Central	Doctor		Female		ORAL ADMINISTRATION OF NEVIRAPINE IN PATIENT LEADING TO HEPATOTOXITY
506	1013	Private Not-for-Profit	Private Hospital	WAKISO	Central	Other		Male	33	26YR OLD ON ARVS (AZT+3TC-NVP) MODERATE ITCHY SKIN RASH-RESOLVED AFTER DISCONTINUATION OF ART. 16YR OLD WITH URTICARIA DUE TO AMOXICILLIN - MILD ADR. WAS GIVEN HYDROCORTISONE.
563	781	Public	District Hospital	MASINDI	Other/NK	Other		Male	25	27YR OLD WITH 237 CD4 CELLS/ML ON NEVIRAPINE ORAL ROUTE GOT SEVERE RASH
170	64	Private For-Profit	Private Hospital	KAMPALA	Central	Doctor		Male	24	25YR-OLD ANAEMIC, AZT
987	1019	Private Not-for-Profit	Private Hospital	TORORO	Eastern	Nurse	Enrolled Comprehensive	Female	22	38YR OLD IMMEDIATELY AFTER STARTING ORAL ARVS FELT LIKE HOT WATER HAD BEEN POURED ON HIM - SEVERE
626	524	Public	District Hospital	KAMULI	Eastern	Nurse	Enrolled Nurse	Female	24	42YR OLD WOMAN REACTED TO ARVS,TREATED WITH ANTIBIOTICS, THAT IS, CEFTRIAXONE
1165	890	Public	Regional Referral	MASAKA	Other/NK	Other		Female		NVP RASH

951	136	Public	Health Centre IV		Other/NK	Pharm		Female	27	35 YR-OLD ISS PATIENT ON AZT/3TC/NVP GOT SKIN RASH MORE SEVERE ON LOWER LIMBS & SKIN STARTED PEELING OFF,DID NOT RESPOND TO STEROIDS OR ANTIFUNGALS
1067	1098	Public	District Hospital	TORORO	Eastern	Doctor		Female	28	4YR HIV+ CHILD ON PROTEASE INHIBITORS SWITCHED TO ORAL EFV - SEVERE BEHAVIORAL DISTURBANCE INCLUDING AGGRESSION, INSOMNIA & HYPERACTIVITY
786	685	Private For-Profit	Other	JINJA	Eastern	Other		Male	29	ADR WAS DUE TO NEVIRAPINE WHILE PATIENT RECEIVED ART
123	1839	Private For-Profit	Private Hospital	KAMPALA	Central	Other		Male	27	ORAL NEVIRAPINE INDUCED RASH-SJS IN 26YR PATIENT. REACTION TO EFAVIRENZ CAUSING AUDITORY & VISUAL HALLUCINATIONS
471	469	Public	National Referral	GULU	Other/NK	Nurse	Registered Nurse	Female	40	3OYR ON AZT ORAL ROUTE GOT BODY RASH,FEVER, & ITCHING- MODERATE
423	419	Private Not-for-Profit	Regional Referral	GULU	Other/NK	Pharm		Female	26	22YR/FEMALE LADY NURSE WHO GOT NEEDLE STICK INJURY & WAS INITIATED ON PEP WITH AZT/3TC-12 DAYS LATER GOT SEVERE RASH (SJS) & WAS ADMITED AND TREATED
612	830	Public	District Hospital	MASINDI	Other/NK	Nurse	Registered Nurse	Female	33	45YR PATIENT ON ORAL TDF/3TC/NVP GOT SEVERE ABDOMINAL PAIN WHICH WAS IN COLICKY FORM
613	831	Public	District Hospital	MASINDI	Other/NK	Other		Female	36	39YR/FEMALE ON ZIDOVIDINE ORAL ROUTE GOT ANAEMIA. SHE WAS BLOOD TRANSFUSED & AZT WAS REPLACED WITH TENOFOVIR
1161	886	Public	Regional Referral	MASAKA	Other/NK	Other		Female	42	NVP RASHES, AZT ANEMIA, ATAZANAVIR HEPATOTOXICITY JAUNDICE
1158	883	Public	Regional Referral	MASAKA	Other/NK	Nurse	Registered Comprehensiv	Female		ORAL NEVIRAPINE, SEVERE HEADACHE, PATIENT STABILIZED
603	821	Public	District Hospital	MASINDI	Other/NK	Other		Male	38	35YR ON AZT-CONTAINING REGIMEN ORAL ROUTE DEVELOPED SEVERE ANAEMIA LEADING TO ADMISSION & BLOOD TRANSFUSION
		**Antimalarials Only**								
992	1024	Private Not-for-Profit	Private Hospital	TORORO	Eastern	Nurse	Enrolled Nurse	Female	27	A CHILD OF 5YRS AFTER INTRAVENOUS QUININE. SHE DIED AFTER ARRIVAL
152	1866	Private For-Profit	Health Centre IV	KAMPALA	Central	Other		Male	34	SWELLING OF FACE IN 16YR PATIENT AFTER SWALLOWING COARTEM (ORALLY)-MODERATE
567	785	Private Not-for-Profit	Private Hospital	MASINDI	Other/NK	Nurse	Enrolled Comprehensive	Female	23	4YR OLD ON COARTEM ORAL ROUTE. MODERATE REACTION
	175	Public	Health Centre III	TORORO	Eastern	Nurse	Registered Nurse	Female	25	ITCHING OF SKIN AFTER TAKING ORAL CHLOROQUINE, ANOTHER GOT RASH AFTER TAKING ORAL COARTEM
682	580	Private For-Profit	Drug Shop	KAMULI	Eastern	Nurse	Nursing Assistant	Male	40	55YR OLD MALE ON QUININE TABS ORAL ROUTE GOT BODY ITCHING
783	682	Private For-Profit	Drug Shop	JINJA	Eastern	Nurse	Enrolled Nurse	Female	18	MAN REACTED TO FANSIDAR IV-VOMITING.TREATMENT WAS PLASIL AND WATER
522	495	Public	Regional Referral	LIRA	Other/NK	Nurse	Registered Nurse	Female	27	26YR/MALE ON COARTEM ORAL ROUTE,RASH ON BOTH HANDS, ITCHING & SWELLING
705	603	Private For-Profit	Drug Shop	KAMULI	Eastern	Nurse	Nursing Assistant	Female	26	CHILDREN REACTED TO CHLOROQUINE WITH BODY ITCHING,WERE GIVEN DEXAMETHASONE TO STOP THE REACTION
869	767	Public	Regional Referral	JINJA	Eastern	Nurse	Registered Nurse	Female	32	3YR OLD CHILD GIVEN IV QUININE & SHE BECAME HYPOGLYCAEMIC
799	698	Private Not-for-Profit	Health Centre IV	JINJA	Eastern	Doctor		Male	24	52YR OLD FEMALE ON COARTEM ORAL ROUTE DEVELOPED SORES ON THE WHOLE BODY
986	1017	Private Not-for-Profit	Private Hospital	TORORO	Eastern	Nurse	Enrolled Comprehensive	Male	24	2YR OLD GIVEN IM QUININE DEVELOPED HYPOGLYCAEMIA - IMPROVED AFTER MANAGEMENT - SEVERE
82	54	Private Not-for-Profit	Private Hospital	KAMPALA	Central	Pharm		Male	34	MILD SULPHUR REACTION TO ORAL TREATMENT WITH SULFADOXINE-PYRIMETHAMINE (SP) AGE-ADULT
624	522	Public	District Hospital	KAMULI	Eastern	Nurse	Enrolled Nurse	Male	47	2YR OLD BOY WAS BROUGHT TO HOSPITAL WITH A REACTION TO QUININE & DOCTOR GAVE HIM AN ANTIDOTE BUT HE DIED
914	1206	Private Not-for-Profit	Private Hospital	MBRA	Other/NK	Nurse	Enrolled Nurse	Female	60	17YR-OLD ON IM QNN WITH POST INJECTION PARALYSIS, IT WAS SEVERE

26	1810	Private For-Profit	Health Centre III	KAMPALA	Central	Doctor		Male	35	TINNITUS IN A 27YR-OLD AFTER IV QUININE. SEVERE (LED TO ALTERED CONSCIOUSNESS).
771	670	Private For-Profit	Pharmacy	JINJA	Eastern	Pharm		Female	35	PATIENT REACTED TO QUININE WITH ITCHING, TREATED IT WITH CETIRIZINE
888	1180	Private Not-for-Profit	Private Hospital	MBRA	Other/NK	Nurse	Enrolled Nurse	Female	50	PATIENT ON ORAL QNN GOT SKIN RASH,TINNITUS,ABORTION,VERTIGO, NAUSEA, VOMITING, BLURRED VISION - SEVERE
707	605	Private For-Profit	Health Centre IV	KAMULI	Eastern	Nurse	Nursing Assistant	Female	21	25YR ON QUININE ORAL ROUTE, MILD, ADVISED HIM TO DRINK A LOT OF FLUIDS & TO TAKE PAIN KILLERS
1073	1104	Private For-Profit	Other	TORORO	Eastern	Other		Male	38	A FOUR-AND-A-HALF-YEAR-OLD CHILD TOOK CHLOROQUINE & DEVELOPED SKIN RASHES - MODERATE
570	788	Private For-Profit	Health Centre IV	MASINDI	Other/NK	Doctor		Male	62	62YR/FEMALE ON ORAL MEPHAQUINE GOT SEVERE HEADACHE WITH MENTAL CONFUSION & INSOMNIA
851	749	Private For-Profit	Drug Shop	JINJA	Eastern	Nurse	Registered Midwife	Male	30	28YR OLD FEMALE REACTED TO QUININE IV LEADING TO MISCARRIAGE
259	987	Private For-Profit	Health Centre III	KAMPALA	Central	Other		Male	32	28YR/FEMALE GOT BODY ITCHING AFTER TAKING ORAL QUININE. ADR WAS MODERATE
398	856	Private For-Profit	Private Hospital	KAMPALA	Central	Doctor		Female	34	PATIENT PUT ON IV ARTHEMETHER,GOT GENERALIZED ITCHY SKIN RASH ABOUT 1HOUR AFTER INJECTION. WAS MILD
5	34	Private Not-for-Profit	Health Centre IV	KAMPALA	Central	Nurse	Registered Midwife	Female	26	VOMITING & IRRITABILITY AFTER IV QUININE ADMINISTRATION BUT LATER STABILISED. REACTIONS WERE MILD.
49	902	Public	Health Centre IV	KAMPALA	Central	Doctor		Male		SEVERE HYPOGLYCEMIA IN ADULT-DRUG WAS ARTESUNATE/AMODIAQUINE
359	97	Private Not-for-Profit	Other	BUIKWE	Central	Doctor		Male	52	PATIENT 52YR B/S +VE FOR MALARIA PARASITES. GIVEN COARTEM FOR THREE DAYS' TREATMENT, AFTER 1 DAY GOT SKIN ITCHING & SEVERE RASH.STOPPED DRUG, GAVE IV QNN & PATIENT IMPROVED
1109	1129	Public	District Hospital	TORORO	Eastern	Doctor		Female	27	9-MONTH-OLD BABY ON ORAL COARTEM - VOMITING BUT RESOLVED. MILD REACTION
1052	1083	Public	Health Centre IV	TORORO	Eastern	Other		Male	24	36YR OLD ON ORAL FANSIDAR (SULPHADOXINE/PYRIMETHAMINE) DEVELOPED HYPERPIGMENTATION OF THE SKIN & THE THROAT - MILD
		**Analgesics Only**								
278	2029	Private For-Profit	Private Hospital	KAMPALA	Central	Doctor		Male	28	ORAL DICLOFENAC 50MG, HAEMOPTYSIS AFTER 2 DAYS - WAS SEVERE. DUOCOTEXCIN - VOMITING UNNECESSARILY
	176	Private Not-for-Profit	Private Hospital	KAMPALA	Central	Doctor		Male	30	SKIN ITCHING SECONDARY TO OPIOID USE
171	65	Private Not-for-Profit	District Hospital	KAMPALA	Central	Nurse	Registered Nurse Midwife	Female	38	28YR FEMALE PATIENT GIVEN IM MORPHINE, GOT GENERALIZED SKIN ITCHING OF MODERATE SEVERITY
332	1672	Private For-Profit	Private Hospital	KAMPALA	Central	Other		Male	30	ORAL ROUTE OF TRAMADOL RESULTED INTO SEVERE ITCHY SORES ALL-OVER THE BODY THAT PERSISTED
390	848	Private For-Profit	Other	KAMPALA	Central	Doctor		Female	60	MILD DYSPEPSIA FROM ADULTS USING ORAL ANALGESICS FOR ATHRITIS
878	1170	Private For-Profit	Private Hospital	MBRA	Other/NK	Nurse	Registered Comprehensiv	Female	23	23YR MALE TOOK IBUPROFEN GOT GENERALIZED SKIN RASH & ITCHING ALL-OVER THE BODY; IT WAS MODERATE. TREATED WITH IV HYDROCORTISONE
574	792	Private Not-for-Profit	Health Centre IV	MASINDI	Other/NK	Nurse	Enrolled Comprehensive	Male	22	25YR OLD ON IV TRAMADOL 100MG, DEVELOPED PALPITATIONS & SWEATING BUT GOT BETTER AFTER 20 MINUTES - MILD
175	69	Private Not-for-Profit	District Hospital	KAMPALA	Central	Doctor		Male		IV PETHIDINE GIVEN TWICE CAUSED A SKIN REACTION
511	484	Public	Regional Referral	LIRA	Other/NK	Pharm		Male	38	26YR/FEMALE HAD A HISTORY OF PEPTIC ULCER DISEASE - DUE TO INDOMETHACIN BUT WAS NOT SEVERE
725	624	Private For-Profit	Health Centre IV	KAMULI	Eastern	Nurse	Enrolled Nurse	Female	56	16YR OLD BOY TOOK OVERDOSE OF IBRUPROFEN (20 TABLETS).PUT HIM ON A DRIP & HE RECOVERED.

927	1219	Private For-Profit	Pharmacy	MBRA	Other/NK	Pharm		Male	25	GASTROINTESTINAL DISTRESS DUE TO NSAID-INDUCED ULCERATION AFTER ORAL DICLOFENAC
453	450	Public	National Referral	GULU	Other/NK	Doctor		Female	35	41YR/MALE GIVEN PANADOL (PARACETAMOL), STARTED SHAKING & SWEATING 10 MINUTES LATER.WAS PUT ON A DRIP AND HE BECAME FINE.
518	491	Public	Regional Referral	LIRA	Other/NK	Doctor		Female	40	10YR/MALE REACTED TO PANADOL (PARACETAMOL) WHEN TOOTH WAS REMOVED,BECAME DIZZY-MINOR
1038	1069	Private For-Profit	Private Hospital	TORORO	Eastern	Nurse	Registered Comprehensiv	Female	29	65YR/FEMALE PATIENT'S BLOOD PRESSURE LOWERED FROM 105/60 TO 86/50 MMHG & STARTED SWEATING AT A TEMPERATURE OF 35.0 DEGREES CELSIUS DUE TO DYNAPAR (IV DICLOFENAC INFUSION) - MILD
	16	Public	National Referral	KAMPALA	Central	Pharm		Male	25	PATIENT DEVELOPED PEPTIC ULCER DISEASE A FEW DAYS AFTER TAKING ACECLOFENAC
		**Antituberculosis drugs Only**								
502	1008	Private Not-for-Profit	Private Hospital	KAMPALA	Central	Nurse	Enrolled Comprehensive	Male	30	40YR OLD ON ETHAMBUTOL ORAL ROUTE GOT BURNING SENSATION OF BOTH LIMBS & LOSS OF SIGHT - MODERATE SEVERITY
	1611	Public	National Referral	KAMPALA	Central	Doctor		Male	26	GENERALIZED BODY RASH FOLLOWING USE OF ANTI-TBS
		**Antibacterials & Antiretrovirals**								
949	134	Public	Health Centre IV		Other/NK	Nurse	Registered Nurse	Male		WOMAN AGED 30YRS ON NEVIRAPINE & COTRIMOXAZOLE WHO DEVELOPED A SKIN RASH
		**Antibacterials & Antimalarials**								
160	1874	Private For-Profit	Health Centre IV	KAMPALA	Central	Doctor		Female		19YR FEMALE PATIENT WITH MALARIA & COUGH REACTED SEVERELY TO COMBINATION OF QNN-IV & ORAL SEPTRIN. MANAGED WITH PARENTERAL HYDROCORTISONE
996	1027	Private Not-for-Profit	Private Hospital	TORORO	Eastern	Nurse	Enrolled Comprehensive	Female	30	5YR OLD GIRL FROM A CLINIC WHERE SHE WAS PUT ON IV QUININE & SEPTRIN. GOT BLISTERS ALL OVER THE BODY & DIED ON ADMISSION - SEVERE
988	1020	Private Not-for-Profit	Private Hospital	TORORO	Eastern	Nurse	Enrolled Comprehensive	Female	27	8YR OLD GIRL REFERRED FROM A CLINIC AFTER RECEIVING IV QUININE & SEPTRIN DEVELOPED BLISTERS ALL-OVER THE BODY & DIED ON ADMISSION - SEVERE
1047	1078	Private Not-for-Profit	Private Hospital	TORORO	Eastern	Nurse	Nursing Assistant	Female	29	5YR OLD GIRL WAS REFERRED FROM A CLINIC AFTER TAKING IV QUININE & SEPTRIN WITH BLISTERS ALL-OVER THE BODY. SHE DIED ON ADMISSION - SEVERE
		**Antiretrovirals & Antituberculosis drugs**								
194	984	Public	National Referral	KAMPALA	Central	Nurse	Registered Nurse	Female	37	37YR FEMALE PATIENT ON ORAL ARVS/TB GOT ENTIRE BODY RASH
		**Antibacterials and Antituberculosis drugs**								
253	994	Public	National Referral	KAMPALA	Central	Nurse	Registered Nurse Midwife	Female	60	ADULT SEVERELY REACTED TO SEPTRIN AND TB DRUGS TAKEN ORALLY
197	941	Private Not-for-Profit	Private Hospital	KAMPALA	Central	Doctor		Male	26	24YR/FEMALE KNOWN IMMUNOSUPPRESSED SYNDROME (ISS) PATIENT ON ANTI-TBS WHO REACTED TO COTRIMOXAZOLE - SJS & ALSO HAD TOXOPLASMOSIS
301	1650	Private For-Profit	Health Centre III	KAYUNGA	Central	Nurse	Enrolled Nurse	Female		ELDERLY PATIENT ON PIROXICAM COMPLAINED OF HEART PAIN WHICH INDICATED PUD & ALSO GOT OEDEMA DUE TO PENICILLINS
227	842	Public	National Referral	KAMPALA	Central	Doctor		Male	30	30YR/FEMALE PATIENT GIVEN IM MORPHINE & IV CEFTRIAXONE AFTER CAESARIAN-SECTION. GOT GENERALIZED BODY RASH & ITCHING 15MIN LATER
		**Antiseptic & Anaesthetic**								
163	1877	Public	National Referral	KAMPALA	Central	Doctor		Male	34	28YR FEMALE REACTED TO ORACURE GEL (LIGNOCAINE & CETYLPYRIDINIUM) FOR ORAL SORES-GOT SWOLLEN LIPS/TONGUE
		**Antibacterial & Antiviral**								

913	1205	Private Not-for-Profit	Private Hospital	MBRA	Other/NK	Doctor		Male	41	74YR-OLD WITH CORNEAL ULCER NOT RESPONDING TO TOPICAL ANTIBIOTICS & ANTIVIRALS FOR 14 DAYS. STOPPED THE MEDICATION AND LEFT PATIENT ON ARTIFICIAL TEARS ONLY & NOTED GREAT IMPROVEMENT
		**Antiretroviral & Analgesic**								
	19	Public	National Referral	KAMPALA	Central	Pharm		Male	24	OVER DOSAGE OF NSAIDS AND PROTEASE INHIBITORS FROM PHYSCIANS WHO DONT CONSULT PHARMARCISTS
		**Antibacterial & Blood**								
	1225	Public	National Referral	KAMPALA	Central	Doctor		Male	25	HYPERSENSITIVITY REACTION TO SEPTRIN & BLOOD
		**Other**								
110	1252	Public	National Referral	KAMPALA	Central	Nurse	Enrolled Midwife	Female	51	PROLONGED HEAVY FLOW,INCREASED BLOOD PRESSURE & PAIN OF ARM WHILE USING IMPLANTS
1162	887	Public	Health Centre IV	MASAKA	Other/NK	Pharm		Male	30	40YR OLD SEVERE HEADACHE AFTER ORAL LOSARTAN
429	425	Private Not-for-Profit	Private Hospital	GULU	Other/NK	Nurse	Enrolled Comprehensive	Male	26	13YR/MALE ON PREDNISOLONE ORAL ROUTE GOT SEVERE EPIGASTRIC PAIN WHICH WAS MANAGED AS SEVERE STEROID-INDUCED GASTRITIS
	808	Private For-Profit	Health Centre III	KAMPALA	Central	Other		Male	24	MALE ADULT DEVELOPED ERECTILE DYSFUNCTION DUE TO NIFEDIPINE
104	983	Public	National Referral	KAMPALA	Central	Nurse	Registered Nurse Midwife	Female	54	ALCOHOL 95% ADMINISTERED IN THE EYE OF A PATIENT
710	608	Private For-Profit	Health Centre IV	KAMULI	Eastern	Nurse	Nursing Assistant	Female	28	30 YR FEMALE REACTED TO INJECTAPLAN,OVER BLEEDING. I PRESCRIBED IBRUPROFEN & AMOXICILLIN FOR FOUR DAYS & BLEEDING STOPPED
	833	Public	National Referral	KAMPALA	Central	Nurse	Registered Nurse Midwife	Female	31	SEVERE PALPITATIONS DUE TO OVER DOSE OF OMEPRAZOLE
	1241	Public	National Referral	KAMPALA	Central	Nurse	Registered Nurse	Female	45	PATIENT RECEIVED IV METHOTREXATE, ADRIANYCIN & VINCRISTINE GOT SEVERE DIARRHOEA, ORAL STOMATITIS (NEUTROPENIC)
1050	1081	Private Not-for-Profit	Private Hospital	TORORO	Eastern	Nurse	Enrolled Midwife	Female	40	3-MONTH-OLD BABY WAS INJECTED WITH DPT AND GOT A REDENNING OF INJECTION SITE
234	978	Private For-Profit	Other	KAMPALA	Central	Doctor		Male	32	35YR/MALE ON IV LIGNOCAINE & ADRENALINE. GOT SWOLLEN & WAS FAILING TO BREATH-SEVERE
498	1004	Private For-Profit	Private Hospital	KAMPALA	Central	Nurse	Enrolled Nurse	Female	29	LADY IN LATE TWENTIES GIVEN IV HYDROCORTISONE,GOT SEVERE BURNING SENSATION IN PRIVATE PARTS IMMEDIATELY AFTER ADMINISTRATION OF THE DRUG
1056	1087	Public	District Hospital	TORORO	Eastern	Nurse	Registered Mental Health	Female	32	18YR/MALE MAN TAKING ORAL PHENORBABITONE GOT RASHES ALL OVER THE BODY SEVERE
	13	Public	National Referral	KAMPALA	Central	Doctor		Female	25	SJS SECONDARY TO CARBAMAZEPINE
508	481	Public	Regional Referral	LIRA	Other/NK	Doctor		Female	35	22YR/FEMALE ON INJECTAPLAN CAME BACK AFTER 1 WEEK WITH CONSTANT BLEEDING. DISCONTINUED USE OF THE INJECTAPLAN
167	61	Private For-Profit	Private Hospital	KAMPALA	Central	Doctor		Male	30	30YR PRIME GRAVID GIVEN MAGNESIUM SULPHATE TO PREVENT FITS-COMPLAINED OF EXCESSIVE HEAT & STARTED SWEATING WITH CHANGE IN HAEMODYNAMIC STABILITY
164	1878	Public	National Referral	KAMPALA	Central	Pharm		Female	36	SEDATION IN 12YR OLD PATIENT GIVEN ORAL CHLORPHENIRAMINE FOR ALLERGIC REACTION-MILD
892	1184	Private For-Profit	Pharmacy	MBRA	Other/NK	Other		Male		24YR/FEMALE DEVELOPED AN INFLAMATORY ITCHY RASH ALL-OVER AFTER TAKING ORAL PIPERAZINE,STOPPD IT AND PATIENT IMPROVED - MODERATE
	1606	Public	National Referral	KAMPALA	Central	Doctor		Male	35	VOMITING FOLLOWING THE ZINC ADMINISTRATION
	1248	Public	National Referral	KAMPALA	Central	Nurse	Registered Nurse	Female	25	SEVERE MUCOSITIS IN PATIENTS WHO GOT 5-FLUOROURACIL (ANTICANCER AGENT)
704	602	Private For-Profit	Pharmacy	KAMULI	Eastern	Nurse	Enrolled Nurse	Male	32	24YR OLD BOY TOOK OVERDOSE OF CHLORPHENIRAMINE & FELL ASLEEP FOR A FULL DAY.

556	774	Private For-Profit	Other	MASINDI	Other/NK	Other	Nursing Assistant	Male	24	EXCESSIVE VAGINAL BLEEDING IN 26YR OLD PATIENT GIVEN SUBCUTANEOUS NORPLANTS
870	768	Private For-Profit	Health Centre IV	JINJA	Eastern	Nurse	Nursing Assistant	Female	28	WOMEN REACTING TO INJECTAPLAN AND PILLPLAN WITH EXCESSIVE BLEEDING
1007	1038	Private Not-for-Profit	Private Hospital	TORORO	Eastern	Nurse	Enrolled Mental Health N	Female	20	27YR/MALE WITH PROTRUDING TONGUE AFTER ORAL CHLORPROMAZINE - MILD
	1246	Public	National Referral	KAMPALA	Central	Nurse	Registered Nurse Midwife	Female	45	PATIENT AFTER CHEMOTHERAPHY GOT SEVERE SKIN REACTION AFTER 4WEEKS OF TREATMENT. SITE WAS LEFT HARD
591	809	Private For-Profit	Health Centre IV	MASINDI	Other/NK	Doctor		Male	36	FEMALE 25YRS WITH IMPLANT,EXCESSIVE UTERINE BLEEDING, MODERATE
	1223	Public	National Referral	KAMPALA	Central	Nurse	Registered Nurse Midwife	Female	45	PATIENT WITH BURNT LEG WHEN PUT ON CHEMOTHERAPHY,HE GOT PAIN & LEG HAD WOUNDS
714	612	Private For-Profit	Health Centre IV	KAMULI	Eastern	Nurse	Enrolled Nurse	Female	22	WOMAN CAME FEELING DIZZY AFTER TAKING FLUFED (TRIPROLIDINE, PARACETAMOL & PSEUDOEPHEDRINE),TOLD HER TO TAKE WATER & REST
58	911	Public	National Referral	KAMPALA	Central	Doctor		Male	29	50YR/FEMALE ON ORAL LISINOPRIL FOR HYPERTENSION WITH PERSISTENT COUGH,MODERATE
89	1628	Public	National Referral	KAMPALA	Central	Doctor		Female	43	14YR OLD GIRL ORALLY ADMINISTERD HALOPERIDOL -GOT EXTRAPYRAMIDAL SIDE EFFECTS, MODERATE. I REDUCED DOSE AND GAVE BENZHEXOL
		**No Medication Reported**								
	406	Public	National Referral	KAMPALA	Central	Doctor		Male	26	SEVERE PRURITUS
459	457	Private For-Profit	Health Centre III	GULU	Other/NK	Nurse	Registered Nurse	Female	24	IT WAS MILD
228	972	Public	National Referral	KAMPALA	Central	Doctor		Male	38	80YR/FEMALE WITH GENERALIZED SKIN ERUPTIONS & WOUNDS, NOT SURE OF DRUG BUT IT WAS SEVERE. ROUTE - ORAL.
	1203	Public	National Referral	KAMPALA	Central	Doctor		Female	26	SULPHUR-ASSOCIATED SKIN BULLOUS ERUPTIONS (FDR KIND)
	1213	Public	National Referral	KAMPALA	Central	Doctor		Male	28	NAUSEA,VOMITING
	401	Public	National Referral	KAMPALA	Central	Pharm		Male		DIZZINESS,LIGHT HEADEDNESS,NAUSEA
	830	Public	National Referral	KAMPALA	Central	Doctor		Female	39	MUSCLE RIGIDITY AND EXCESSIVE SALIVATION
20	839	Public	National Referral	KAMPALA	Central	Other		Male	56	EXTRAPYRAMIDAL SIDE EFFECTS E.G. TREMORS,TARDIVE DYSKINESIA, AKATHISIA ETC
	838	Public	National Referral	KAMPALA	Central	Doctor		Male	46	SJS TO ART,HEPATOTOXICITY IN TB PATIENT
	2017	Private For-Profit	Health Centre III	WAKISO	Central	Nurse	Registered Midwife	Female	40	VERY WEAK,SORES IN THE MOUTH,SKIN RASH
	1222	Public	National Referral	KAMPALA	Central	Nurse	Registered Nurse	Male		OTOTOXITY
	1243	Public	National Referral	KAMPALA	Central	Nurse	Registered Mental Health	Female	40	EXTENSIVE ORAL SORES WITH SEVERE DIARRHOEA &GENERALISED BODY RASH
	844	Public	National Referral	KAMPALA	Central	Doctor		Male	26	SJS
	827	Private For-Profit	Health Centre IV	KAMPALA	Central	Doctor		Male	40	ABDOMINAL DISCOMFORT,GENERAL MALAISE
45	1256	Public	National Referral	KAMPALA	Central	Nurse	Registered Nurse	Female	25	EXTRAVASATION
247	1259	Public	National Referral	KAMPALA	Central	Doctor		Male		25YR WITH SEVERE ADR THAT THE SKIN & MUCOUS MEMBRANES WITH REDDENING OF EYES
499	1005	Private For-Profit	Private Hospital	KAMPALA	Central	Nurse	Registered Nurse	Female	26	30YR,WHOLE BODY ITCHING ROUTE OF ADMINISTRATION WAS INTRAVENOUS. REACTION MODERATE
	839	Private For-Profit	Other	KAMPALA	Central	Pharm		Male	42	HYPERSENSITIVITY TO TREATMENT PRESENTING WITH SKIN RASHES AND BODY ITCHES
	2021	Private For-Profit	District Hospital	WAKISO	Central	Doctor		Male	40	VOMITING,RIGORS,SKIN RASH
	1228	Public	National Referral	KAMPALA	Central	Other	Registered Comprehensiv	Female	34	ROLLING EYES,BODY TREMORS, SALIVA THROUGH THE MOUTH,TONGUE OUT,BODY WEAKNESS
	842	Public	National Referral	KAMPALA	Central	Nurse	Other	Female	25	SKIN RASH AND ITCHING
	611	Public	National Referral	KAMPALA	Central	Nurse	Registered Nurse Midwife	Female	26	RASH, STIFFNESS

679	577	Private For-Profit	Drug Shop	KAMULI	Eastern	Nurse	Nursing Assistant	Female	29	ANAPHYLAXIS,MENTAL EFFECT,ALLERGIC REACTION,HEADACHE,ABDOMINAL DISCOMFORT
	405	Public	National Referral	KAMPALA	Central	Doctor		Male	31	PATIENT DEVELOPED WHEALS ALL OVER THE BODY
437	433	Private For-Profit	Pharmacy	GULU	Other/NK	Nurse	Enrolled Comprehensive	Female	26	MODERATE
	6	Public	National Referral	KAMPALA	Central	Pharm		Female	24	BLOOD IN URINE
174	68	Private Not-for-Profit	Private Hospital	KAMPALA	Central	Nurse	Enrolled Midwife	Female	28	PATIENT WAS COMPLAINING OF SEVERE HEADACHE & I REFERED HIM TO A MEDICAL OFFICER
	3	Private Not-for-Profit	Private Hospital	KAMPALA	Central	Other		Male	30	SKIN RASH
933	1225	Public	Health Centre III	MBRA	Other/NK	Other		Male	47	BODY SWELLING WITH ANEMIA
504	1010	Private Not-for-Profit	Private Hospital	WAKISO	Central	Other		Male		GENERALISED BODY RASH WITH SORES IN 30YR OLD MALE. REACTION WAS SEVERE
	1632	Private For-Profit	Private Hospital	KAMPALA	Central	Doctor		Male	27	SJS
	602	Public	National Referral	KAMPALA	Central	Nurse	Registered Nurse	Female	36	SKIN PEELING WHICH COULD LEAVE SCARS
622	520	Public	District Hospital	KAMULI	Eastern	Nurse	Enrolled Nurse	Female	39	1YR OLD CHILD DEVELOPED ABSCESSES & WE USED DRAINAGE AS FORM OF TREATMENT
	1218	Public	National Referral	KAMPALA	Central	Nurse	Registered Nurse	Female	26	SEVERE NEUTROPENIA WITH VERY HIGH UNCONTROLLED TEMPERATURE, SEVERE MUCOSITIS, EXCESSIVE VOMITING, EXCESSIVE WEIGHT LOSS & MALNUTRITION,TUMOR LYSIS SYNDROME
	1229	Public	National Referral	KAMPALA	Central	Other	Other	Male	32	TREMORS OF THE HANDS & ENTIRE BODY,COMING OUT OF TONGUE,ROLLING EYES,BODY WEAKNESS
	816	Public	National Referral	KAMPALA	Central	Doctor		Male	26	SJS
	1247	Public	National Referral	KAMPALA	Central	Nurse	Registered Nurse	Female	27	SJS, MUCOSITIS (WHERE PATIENT CANNOT PUT ANYTHING IN THE MOUTH), DROP IN BLOOD COUNTS
	2010	Private Not-for-Profit	Regional Referral	WAKISO	Central	Doctor		Male	40	DIARRHOREA,CONSTIPATION

KEY

ADR = ADVERSE DRUG REACTION
ART = ANTIRETROVIRAL THERAPY
AZT = ZIDOVUDINE
HAART = HIGHLY ACTIVE ANTIRETROVIRAL THERAPY
FLAGYL = METRONIDAZOLE
EFV = EFAVIRENZ
3TC = LAMIVUDINE
ISS = IMMUNOSUPRESSED SYNDROME

IV = INTRAVENOUS
NVP = NEVIRAPINE
MEPHAQUINE = MEFLOQUINE
QNN = QUININE
SEPTRIN = COTRIMOXAZOLE
SJS = STEVENS-JOHNSON SYNDROME
TDF = TENOFOVIR
* = CHECK APPENDIX - 2 OR MORE ADVERSE REACTIONS DESCRIBED
