# Supplementary material for: Rare, Serious, and Comprehensively Described Suspected Adverse Drug Reactions Reported by Surveyed Healthcare Professionals in Uganda
Source: PLoS One. 2015 Apr 23;10(4):e0123974. doi: 10.1371/journal.pone.0123974 (PMC4408100; doi:10.1371/journal.pone.0123974)
Supplement: S5 Appendix — (PDF) [file pone.0123974.s005.pdf]

	Appendix S5: Comprehensiveness of 241 ADR descriptions from 268 healthcare professionals who suspected ADRs in the previous one month											
file	id	Description	Body Site						Drugclass	Route	Severity	Patient's age
			GI	Skin	Swellg	Bleeding	CNS	Other				
5	34	VOMITING & IRRITABILITY AFTER IV QUININE ADMINISTRATION BUT LATER STABILISED. REACTIONS WERE MILD.	1				1		Antimal only	Injectable	Mild	
20	839	EXTRAPYRAMIDAL SIDE EFFECTS E.G. TREMORS,TARDIVE DYSKINESIA, AKATHISIA ETC					1				Unmentioned	
26	1810	TINNITUS IN A 27YR-OLD AFTER IV QUININE. SEVERE (LED TO ALTERED CONSCIOUSNESS).					1		Antimal only	Injectable	Severe	27
39	1625	PATIENT ON POST-EXPOSURE-PROPHYLAXIS (CBV/EFV) PRESENTS WITH EUPHORIA & DECREASED SLEEP: EFV-INDUCED NEUROPSYCHIATRIC MOOD					1		ART only		Unmentioned	
45	1256	EXTRAVASATION						1			Unmentioned	
49	902	SEVERE HYPOGLYCEMIA IN ADULT-DRUG WAS ARTESUNATE/AMODIAQUINE						1	Antimal only		Severe	
53	906	REDUCED BLOOD SUGAR & LOW PULSE AFTER TAKING CHLORAMPHENICOL INJECTION						1	Antibact only	Injectable	Unmentioned	
58	911	50YR/FEMALE ON ORAL LISINOPRIL FOR HYPERTENSION WITH PERSISTENT COUGH,MODERATE					1		Other	Oral&Topical	Moderate	50
59	912	VOMITING,IV CEFTRIAXONE-MODERATE	1						Antibact only	Injectable	Moderate	
61	914	REACTION TO SEPTRIN PROPHYLAXIS IN A NEWLY DIAGNOSED HIV PATIENT STARTING SEPTRIN DOSE							Antibact only		Unmentioned	
63	916	NVP SKIN HYPERSENSITIVITY		1					ART only		Unmentioned	
69	922	30YR PATIENT REACTED TO ART ADMINISTERED ORALLY. PATIENT LOST SKIN & MUCUS MEMBRANES-SEVERE		1				1	ART only	Oral&Topical	Severe	30
74	42	30YR HIV+ MALE REACTED MODERATELY TO ORAL SEPTRIN PROPHYLAXIS							Antibact only	Oral&Topical	Moderate	30
76	48	40YR MALE REACTED TO PENICILINS (FLUCLOXACILLIN-AMOXICILLIN COMBINATION) - GOT RASHES AFTER FIRST DOSE, REACTION WAS MODERATE		1					Antibact only		Moderate	40
82	54	MILD SULPHUR REACTION TO ORAL TREATMENT WITH SULFADOXINE-PYRIMETHAMINE (SP) AGE-ADULT							Antimal only	Oral&Topical	Mild	
87	1626	1YR OLD GOT MILD REACTION TO COTRIMOXAZOLE FOR URTI							Antibact only		Mild	1
89	1628	14YR OLD GIRL ORALLY ADMINISTERD HALOPERIDOL -GOT EXTRAPYRAMIDAL SIDE EFFECTS, MODERATE. I REDUCED DOSE AND GAVE BENZHEXOL					1		Other	Oral&Topical	Moderate	14
94	923	2.5YR OLD BOY REACTED TO SEPTRIN							Antibact only		Unmentioned	2.5
104	983	ALCOHOL 95% ADMINISTERED IN THE EYE OF A PATIENT						1	Other		Unmentioned	
110	1252	PROLONGED HEAVY FLOW,INCREASED BLOOD PRESSURE & PAIN OF ARM WHILE USING IMPLANTS				1		1	Other		Unmentioned	
123	1839	ORAL NEVIRAPINE INDUCED RASH-SJS IN 26YR PATIENT. [DESELECTED - REACTION TO EFAVIRENZ CAUSING AUDITORY & VISUAL HALLUCINATIONS]		1					ART only	Oral&Topical	Unmentioned	26
152	1866	SWELLING OF FACE IN 16YR PATIENT AFTER SWALLOWING COARTEM (ORALLY)-MODERATE			1				Antimal only	Oral&Topical	Moderate	16
154	1868	9YR OLD MALE GOT SKIN RASH AFTER TAKING ORAL PENICILLIN.ONSET WAS SUDDEN BUT RESOLVED AFTER USE OF TOPICAL HYDROCORTISONE CREAM		1					Antibact only	Oral&Topical	Unmentioned	9
160	1874	19YR FEMALE PATIENT WITH MALARIA & COUGH REACTED SEVERELY TO COMBINATION OF QNN-IV & ORAL SEPTRIN. MANAGED WITH PARENTERAL HYDROCORTISONE							Antibact & Antimal	Injectable	Severe	19

163	1877	28YR FEMALE REACTED TO ORACURE GEL (LIGNOCAINE & CETYLPYRIDINIUM) FOR ORAL SORES-GOT SWOLLEN LIPS/TONGUE			1				Antiseptic & Anaesthe	Oral&Topical	Unmentioned	28
164	1878	SEDATION IN 12YR OLD PATIENT GIVEN ORAL CHLORPHENIRAMINE FOR ALLERGIC REACTION-MILD					1		Other	Oral&Topical	Mild	12
165	982	BODY ITCHING,SWELLING &DISCOMFORT DUE TO CEPHALEXIN CAPS		1	1			1	Antibact only	Oral&Topical	Unmentioned	
167	61	30YR PRIME GRAVID GIVEN MAGNESIUM SULPHATE TO PREVENT FITS-COMPLAINED OF EXCESSIVE HEAT & STARTED SWEATING WITH CHANGE IN HAEMODYNAMIC STABILITY		1			1		Other		Unmentioned	30
170	64	25YR-OLD ANAEMIC, AZT						1	ART only		Unmentioned	25
171	65	28YR FEMALE PATIENT GIVEN IM MORPHINE, GOT GENERALIZED SKIN ITCHING OF MODERATE SEVERITY		1					Analgesic only	Injectable	Moderate	28
174	68	PATIENT WAS COMPLAINING OF SEVERE HEADACHE & I REFERED HIM TO A MEDICAL OFFICER					1				Severe	
175	69	IV PETHIDINE GIVEN TWICE CAUSED A SKIN REACTION		1					Analgesic only	Injectable	Unmentioned	
182	1263	24 YR PATIENT, VOMITING AFTER ORAL DOXYCYCLINE, MODERATE	1						Antibact only	Oral&Topical	Moderate	24
183	818	50YR FEMALE PATIENT GOT NEVIRAPINE (ORAL) HYPERSENSITIVITY INVOLVING ALL MUCUS MEMBRANES,SEVERE BUT RESOLVED						1	ART only	Oral&Topical	Severe	50
185	2006	5YR OLD CHILD ON ORAL AMPICLOX (AMPICILLIN & CLOXACILLIN) DEVELOPED GENERALIZED RASHES. IT WAS MODERATE		1					Antibact only	Oral&Topical	Moderate	5
187	2008	28YR FEMALE ON ORAL DUOVIR-N (AZT/3TC/NVP), GOT RASH WHICH WORSENED & SHE DEVELOPED SJS		1					ART only	Oral&Topical	Unmentioned	28
191	2004	36YR MALE ADMITTED WITH SJS FOLLOWING INITIATION OF ORAL NEVIRAPINE,SEVERE EVENT. [DESELECTED - 17YR OLD FEMALE ON ORAL AZITHROMYCIN GOT SKIN RASHES NECESSITATING ADMISSION-MODERATE]		1					ART only	Oral&Topical	Severe	36
192	2005	SKIN RASH IN A 45YR FEMALE PATIENT ON CBV/EFV/CTX FOR 2YRS TAKEN ORALLY. MODERATE. WAS REFFERED TO DERMATOLOGIST		1					ART only	Oral&Topical	Moderate	45
194	984	37YR FEMALE PATIENT ON ORAL ARVS/TB GOT ENTIRE BODY RASH		1					ART & AntiTBs	Oral&Topical	Unmentioned	37
197	941	24YR/FEMALE KNOWN IMMUNOSUPPRESSED SYNDROME (ISS) PATIENT ON ANTI-TBS WHO REACTED TO COTRIMOXAZOLE - SJS & ALSO HAD TOXOPLASMOSIS		1					Antibact & AntiTBs		Unmentioned	24
205	949	26YR ANAEMIC FEMALE WITH SEVERE PRE-ECLAMPSIA FINALLY DELIVERED & WAS MANAGED POST-OPERATIVELY WITH GENTAMICIN-SUSTAINED ACUTE RENAL FAILURE WITH ANAEMIA FOR 5 DAYS & OTHER COMPLICATIONS						1	Antibact only		Unmentioned	26
227	842	30YR/FEMALE PATIENT GIVEN IM MORPHINE & IV CEFTRIAXONE AFTER CAESARIAN-SECTION. GOT GENERALIZED BODY RASH & ITCHING 15MIN LATER		1					Antibact & Analgesic	Injectable	Unmentioned	30
228	972	80YR/FEMALE WITH GENERALIZED SKIN ERUPTIONS & WOUNDS, NOT SURE OF DRUG BUT IT WAS SEVERE. ROUTE - ORAL.		1						Oral&Topical	Severe	80
234	978	35YR/MALE ON IV LIGNOCAINE & ADRENALINE. GOT SWOLLEN & WAS FAILING TO BREATH-SEVERE			1		1		Other	Injectable	Severe	35
243	1254	42YR/FEMALE SERO-POSITIVE, NAIVE TO HAART WITH MILD ITCHY RASH, APPETITE LOSS ,RISE IN BODY TEMPERATURE & ITCHING WHICH INTENSIFIED ON SWALLOWING SEPTRIN TABLETS FIVE WEEKS AGO. PATIENT RETURNED TO FACILITY & LAB INVESTIGATIONS SHOWED DERRANGED LIVER FUNCTION TEST VARIABLES	1	1				1	Antibact only	Oral&Topical	Unmentioned	42
247	1259	25YR WITH SEVERE ADR THAT THE SKIN & MUCOUS MEMBRANES WITH REDDENING OF EYES		1				1			Severe	25

253	994	ADULT SEVERELY REACTED TO SEPTRIN AND TB DRUGS TAKEN ORALLY							Antibact & AntiTBs	Oral&Topical	Severe	
259	987	28YR/FEMALE GOT BODY ITCHING AFTER TAKING ORAL QUININE. ADR WAS MODERATE		1					Antimal only	Oral&Topical	Moderate	38
274	2025	2YR OLD, CEFTRIAXONE, INTRAVENOUS, MILD							Antibact only	Injectable	Mild	2
278	2029	ORAL DICLOFENAC 50MG, HAEMOPTYSIS AFTER 2 DAYS - WAS SEVERE. [DESELECTED - DUOCOTEXCIN - VOMITING UNNECESSARILY]				1			Analgesic only	Oral&Topical	Severe	
279	80	LOOSE STOOL,BODY ITCHING & URTICARIA WITH BLISTERING FOLLOWING USE OF PENICILINS	1	1					Antibact only		Unmentioned	
301	1650	ELDERLY PATIENT ON PIROXICAM COMPLAINED OF HEART PAIN WHICH INDICATED PUD & ALSO GOT OEDEMA DUE TO PENICILLINS	1		1				Antibact & Analgesic		Unmentioned	
304	1653	28YRS, COTRIMOXAZOLE, SEVERE							Antibact only		Severe	28
313	1662	MILD ADR.ELDERLY MALE PATIENT GOT ITCHING AFTER BENZYL PENICILIN BY IV ROUTE		1					Antibact only	Injectable	Mild	
325	2034	18YR/FEMALE REACTED TO INTRAVENOUS CEFTRIAXONE BY SWELLING OF HAND THROUGH WHICH DRUG WAS GIVEN & SEVERE BACK PAIN-MILD			1			1	Antibact only	Injectable	Severe	18
326	2035	ORAL ADMINISTRATION OF NEVIRAPINE IN PATIENT LEADING TO HEPATOTOXITY						1	ART only	Oral&Topical	Unmentioned	
327	843	35YR/FEMALE HYPERSENSITIVITY TO PENICILLINS							Antibact only		Unmentioned	33
329	845	30YR/MALE ON ORAL TINIDAZOLE GOT HEADACHE & DIZZINESS.IT WAS MILD					1		Antibact only	Oral&Topical	Mild	30
331	847	24YR PATIENT REACTED TO PENICILLIN ORAL ROUTE,GOT GENERALIZED RASH AND BODY ITCHING		1					Antibact only	Oral&Topical	Unmentioned	24
332	1672	ORAL ROUTE OF TRAMADOL RESULTED INTO SEVERE ITCHY SORES ALL-OVER THE BODY THAT PERSISTED		1					Analgesic only	Oral&Topical	Severe	
343	1687	26YR OLD REACTED TO SEPTRIN. MODERATE REACTION							Antibact only		Moderate	26
349	86	58YR OLD ON ORAL COTRIMOXAZOLE GOT SEVERE URTICARIA AND SKIN RASHES ALL OVER THE BODY		1					Antibact only	Oral&Topical	Severe	58
359	97	PATIENT 52YR B/S +VE FOR MALARIA PARASITES. GIVEN COARTEM FOR THREE DAYS' TREATMENT, AFTER 1 DAY GOT SKIN ITCHING & SEVERE RASH.STOPPED DRUG, GAVE IV QNN & PATIENT IMPROVED		1					Antimal only		Severe	52
365	103	20YR/FEMALE ON PENICILIN GOT MILD REACTION							Antibact only		Mild	20
370	109	MILD ADR.PATIENT ON ORAL EFV HAD GYNAECOMASTIA AND JERKS					1	1	ART only	Oral&Topical	Mild	
390	848	MILD DYSPEPSIA FROM ADULTS USING ORAL ANALGESICS FOR ATHRITIS	1						Analgesic only	Oral&Topical	Mild	
396	854	REACTION TO CEFTRIAXONE WITH MILD INFLAMATION &SWELLING AT POINT OF INJECTION. [DESELECTED - REACTION TO CIPROFLOACIN WITH SWELLING OF TONGUE].			1				Antibact only	Injectable	Mild	
398	856	PATIENT PUT ON IV ARTHEMETHER,GOT GENERALIZED ITCHY SKIN RASH ABOUT 1HOUR AFTER INJECTION. WAS MILD		1					Antimal only	Injectable	Mild	
402	1273	30YR/FEMALE ON AZT/3TC/NVP GOT BODY RASH &ITCHING AFTER 2WEEKS.MODERATE ADR		1					ART only		Moderate	30
403	1287	PERIPHERAL NEUROPATHY DUE TO AZT						1	ART only		Unmentioned	
409	1368	35YR/FEMALE, WITH CD4 100 CELLS/DL ON AZT/3TC/NVP,AFTER 2WEEKS SHE CAME BACK WITH A SEVERE GENERALIZED BODY RASH		1					ART only		Severe	35
411	1371	28YR OLD ON COTRIMOXAZOLE TAB TAKEN ORALLY,MODERATE ADR (HYPERSENSITIVITY)							Antibact only	Oral&Topical	Moderate	28

417	1692	ADULT WITH UTI GIVEN ORAL CIPROFLOXIN GOT ARTHRALGIA, MODERATE ABDOMINAL PAIN,VOMITING & RESTLESSNESS. SEVERE ADR.	1				1	1	Antibact only	Oral&Topical	Severe	
423	419	22YR/FEMALE LADY NURSE WHO GOT NEEDLE STICK INJURY & WAS INITIATED ON PEP WITH AZT/3TC-12 DAYS LATER GOT SEVERE RASH (SJS) & WAS ADMITED AND TREATED		1					ART only		Severe	22
429	425	13YR/MALE ON PREDNISOLONE ORAL ROUTE GOT SEVERE EPIGASTRIC PAIN WHICH WAS MANAGED AS SEVERE STEROID-INDUCED GASTRITIS	1						Other	Oral&Topical	Severe	13
430	426	27YR/FEMALE ON PEP ORAL ROUTE GOT BLISTERS 3 DAYS LATER WITH BURNING SENSATION ALL-OVER THE BODY.MODERATE		1			1		ART only	Oral&Topical	Moderate	27
437	433	MODERATE									Moderate	
450	447	ADULT FEMALE ON ORAL COTRIMOXAZOLE GOT SWELLING AND ITCHY FACE.MILD		1	1				Antibact only	Oral&Topical	Mild	
453	450	41YR/MALE GIVEN PANADOL (PARACETAMOL), STARTED SHAKING & SWEATING 10 MINUTES LATER.WAS PUT ON A DRIP AND HE BECAME FINE.		1			1		Analgesic only		Unmentioned	41
459	457	IT WAS MILD									Mild	
465	463	72YR/FEMALE REACTED TO BENZYL PENICILLIN WITH SHIVERS,ITCHY BODY RASHES & PROFUSE SWEATING IMMEDIATELY AFTER INJECTION - MODERATE		1			1		Antibact only	Injectable	Moderate	72
466	464	32YR/FEMALE KNOWN ISS PATIENT ON COTRIMOXAZOLE CAME WITH SEVERE BODY RASHES & SLOUGHING. [DESELECTED - 18YR/MALE GIVEN IV FORTIFIED PROCAINE PENICILLIN (PPF) & SHE COLLAPSED INSTANTLY WITH DIFFICULTY IN BREATHING & BODY TREMBLING]		1					Antibact only		Severe	32
471	469	3OYR ON AZT ORAL ROUTE GOT BODY RASH,FEVER, & ITCHING- MODERATE		1			1		ART only	Oral&Topical	Moderate	30
484	1314	ADULT PATIENT HAD ABDOMINAL CRAMPS & URTICARIA RASH DUE TO IV VANCOMYCIN - MODERATE	1	1					Antibact only	Injectable	Moderate	
486	1289	4YR OLD,EFAVIRENZ-ORAL ROUTE-MODERATE ADR							ART only	Oral&Topical	Moderate	4
495	863	38YR OLD FEMALE WITH SEVERE NEVIRAPINE HYPERSENSITIVITY							ART only		Severe	38
498	1004	LADY IN LATE TWENTIES GIVEN IV HYDROCORTISONE,GOT SEVERE BURNING SENSATION IN PRIVATE PARTS IMMEDIATELY AFTER ADMINISTRATION OF THE DRUG						1	Other	Injectable	Severe	29
499	1005	30YR,WHOLE BODY ITCHING ROUTE OF ADMINISTRATION WAS INTRAVENOUS. REACTION MODERATE		1						Injectable	Moderate	30
502	1008	40YR OLD ON ETHAMBUTOL ORAL ROUTE GOT BURNING SENSATION OF BOTH LIMBS & LOSS OF SIGHT - MODERATE SEVERITY					1	1	AntiTBs only	Oral&Topical	Moderate	40
504	1010	GENERALISED BODY RASH WITH SORES IN 30YR OLD MALE. REACTION WAS SEVERE		1							Severe	35
506	1013	26YR OLD ON ARVS (AZT+3TC-NVP) MODERATE ITCHY SKIN RASH-RESOLVED AFTER DISCONTINUATION OF ART. [DESELECTED -16YR OLD WITH URTICARIA DUE TO AMOXICILLIN - MILD ADR. WAS GIVEN HYDROCORTISONE].		1					ART only		Moderate	16
508	481	22YR/FEMALE ON INJECTAPLAN CAME BACK AFTER 1 WEEK WITH CONSTANT BLEEDING. DISCONTINUED USE OF THE INJECTAPLAN				1			Other	Injectable	Unmentioned	22
511	484	26YR/FEMALE HAD A HISTORY OF PEPTIC ULCER DISEASE - DUE TO INDOMETHACIN BUT WAS NOT SEVERE	1						Analgesic only		Moderate	26
518	491	10YR/MALE REACTED TO PANADOL (PARACETAMOL) WHEN TOOTH WAS REMOVED,BECAME DIZZY-MINOR					1		Analgesic only		Mild	10
522	495	26YR/MALE ON COARTEM ORAL ROUTE,RASH ON BOTH HANDS, ITCHING & SWELLING		1	1				Antimal only	Oral&Topical	Unmentioned	26

523	496	80YR OLD WOMAN REACTED TO COTRIMOXAZOLE.SKIN CHANGED BUT WAS A MILD CASE		1					Antibact only		Mild	80
550	2041	62YR/FEMALE ON COTRIMOXAZOLE ORAL ROUTE WITHIN TWO DAYS GOT MULTIPLE SKIN PATCHES, DEVELOPED SORES ON MUCOUS MEMBRANES WITH HIGH TEMPERATURE. GIVEN STEROIDS & SHE RECOVERED		1			1	1	Antibact only	Oral&Topical	Unmentioned	62
556	774	EXCESSIVE VAGINAL BLEEDING IN 26YR OLD PATIENT GIVEN SUBCUTANEOUS NORPLANTS				1			Other	Injectable	Unmentioned	26
563	781	27YR OLD WITH 237 CD4 CELLS/ML ON NEVIRAPINE ORAL ROUTE GOT SEVERE RASH		1					ART only	Oral&Topical	Severe	27
567	785	4YR OLD ON COARTEM ORAL ROUTE. MODERATE REACTION							Antimal only	Oral&Topical	Moderate	4
570	788	62YR/FEMALE ON ORAL MEPHAQUINE GOT SEVERE HEADACHE WITH MENTAL CONFUSION & INSOMNIA					1		Antimal only	Oral&Topical	Severe	62
574	792	25YR OLD ON IV TRAMADOL 100MG, DEVELOPED PALPITATIONS & SWEATING BUT GOT BETTER AFTER 20 MINUTES - MILD		1			1		Analgesic only	Injectable	Mild	25
587	805	SEPTRIN							Antibact only		Unmentioned	
591	809	FEMALE 25YRS WITH IMPLANT,EXCESSIVE UTERINE BLEEDING, MODERATE				1			Other		Moderate	25
597	815	ADULT FEMALE GOT EXTENSIVE SKIN RASH,WAS ON STARTER SEPTRIN PROPHYLAXIS		1					Antibact only		Unmentioned	
603	821	35YR ON AZT-CONTAINING REGIMEN ORAL ROUTE DEVELOPED SEVERE ANAEMIA LEADING TO ADMISSION & BLOOD TRANSFUSION						1	ART only	Oral&Topical	Severe	35
606	824	5YR OLD ON COTRIMOXAZOLE REACTED TO THE DRUG & WAS CHANGED TO CAP AMOXYCILLIN. MODERATE							Antibact only		Moderate	5
608	826	28YR/MALE - ALLERGIC REACTION TO ORAL CIPROFLOXACIN - SEVERE URTICARIA - SUBSTITUTED FOR CEPHALEXIN & CETIRIZINE. [DESELECTED - 30YR-OLD FEMALE REACTED TO NEVIRAPINE - MODERATE & WAS SUBSTITUTED FOR EFV]		1					Antibact only	Oral&Topical	Severe	28
612	830	45YR PATIENT ON ORAL TDF/3TC/NVP GOT SEVERE ABDOMINAL PAIN WHICH WAS IN COLICKY FORM	1						ART only	Oral&Topical	Severe	45
613	831	39YR/FEMALE ON ZIDOVIDINE ORAL ROUTE GOT ANAEMIA. SHE WAS BLOOD TRANSFUSED & AZT WAS REPLACED WITH TENOFOVIR						1	ART only	Oral&Topical	Unmentioned	39
614	832	40YRS,SEPTRIN,ORALLY.SJS		1					Antibact only	Oral&Topical	Unmentioned	40
622	520	1YR OLD CHILD DEVELOPED ABSCESSES & WE USED DRAINAGE AS FORM OF TREATMENT						1			Unmentioned	1
624	522	2YR OLD BOY WAS BROUGHT TO HOSPITAL WITH A REACTION TO QUININE & DOCTOR GAVE HIM AN ANTIDOTE BUT HE DIED							Antimal only		Unmentioned	2
626	524	42YR OLD WOMAN REACTED TO ARVS,TREATED WITH ANTIBIOTICS, THAT IS, CEFTRIAXONE							ART only		Unmentioned	42
628	526	37YR OLD GOT SJS AFTER TAKING ARVS. WAS LATER GIVEN IV FLUID ANTIBIOTICS, STEROID DRUGS & SUPPORTIVE TREATMENT		1					ART only		Unmentioned	37
679	577	ANAPHYLAXIS,MENTAL EFFECT,ALLERGIC REACTION,HEADACHE,ABDOMINAL DISCOMFORT	1				1				Unmentioned	
682	580	55YR OLD MALE ON QUININE TABS ORAL ROUTE GOT BODY ITCHING		1					Antimal only	Oral&Topical	Unmentioned	55
700	598	OLD WOMAN REACTED TO SEPTRIN,GENERALIZED BODY SORES,GAVE HER PREDISOLONE & PAIN KILLER PLUS BETADERM TOPICAL (BETAMETHASONE)		1					Antibact only		Unmentioned	
704	602	24YR OLD BOY TOOK OVERDOSE OF CHLORPHENIRAMINE & FELL ASLEEP FOR A FULL DAY.					1		Other		Unmentioned	24

705	603	CHILDREN REACTED TO CHLOROQUINE WITH BODY ITCHING,WERE GIVEN DEXAMETHASONE TO STOP THE REACTION		1					Antimal only		Unmentioned	
707	605	25YR ON QUININE ORAL ROUTE, MILD, ADVISED HIM TO DRINK A LOT OF FLUIDS & TO TAKE PAIN KILLERS							Antimal only	Oral&Topical	Mild	25
710	608	30 YR FEMALE REACTED TO INJECTAPLAN,OVER BLEEDING. I PRESCRIBED IBRUPROFEN & AMOXICILLIN FOR FOUR DAYS & BLEEDING STOPPED				1			Other	Injectable	Unmentioned	30
714	612	WOMAN CAME FEELING DIZZY AFTER TAKING FLUFED (TRIPROLIDINE, PARACETAMOL & PSEUDOEPHEDRINE),TOLD HER TO TAKE WATER & REST					1		Other		Unmentioned	
723	622	27YR/FEMALE ON ORAL DUOVIR-N (AZT/3TC/NVP) DEVELOPED SWELLING & ITCHING. WAS MODERATE		1	1				ART only	Oral&Topical	Moderate	27
724	623	PATIENT ON SEPTRIN ORAL ROUTE,DEVELOPED RASH ALL OVER BODY, TREATMENT WAS STOPPED		1					Antibact only	Oral&Topical	Unmentioned	
725	624	16YR OLD BOY TOOK OVERDOSE OF IBRUPROFEN (20 TABLETS).PUT HIM ON A DRIP & HE RECOVERED.							Analgesic only	Oral&Topical	Unmentioned	16
738	637	26YR/MALE HIV+ ON ORAL SEPTRIN,GOT BURNT FACE & LIPS-GIVEN ORAL DEXAMETHASONE FOR FIVE DAYS - SEVERE		1					Antibact only	Oral&Topical	Severe	26
750	649	25YR/MALE REACTED TO IV AMPICILLIN.MODERATE.WAS GIVEN HYDROCORTISONE IV							Antibact only	Injectable	Moderate	25
758	657	25YR OLD FEMALE ON CIPROFLOXACIN IV. GOT SJS.IT WAS SEVERE		1					Antibact only	Injectable	Severe	25
771	670	PATIENT REACTED TO QUININE WITH ITCHING, TREATED IT WITH CETIRIZINE		1					Antimal only		Unmentioned	
783	682	MAN REACTED TO FANSIDAR IV-VOMITING.TREATMENT WAS PLASIL AND WATER	1						Antimal only	Injectable	Unmentioned	
784	683	29YR/FEMALE REACTED TO SEPTRIN - RASHES, GAVE HER DEXAMETHASONE AND PANADOL TO TREAT THE RASHES		1					Antibact only		Unmentioned	29
786	685	ADR WAS DUE TO NEVIRAPINE WHILE PATIENT RECEIVED ART							ART only		Unmentioned	
791	690	21YR PATIENT REACTED TO PPF WITH VOMITING.TREATMENT CHANGED TO ERYTHOMYCIN 500MG	1						Antibact only		Unmentioned	21
799	698	52YR OLD FEMALE ON COARTEM ORAL ROUTE DEVELOPED SORES ON THE WHOLE BODY		1					Antimal only	Oral&Topical	Unmentioned	52
809	708	WOMAN ON SEPTRIN REACTED BY DEVELOPING GENERALIZED BODY RASH		1					Antibact only		Unmentioned	
851	749	28YR OLD FEMALE REACTED TO QUININE IV LEADING TO MISCARRIAGE				1			Antimal only	Injectable	Unmentioned	28
869	767	3YR OLD CHILD GIVEN IV QUININE & SHE BECAME HYPOGLYCAEMIC						1	Antimal only	Injectable	Unmentioned	3
870	768	WOMEN REACTING TO INJECTAPLAN AND PILLPLAN WITH EXCESSIVE BLEEDING				1			Other	Injectable	Unmentioned	
876	1168	COTRIMOXAZOLE ORAL, MILD ADR							Antibact only	Oral&Topical	Mild	
878	1170	23YR MALE TOOK IBUPROFEN GOT GENERALIZED SKIN RASH & ITCHING ALL-OVER THE BODY; IT WAS MODERATE. TREATED WITH IV HYDROCORTISONE		1					Analgesic only		Moderate	23
887	1179	35YRS ORAL EFV LED TO MODERATE MENTAL DISTURBANCE, SUBSTITUTN REDUCED IT					1		ART only	Oral&Topical	Moderate	35
888	1180	PATIENT ON ORAL QNN GOT SKIN RASH,TINNITUS,ABORTION,VERTIGO, NAUSEA, VOMITING, BLURRED VISION - SEVERE	1	1		1	1		Antimal only	Oral&Topical	Severe	
892	1184	24YR/FEMALE DEVELOPED AN INFLAMATORY ITCHY RASH ALL-OVER AFTER TAKING ORAL PIPERAZINE,STOPPD IT AND PATIENT IMPROVED - MODERATE		1					Other	Oral&Topical	Moderate	24
904	1196	29YR-OLD ON COTRIMOXAZOLE ORALLY, REACTION WAS MODERATE							Antibact only	Oral&Topical	Moderate	29
909	1201	60YR-OLD ON ORAL COTRIMOXAZOLE - RASHES,NAUSEA & SEVERE INVISIBLE SWEATS,MODERATE RXN	1	1			1		Antibact only	Oral&Topical	Moderate	60

910	1202	24 YR-OLD WITH SKIN PEELING OFF DUE TO NEVIRAPINE, REACTION WAS SEVERE		1					ART only		Severe	24
913	1205	74YR-OLD WITH CORNEAL ULCER NOT RESPONDING TO TOPICAL ANTIBIOTICS & ANTIVIRALS FOR 14 DAYS. STOPPED THE MEDICATION AND LEFT PATIENT ON ARTIFICIAL TEARS ONLY & NOTED GREAT IMPROVEMENT						1	Antibact & Antiviral	Oral&Topical	Unmentioned	74
914	1206	17YR-OLD ON IM QNN WITH POST INJECTION PARALYSIS, IT WAS SEVERE						1	Antimal only	Injectable	Severe	17
919	1211	32YR OLD HIV+ MALE STARTED ON COTRIMOXAZOLE ORALLY. IT WAS SEVERE.RECOVERED							Antibact only	Oral&Topical	Severe	32
927	1219	GASTROINTESTINAL DISTRESS DUE TO NSAID-INDUCED ULCERATION AFTER ORAL DICLOFENAC	1						Analgesic only	Oral&Topical	Unmentioned	
933	1225	BODY SWELLING WITH ANEMIA			1			1			Unmentioned	
949	134	WOMAN AGED 30YRS ON NEVIRAPINE & COTRIMOXAZOLE WHO DEVELOPED A SKIN RASH		1					Antibact & ART		Unmentioned	30
951	136	35 YR-OLD ISS PATIENT ON AZT/3TC/NVP GOT SKIN RASH MORE SEVERE ON LOWER LIMBS & SKIN STARTED PEELING OFF,DID NOT RESPOND TO STEROIDS OR ANTIFUNGALS		1					ART only		Severe	35
953	138	FEMALE 39YRS IV CEFTRIAXONE - MILD							Antibact only	Injectable	Mild	39
957	142	HIV+ PATIENT WITH BODY ACHES, SUDDEN SKIN RASH, BURNT SKIN, OOZING OF SWEAT-LIKE FLUID AFTER COTRIMOXAZOLE ADMINISTRATION - MILD SYMPTOMS		1				1	Antibact only		Mild	
986	1017	2YR OLD GIVEN IM QUININE DEVELOPED HYPOGLYCAEMIA - IMPROVED AFTER MANAGEMENT - SEVERE						1	Antimal only	Injectable	Severe	2
987	1019	38YR OLD IMMEDIATELY AFTER STARTING ORAL ARVS FELT LIKE HOT WATER HAD BEEN POURED ON HIM - SEVERE					1		ART only	Oral&Topical	Severe	38
988	1020	8YR OLD GIRL REFERRED FROM A CLINIC AFTER RECEIVING IV QUININE & SEPTRIN DEVELOPED BLISTERS ALL-OVER THE BODY & DIED ON ADMISSION - SEVERE		1					Antibact & Antimal	Injectable	Severe	8
990	1022	38YR/MALE ON SEPTRIN DEVELOPED SJS - MODERATE		1					Antibact only		Moderate	38
991	1023	A MAN 35YRS WITH GENERALIZED SKIN RASH AFTER TAKING ORAL SEPTRIN - SEVERE		1					Antibact only	Oral&Topical	Severe	35
992	1024	A CHILD OF 5YRS AFTER INTRAVENOUS QUININE. SHE DIED AFTER ARRIVAL							Antimal only	Injectable	Unmentioned	5
996	1027	5YR OLD GIRL FROM A CLINIC WHERE SHE WAS PUT ON IV QUININE & SEPTRIN. GOT BLISTERS ALL OVER THE BODY & DIED ON ADMISSION - SEVERE		1					Antibact & Antimal	Injectable	Severe	5
1002	1033	8YR OLD BOY DEVELOPED ITCHY BODY RASH AND SWOLLEN EYES AFTER RECEIVING BENZYL PENICILLIN - MODERATE. RESOLVED WITHIN 1HR AFTER GIVING HYDROCORTISONE		1	1				Antibact only		Moderate	8
1007	1038	27YR/MALE WITH PROTRUDING TONGUE AFTER ORAL CHLORPROMAZINE - MILD					1		Other	Oral&Topical	Mild	27
1008	1039	18YR OLD GIVEN IV CEFTRIAXONE. DRUG INJECTED VERY FAST & THE PATIENT DEVELOPED SEVERE VOMITING	1						Antibact only	Injectable	Severe	18
1009	1040	45YR/FEMALE DEVELOPED DEEP JAUNDICE (HEPATOTOXICITY) AFTER RECEIVING ORAL NEVIRAPINE. WAS GIVEN EFV AS ALTERNATIVE DRUG AND SHE IMPROVED						1	ART only	Oral&Topical	Unmentioned	45
1034	1065	MOTHER REPORTED CONVULSIONS WHENEVER THE CHILD TOOK HIS ORAL ARVS - MODERATE					1		ART only	Oral&Topical	Moderate	
1038	1069	65YR/FEMALE PATIENT'S BLOOD PRESSURE LOWERED FROM 105/60 TO 86/50 MMHG & STARTED SWEATING AT A TEMPERATURE OF 35.0 DEGREES CELSIUS DUE TO DYNAPAR (IV DICLOFENAC INFUSION) - MILD					1	1	Analgesic only	Injectable	Mild	65
1040	1071	A MAN DIAGNOSED WITH HIV TOOK COTRIMOXAZOLE AND GOT STEVENS-JOHNSON SYNDROME - SEVERE		1					Antibact only		Severe	

1043	1074	28YR OLD PATIENT WAS ADMINISTERED WITH COTRIMOXAZOLE TABLETS DEVELOPED BLACK PATCHES ON SKIN - MODERATE		1					Antibact only	Oral&Topical	Moderate	28
1047	1078	5YR OLD GIRL WAS REFERRED FROM A CLINIC AFTER TAKING IV QUININE & SEPTRIN WITH BLISTERS ALL-OVER THE BODY. SHE DIED ON ADMISSION - SEVERE		1					Antibact & Antimal	Injectable	Severe	5
1050	1081	3-MONTH-OLD BABY WAS INJECTED WITH DPT AND GOT A REDENNING OF INJECTION SITE		1					Other	Injectable	Unmentioned	0.25
1052	1083	36YR OLD ON ORAL FANSIDAR (SULPHADOXINE/PYRIMETHAMINE) DEVELOPED HYPERPIGMENTATION OF THE SKIN & THE THROAT - MILD		1					Antimal only	Oral&Topical	Mild	36
1056	1087	18YR/MALE MAN TAKING ORAL PHENORBABITONE GOT RASHES ALL OVER THE BODY SEVERE		1					Other	Oral&Topical	Severe	18
1057	1088	32YR/FEMALE ISS PATIENT ON AZT/3TC/NVP FOR 3 MONTHS GOT SKIN RASH ALL OVER THE BODY - STEVEN JOHNSON SYDROME - SEVERE		1					ART only		Severe	32
1058	1089	30YR OLD - SULPHUR REACTION-ORAL COTRIMOXAZOLE, SKIN RASH - MILD ; [DESELECTED - 40YR OLD NEVIRAPINE REACTION - SEVERE]		1					Antibact only	Oral&Topical	Mild	30
1061	1092	A 20YR OLD TAKING SEPTRIN ORALLY GOT SEVERE SKIN RASH ALL OVER THE BODY		1					Antibact only	Oral&Topical	Severe	20
1067	1098	4YR HIV+ CHILD ON PROTEASE INHIBITORS SWITCHED TO ORAL EFV - SEVERE BEHAVIORAL DISTURBANCE INCLUDING AGGRESSION, INSOMNIA & HYPERACTIVITY					1		ART only	Oral&Topical	Severe	4
1073	1104	A FOUR-AND-A-HALF-YEAR-OLD CHILD TOOK CHLOROQUINE & DEVELOPED SKIN RASHES - MODERATE		1					Antimal only		Moderate	4.5
1096	1116	IV/IM PENICILLINS - MODERATE RASHES AND ITCHING		1					Antibact only	Injectable	Moderate	
1109	1129	9-MONTH-OLD BABY ON ORAL COARTEM - VOMITING BUT RESOLVED. MILD REACTION	1						Antimal only	Oral&Topical	Mild	0.75
1157	882	32YR OLD REACTED TO ORAL NEVIRAPINE - SJS. REACTION WAS MODERATE		1					ART only	Oral&Topical	Moderate	32
1158	883	ORAL NEVIRAPINE, SEVERE HEADACHE, PATIENT STABILIZED					1		ART only	Oral&Topical	Severe	
1159	884	ORAL NEVIRAPINE, MODERATE REACTION, DRUG WITHHELD & PATIENT STABILIZED							ART only	Oral&Topical	Moderate	
1161	886	NVP RASHES, AZT ANEMIA, ATAZANAVIR HEPATOTOXICITY JAUNDICE		1				1	ART only		Unmentioned	
1162	887	40YR OLD SEVERE HEADACHE AFTER ORAL LOSARTAN					1		Other	Oral&Topical	Severe	40
1163	888	23YR OLD,FLAGYL,ITCHING & NAUSEA,MILD	1	1					Antibact only		Mild	23
1165	890	NVP RASH		1					ART only		Unmentioned	
	816	SJS		1							Unmentioned	
	1218	SEVERE NEUTROPENIA WITH VERY HIGH UNCONTROLLED TEMPERATURE, SEVERE MUCOSITIS, EXCESSIVE VOMITING, EXCESSIVE WEIGHT LOSS & MALNUTRITION,TUMOR LYSIS SYNDROME	1				1	1			Severe	
	2017	VERY WEAK,SORES IN THE MOUTH,SKIN RASH	1	1				1			Unmentioned	
	602	SKIN PEELING WHICH COULD LEAVE SCARS		1							Unmentioned	
	176	SKIN ITCHING SECONDARY TO OPIOID USE		1					Analgesic only		Unmentioned	
	405	PATIENT DEVELOPED WHEALS ALL OVER THE BODY		1							Unmentioned	
	401	DIZZINESS,LIGHT HEADEDNESS,NAUSEA	1				1				Unmentioned	
	2010	DIARRHOREA,CONSTIPATION	1								Unmentioned	
	1632	SJS		1							Unmentioned	
	402	SKIN RASH DUE TO NEVIRAPINE TABLETS		1					ART only	Oral&Topical	Unmentioned	
	825	PT ON ALUVIA (LOPINAVIR & RITONAVIR) COMPLAINED OF DRY MOUTH AND THROAT DISCOMFORT	1						ART only		Unmentioned	
	14	SKIN REACTIONS FOLLOWING IV CEFTRIAXONE		1					Antibact only	Injectable	Unmentioned	

	6	BLOOD IN URINE				1					Unmentioned	
	1222	OTOTOXITY					1				Unmentioned	
	16	PATIENT DEVELOPED PEPTIC ULCER DISEASE A FEW DAYS AFTER TAKING ACECLOFENAC	1						Analgesic only		Unmentioned	
	1203	SULPHUR-ASSOCIATED SKIN BULLOUS ERUPTIONS (FDR KIND)		1							Unmentioned	
	1611	GENERALIZED BODY RASH FOLLOWING USE OF ANTI-TBS		1					AntiTBs only		Unmentioned	
	1606	VOMITING FOLLOWING THE ZINC ADMINISTRATION	1						Other		Unmentioned	
	1229	TREMORS OF THE HANDS & ENTIRE BODY,COMING OUT OF TONGUE,ROLLING EYES,BODY WEAKNESS					1	1			Unmentioned	
	814	PATIENT GOT RASH AFTER TAKING SEPTRIN PROPHYLAXIS		1					Antibact only		Unmentioned	
	1248	SEVERE MUCOSITIS IN PATIENTS WHO GOT 5-FLUOROURACIL (ANTICANCER AGENT)	1						Other		Severe	
	2011	CHILD ON IV CIPROFLOXACIN GOT BODY ITCH, SWELLING AROUND THE FACE & AROUND SITE OF THE INJECTION		1	1				Antibact only	Injectable	Unmentioned	
	842	SKIN RASH AND ITCHING		1							Unmentioned	
	1225	HYPERSENSITIVITY REACTION TO SEPTRIN & BLOOD							Antibact & Blood		Unmentioned	
	833	SEVERE PALPITATIONS DUE TO OVER DOSE OF OMEPRAZOLE						1	Other		Severe	
	2021	VOMITING,RIGORS,SKIN RASH	1	1			1				Unmentioned	
	1223	PATIENT WITH BURNT LEG WHEN PUT ON CHEMOTHERAPHY,HE GOT PAIN & LEG HAD WOUNDS		1				1	Other		Unmentioned	
	808	MALE ADULT DEVELOPED ERECTILE DYSFUNCTION DUE TO NIFEDIPINE						1	Other		Unmentioned	
	1247	SJS, MUCOSITIS (WHERE PATIENT CANNOT PUT ANYTHING IN THE MOUTH), DROP IN BLOOD COUNTS	1	1				1			Unmentioned	
	183	PATIENT WAS ON CIPRO AND GOT SWOLLEN HANDS			1				Antibact only		Unmentioned	
	830	MUSCLE RIGIDITY AND EXCESSIVE SALIVATION	1				1				Unmentioned	
	838	[DESELECTED - SJS TO ART], HEPATOTOXICITY IN TB PATIENT						1			Unmentioned	
	611	RASH, STIFFNESS		1			1				Unmentioned	
	3	SKIN RASH		1							Unmentioned	
	13	SJS SECONDARY TO CARBAMAZEPINE		1					Other		Unmentioned	
	839	HYPERSENSITIVITY TO TREATMENT PRESENTING WITH SKIN RASHES AND BODY ITCHES		1							Unmentioned	
	1246	PATIENT AFTER CHEMOTHERAPHY GOT SEVERE SKIN REACTION AFTER 4WEEKS OF TREATMENT. SITE WAS LEFT HARD		1					Other		Severe	
	20	HIV+ PATIENT REACTED TO SEPTRIN PROPHYLAXIS AND GOT GENERALIZED SKIN RASH		1					Antibact only		Unmentioned	
	1228	ROLLING EYES,BODY TREMORS, SALIVA THROUGH THE MOUTH,TONGUE OUT,BODY WEAKNESS	1				1	1			Unmentioned	
	1601	DRUG REACTIONS TO ORAL SEPTRIN							Antibact only	Oral&Topical	Unmentioned	
	1243	EXTENSIVE ORAL SORES WITH SEVERE DIARRHOEA &GENERALISED BODY RASH	1	1							Severe	
	19	OVER DOSAGE OF NSAIDS AND PROTEASE INHIBITORS FROM PHYSCIANS WHO DONT CONSULT PHARMARCISTS							ART & Analgesic		Unmentioned	
	827	ABDOMINAL DISCOMFORT,GENERAL MALAISE	1					1			Unmentioned	
	2019	HIV+/FEMALE NEWLY ENROLLED ON HAART WITH NVP-BASED REGIMEN DEVELOPED SJS WHICH WAS SEVERE		1					ART only		Severe	
	406	SEVERE PRURITUS		1							Severe	

	175	[DESELECTED - ITCHING OF SKIN AFTER TAKING ORAL CHLOROQUINE], ANOTHER GOT RASH AFTER TAKING ORAL COARTEM		1					Antimal only	Oral&Topical	Unmentioned	
	1241	PATIENT RECEIVED IV METHOTREXATE, ADRIANYCIN & VINCRISTINE GOT SEVERE DIARRHOEA, ORAL STOMATITIS (NEUTROPENIC)	1						Other	Injectable	Severe	
	1618	[DESELECTED - TWO PATIENTS GOT ITCHY RASH 20 MINUTES AFTER TRAMADOL INJECTION, 1 PATIENT DEVELOPED ITCHY RASH AFTER IM DICLOFENAC], 1 PATIENT GOT RASH AFTER IV CIPROFLOXACIN		1					Antibact only	Injectable	Unmentioned	
	1214	PATIENT REACTED TO IV CEFTRIAXONE GOT PRURITUS AND ANGIOEDEMA INVOLVING THE MANDIBULAR AREA		1	1				Antibact only	Injectable	Unmentioned	
	844	SJS		1							Unmentioned	
	1807	ISS PATIENT WITH SKIN RASH,SCALING,ULCERATIONS FOR 2WKS THAT STARTED 1WK AFTER ART INITIATION OF COMBIVIR-NEVIRAPINE (AZT/3TC/NVP)		1					ART only		Unmentioned	
	1213	NAUSEA,VOMITING	1								Unmentioned	
