# Supplementary material for: Rare, Serious, and Comprehensively Described Suspected Adverse Drug Reactions Reported by Surveyed Healthcare Professionals in Uganda
Source: PLoS One. 2015 Apr 23;10(4):e0123974. doi: 10.1371/journal.pone.0123974 (PMC4408100; doi:10.1371/journal.pone.0123974)
Supplement: S6 Appendix — (PDF) [file pone.0123974.s006.pdf]

Appendix S6: Survey-descriptions of 106 serious suspected Adverse Drug Reactions (ADRs) of Healthcare Professionals (HCPs) who suspected ADRs in the past 4 weeks										
file	id	Level H/Facility	Type H/Facility	district	Region	Cadre	Nurse-cadre	HCP-Gender	HCP-Age	Serious ADR Description
		**Antibacterials Only**								
466	464	Private For-Profit	Health Centre III	GULU	Other/NK	Other		Male	26	32YR/FEMALE KNOWN ISS PATIENT ON COTRIMOXAZOLE CAME WITH SEVERE BODY RASHES & SLOUGHING*
243	1254	Private Not-for-Profit	Other	KAMPALA	Central	Nurse	Registered Nurse	Male	33	42YR/FEMALE SERO-POSITIVE, NAIVE TO HAART WITH MILD ITCHY RASH, APPETITE LOSS ,RISE IN BODY TEMPERATURE & ITCHING WHICH INTENSIFIED ON SWALLOWING SEPTRIN TABLETS FIVE WEEKS AGO. PATIENT RETURNED TO FACILITY & LAB INVESTIGATIONS SHOWED DERRANGED
1043	1074	Public	Health Centre IV	TORORO	Eastern	Nurse	Registered Midwife	Female	35	28YR OLD PATIENT WAS ADMINISTERED WITH COTRIMOXAZOLE TABLETS DEVELOPED BLACK PATCHES ON SKIN - MODERATE
1040	1071	Private For-Profit	Private Hospital	TORORO	Eastern	Doctor		Female	28	A MAN DIAGNOSED WITH HIV TOOK COTRIMOXAZOLE AND GOT STEVENS-JOHNSON SYNDROME - SEVERE
957	142	Public	Health Centre III	MITOOMA	Other/NK	Other		Male	27	HIV+ PATIENT WITH BODY ACHES, SUDDEN SKIN RASH, BURNT SKIN, OOZING OF SWEAT-LIKE FLUID AFTER COTRIMOXAZOLE ADMINISTRATION - MILD SYMPTOMS
550	2041	Public	National Referral	KAMPALA	Central	Other		Male	32	62YR/FEMALE ON COTRIMOXAZOLE ORAL ROUTE WITHIN TWO DAYS GOT MULTIPLE SKIN PATCHES, DEVELOPED SORES ON MUCOUS MEMBRANES WITH HIGH TEMPERATURE. GIVEN STEROIDS & SHE RECOVERED
990	1022	Private Not-for-Profit	Private Hospital	TORORO	Eastern	Other		Female	25	38YR/MALE ON SEPTRIN DEVELOPED SJS - MODERATE
614	832	Public	District Hospital	MASINDI	Other/NK	Nurse	Registered Nurse Midwife	Female	43	40YRS,SEPTRIN,ORALLY.SJS
738	637	Public	Health Centre III	JINJA	Eastern	Nurse	Enrolled Nurse	Female	20	26YR/MALE HIV+ ON ORAL SEPTRIN,GOT BURNT FACE & LIPS-GIVEN ORAL DEXAMETHASONE FOR FIVE DAYS - SEVERE
1008	1039	Private For-Profit	Private Hospital	TORORO	Eastern	Nurse	Nursing Assistant	Female	20	18YR OLD GIVEN IV CEFTRIAXONE. DRUG INJECTED VERY FAST & THE PATIENT DEVELOPED SEVERE VOMITING
758	657	Public	Health Centre III	JINJA	Eastern	Pharm		Female	26	25YR OLD FEMALE ON CIPROFLOXACIN IV. GOT SJS.IT WAS SEVERE
53	906	Private For-Profit	Private Hospital	KAMPALA	Central	Doctor		Male	45	REDUCED BLOOD SUGAR & LOW PULSE AFTER TAKING CHLORAMPHENICOL INJECTION
205	949	Public	Regional Referral	KAMPALA	Central	Doctor		Male	28	26YR ANAEMIC FEMALE WITH SEVERE PRE-ECLAMPSIA FINALLY DELIVERED & WAS MANAGED POST-OPERATIVELY WITH GENTAMICIN-SUSTAINED ACUTE RENAL FAILURE WITH ANAEMIA FOR 5 DAYS & OTHER COMPLICATIONS
		**Antiretrovirals Only**								
183	818	Public	National Referral	KAMPALA	Central	Nurse	Registered Nurse	Female	32	50YR FEMALE PATIENT GOT NEVIRAPINE (ORAL) HYPERSENSITIVITY INVOLVING ALL MUCUS MEMBRANES,SEVERE BUT RESOLVED
191	2004	Public	National Referral	KAMPALA	Central	Doctor		Female	32	36YR MALE ADMITTED WITH SJS FOLLOWING INITIATION OF ORAL NEVIRAPINE,SEVERE EVENT*
	1807	Public	National Referral	KAMPALA	Central	Doctor		Male	29	ISS PATIENT WITH SKIN RASH,SCALING,ULCERATIONS FOR 2WKS THAT STARTED 1WK AFTER ART INITIATION OF COMBIVIR-NEVIRAPINE (AZT/3TC/NVP)
	2019	Private Not-for-Profit	Private Hospital	WAKISO	Central	Doctor		Male	27	HIV+/FEMALE NEWLY ENROLLED ON HAART WITH NVP-BASED REGIMEN DEVELOPED SJS WHICH WAS SEVERE
409	1368	Private For-Profit	Private Hospital	KIRYANDO	Other/NK	Other		Female	27	35YR/FEMALE, WITH CD4 100 CELLS/DL ON AZT/3TC/NVP,AFTER 2WEEKS SHE CAME BACK WITH A SEVERE GENERALIZED BODY RASH
1157	882	Public	Health Centre III	MASAKA	Other/NK	Other		Female	27	32YR OLD REACTED TO ORAL NEVIRAPINE - SJS. REACTION WAS MODERATE

63	916	Private Not-for-Profit	Health Centre IV	KAMPALA	Central	Doctor		Female	32	NVP SKIN HYPERSENSITIVITY
1057	1088	Public	District Hospital	TORORO	Eastern	Nurse	Registered Nurse	Female	48	32YR/FEMALE ISS PATIENT ON AZT/3TC/NVP FOR 3 MONTHS GOT SKIN RASH ALL OVER THE BODY - STEVEN JOHNSON SYDROME - SEVERE
495	863	Private Not-for-Profit	Private Hospital	KAMPALA	Central	Doctor		Male	24	38YR OLD FEMALE WITH SEVERE NEVIRAPINE HYPERSENSITIVITY
1009	1040	Private For-Profit	Other	TORORO	Eastern	Other		Male	32	45YR/FEMALE DEVELOPED DEEP JAUNDICE (HEPATOTOXICITY) AFTER RECEIVING ORAL NEVIRAPINE. WAS GIVEN EFV AS ALTERNATIVE DRUG AND SHE IMPROVED
187	2008	Public	District Hospital	KAMPALA	Central	Doctor		Female	33	28YR FEMALE ON ORAL DUOVIR-N (AZT/3TC/NVP), GOT RASH WHICH WORSENED & SHE DEVELOPED SJS
910	1202	Private For-Profit	Private Hospital	MBRA	Other/NK	Pharm		Male	27	24 YR-OLD WITH SKIN PEELING OFF DUE TO NEVIRAPINE, REACTION WAS SEVERE
326	2035	Private For-Profit	Private Hospital	WAKISO	Central	Doctor		Female		ORAL ADMINISTRATION OF NEVIRAPINE IN PATIENT LEADING TO HEPATOTOXITY
563	781	Public	District Hospital	MASINDI	Other/NK	Other		Male	25	27YR OLD WITH 237 CD4 CELLS/ML ON NEVIRAPINE ORAL ROUTE GOT SEVERE RASH
951	136	Public	Health Centre IV		Other/NK	Pharm		Female	27	35 YR-OLD ISS PATIENT ON AZT/3TC/NVP GOT SKIN RASH MORE SEVERE ON LOWER LIMBS & SKIN STARTED PEELING OFF,DID NOT RESPOND TO STEROIDS OR ANTIFUNGALS
123	1839	Private For-Profit	Private Hospital	KAMPALA	Central	Other		Male	27	ORAL NEVIRAPINE INDUCED RASH-SJS IN 26YR PATIENT*
612	830	Public	District Hospital	MASINDI	Other/NK	Nurse	Registered Nurse	Female	33	45YR PATIENT ON ORAL TDF/3TC/NVP GOT SEVERE ABDOMINAL PAIN WHICH WAS IN COLICKY FORM
1161	886	Public	Regional Referral	MASAKA	Other/NK	Other		Female	42	NVP RASHES*
39	1625	Public	National Referral	KAMPALA	Central	Doctor		Male	28	PATIENT ON POST-EXPOSURE-PROPHYLAXIS (CBV/EFV) PRESENTS WITH EUPHORIA & DECREASED SLEEP: EFV-INDUCED NEUROPSYCHIATRIC MOOD
370	109	Private Not-for-Profit	District Hospital	BUIKWE	Central	Nurse	Registered Nurse	Female	25	MILD ADR.PATIENT ON ORAL EFV HAD GYNAECOMASTIA AND JERKS
887	1179	Private For-Profit	Private Hospital	MBRA	Other/NK	Pharm		Male	30	35YRS ORAL EFV LED TO MODERATE MENTAL DISTURBANCE, SUBSTITUTN REDUCED IT
1067	1098	Public	District Hospital	TORORO	Eastern	Doctor		Female	28	4YR HIV+ CHILD ON PROTEASE INHIBITORS SWITCHED TO ORAL EFV - SEVERE BEHAVIORAL DISTURBANCE INCLUDING AGGRESSION, INSOMNIA & HYPERACTIVITY
403	1287	Public	Health Centre III	KAMPALA	Central	Doctor		Female	39	PERIPHERAL NEUROPATHY DUE TO AZT
170	64	Private For-Profit	Private Hospital	KAMPALA	Central	Doctor		Male	24	25YR-OLD ANAEMIC, AZT
613	831	Public	District Hospital	MASINDI	Other/NK	Other		Female	36	39YR/FEMALE ON ZIDOVIDINE ORAL ROUTE GOT ANAEMIA. SHE WAS BLOOD TRANSFUSED & AZT WAS REPLACED WITH TENOFOVIR
603	821	Public	District Hospital	MASINDI	Other/NK	Other		Male	38	35YR ON AZT-CONTAINING REGIMEN ORAL ROUTE DEVELOPED SEVERE ANAEMIA LEADING TO ADMISSION & BLOOD TRANSFUSION
423	419	Private Not-for-Profit	Regional Referral	GULU	Other/NK	Pharm		Female	26	22YR/FEMALE LADY NURSE WHO GOT NEEDLE STICK INJURY & WAS INITIATED ON PEP WITH AZT/3TC-12 DAYS LATER GOT SEVERE RASH (SJS) & WAS ADMITED AND TREATED
628	526	Public	District Hospital	KAMULI	Eastern	Other		Male	30	37YR OLD GOT SJS AFTER TAKING ARVS. WAS LATER GIVEN IV FLUID ANTIBIOTICS, STEROID DRUGS & SUPPORTIVE TREATMENT
69	922	Public	National Referral	KAMPALA	Central	Nurse	Registered Nurse Midwife	Female		30YR PATIENT REACTED TO ART ADMINISTERED ORALLY. PATIENT LOST SKIN & MUCUS MEMBRANES-SEVERE
430	426	Private Not-for-Profit	Private Hospital	GULU	Other/NK	Nurse	Registered Nurse	Female	36	27YR/FEMALE ON PEP ORAL ROUTE GOT BLISTERS 3 DAYS LATER WITH BURNING SENSATION ALL-OVER THE BODY.MODERATE
1034	1065	Private Not-for-Profit	District Hospital	TORORO	Eastern	Nurse	Registered Nurse	Male	25	MOTHER REPORTED CONVULSIONS WHENEVER THE CHILD TOOK HIS ORAL ARVS - MODERATE

		**Antimalarials Only**								
992	1024	Private Not-for-Profit	Private Hospital	TORORO	Eastern	Nurse	Enrolled Nurse	Female	27	A CHILD OF 5YRS AFTER INTRAVENOUS QUININE. SHE DIED AFTER ARRIVAL#
869	767	Public	Regional Referral	JINJA	Eastern	Nurse	Registered Nurse	Female	32	3YR OLD CHILD GIVEN IV QUININE & SHE BECAME HYPOGLYCAEMIC
986	1017	Private Not-for-Profit	Private Hospital	TORORO	Eastern	Nurse	Enrolled Comprehensive N	Male	24	2YR OLD GIVEN IM QUININE DEVELOPED HYPOGLYCAEMIA - IMPROVED AFTER MANAGEMENT - SEVERE
624	522	Public	District Hospital	KAMULI	Eastern	Nurse	Enrolled Nurse	Male	47	2YR OLD BOY WAS BROUGHT TO HOSPITAL WITH A REACTION TO QUININE & DOCTOR GAVE HIM AN ANTIDOTE BUT HE DIED
914	1206	Private Not-for-Profit	Private Hospital	MBRA	Other/NK	Nurse	Enrolled Nurse	Female	60	17YR-OLD ON IM QNN WITH POST INJECTION PARALYSIS, IT WAS SEVERE
26	1810	Private For-Profit	Health Centre III	KAMPALA	Central	Doctor		Male	35	TINNITUS IN A 27YR-OLD AFTER IV QUININE. SEVERE (LED TO ALTERED CONSCIOUSNESS).
888	1180	Private Not-for-Profit	Private Hospital	MBRA	Other/NK	Nurse	Enrolled Nurse	Female	50	PATIENT ON ORAL QNN GOT SKIN RASH,TINNITUS,ABORTION,VERTIGO, NAUSEA, VOMITING, BLURRED VISION - SEVERE
851	749	Private For-Profit	Drug Shop	JINJA	Eastern	Nurse	Registered Midwife	Male	30	28YR OLD FEMALE REACTED TO QUININE IV LEADING TO MISCARRIAGE
799	698	Private Not-for-Profit	Health Centre IV	JINJA	Eastern	Doctor		Male	24	52YR OLD FEMALE ON COARTEM ORAL ROUTE DEVELOPED SORES ON THE WHOLE BODY
359	97	Private Not-for-Profit	Other	BUIKWE	Central	Doctor		Male	52	PATIENT 52YR B/S +VE FOR MALARIA PARASITES. GIVEN COARTEM FOR THREE DAYS' TREATMENT, AFTER 1 DAY GOT SKIN ITCHING & SEVERE RASH.STOPPED DRUG, GAVE IV QNN & PATIENT IMPROVED
49	902	Public	Health Centre IV	KAMPALA	Central	Doctor		Male		SEVERE HYPOGLYCEMIA IN ADULT-DRUG WAS ARTESUNATE/AMODIAQUINE
570	788	Private For-Profit	Health Centre IV	MASINDI	Other/NK	Doctor		Male	62	62YR/FEMALE ON ORAL MEPHAQUINE GOT SEVERE HEADACHE WITH MENTAL CONFUSION & INSOMNIA
		**Antibacterials & Antimalarials**								
160	1874	Private For-Profit	Health Centre IV	KAMPALA	Central	Doctor		Female		19YR FEMALE PATIENT WITH MALARIA & COUGH REACTED SEVERELY TO COMBINATION OF QNN-IV & ORAL SEPTRIN. MANAGED WITH PARENTERAL HYDROCORTISONE
996	1027	Private Not-for-Profit	Private Hospital	TORORO	Eastern	Nurse	Enrolled Comprehensive N	Female	30	5YR OLD GIRL FROM A CLINIC WHERE SHE WAS PUT ON IV QUININE & SEPTRIN. GOT BLISTERS ALL OVER THE BODY & DIED ON ADMISSION - SEVERE
988	1020	Private Not-for-Profit	Private Hospital	TORORO	Eastern	Nurse	Enrolled Comprehensive N	Female	27	8YR OLD GIRL REFERRED FROM A CLINIC AFTER RECEIVING IV QUININE & SEPTRIN DEVELOPED BLISTERS ALL-OVER THE BODY & DIED ON ADMISSION - SEVERE
1047	1078	Private Not-for-Profit	Private Hospital	TORORO	Eastern	Nurse	Nursing Assistant	Female	29	[illegible]YR OLD GIRL WAS REFERRED FROM A CLINIC AFTER TAKING IV QUININE & SEPTRIN WITH BLISTERS ALL-OVER THE BODY. SHE DIED ON ADMISSION - SEVERE
		**Antituberculosis drugs Only**								
502	1008	Private Not-for-Profit	Private Hospital	KAMPALA	Central	Nurse	Enrolled Comprehensive N	Male	30	40YR OLD ON ETHAMBUTOL ORAL ROUTE GOT BURNING SENSATION OF BOTH LIMBS & LOSS OF SIGHT - MODERATE SEVERITY
		**Antibacterials & Antituberculosis drugs**								
197	941	Private Not-for-Profit	Private Hospital	KAMPALA	Central	Doctor		Male	26	24YR/FEMALE KNOWN IMMUNOSUPPRESSED SYNDROME (ISS) PATIENT ON ANTI-TBS WHO REACTED TO COTRIMOXAZOLE - SJS & ALSO HAD TOXOPLASMOSIS
		**Analgesics Only**								
278	2029	Private For-Profit	Private Hospital	KAMPALA	Central	Doctor		Male	28	ORAL DICLOFENAC 50MG, HAEMOPTYSIS AFTER 2 DAYS - WAS SEVERE*
927	1219	Private For-Profit	Pharmacy	MBRA	Other/NK	Pharm		Male	25	GASTROINTESTINAL DISTRESS DUE TO NSAID-INDUCED ULCERATION AFTER ORAL DICLOFENAC

	16	Public	National Referral	KAMPALA	Central	Pharm		Male	25	PATIENT DEVELOPED PEPTIC ULCER DISEASE A FEW DAYS AFTER TAKING ACECLOFENAC
511	484	Public	Regional Referral	LIRA	Other/NK	Pharm		Male	38	26YR/FEMALE HAD A HISTORY OF PEPTIC ULCER DISEASE - DUE TO INDOMETHACIN BUT WAS NOT SEVERE
453	450	Public	National Referral	GULU	Other/NK	Doctor		Female	35	41YR/MALE GIVEN PANADOL (PARACETAMOL), STARTED SHAKING & SWEATING 10 MINUTES LATER.WAS PUT ON A DRIP AND HE BECAME FINE.
		**Antibacterials & Analgesics**								
301	1650	Private For-Profit	Health Centre III	KAYUNGA	Central	Nurse	Enrolled Nurse	Female		ELDERLY PATIENT ON PIROXICAM COMPLAINED OF HEART PAIN WHICH INDICATED PUD & ALSO GOT OEDEMA DUE TO PENICILLINS
		**Antibacterials & Antivirals**								
913	1205	Private Not-for-Profit	Private Hospital	MBRA	Other/NK	Doctor		Male	41	74YR-OLD WITH CORNEAL ULCER NOT RESPONDING TO TOPICAL ANTIBIOTICS & ANTIVIRALS FOR 14 DAYS. STOPPED THE MEDICATION AND LEFT PATIENT ON ARTIFICIAL TEARS ONLY & NOTED GREAT IMPROVEMENT
		**Other Medications**								
110	1252	Public	National Referral	KAMPALA	Central	Nurse	Enrolled Midwife	Female	51	PROLONGED HEAVY FLOW,INCREASED BLOOD PRESSURE & PAIN OF ARM WHILE USING IMPLANTS
710	608	Private For-Profit	Health Centre IV	KAMULI	Eastern	Nurse	Nursing Assistant	Female	28	30 YR FEMALE REACTED TO INJECTAPLAN,OVER BLEEDING. I PRESCRIBED IBRUPROFEN & AMOXICILLIN FOR FOUR DAYS & BLEEDING STOPPED
508	481	Public	Regional Referral	LIRA	Other/NK	Doctor		Female	35	22YR/FEMALE ON INJECTAPLAN CAME BACK AFTER 1 WEEK WITH CONSTANT BLEEDING. DISCONTINUED USE OF THE INJECTAPLAN
556	774	Private For-Profit	Other	MASINDI	Other/NK	Other	Nursing Assistant	Male	24	EXCESSIVE VAGINAL BLEEDING IN 26YR OLD PATIENT GIVEN SUBCUTANEOUS NORPLANTS
870	768	Private For-Profit	Health Centre IV	JINJA	Eastern	Nurse	Nursing Assistant	Female	28	WOMEN REACTING TO INJECTAPLAN AND PILLPLAN WITH EXCESSIVE BLEEDING
591	809	Private For-Profit	Health Centre IV	MASINDI	Other/NK	Doctor		Male	36	FEMALE 25YRS WITH IMPLANT,EXCESSIVE UTERINE BLEEDING, MODERATE
	1241	Public	National Referral	KAMPALA	Central	Nurse	Registered Nurse	Female	45	[illegible] DIARRHOEA, ORAL STOMATITIS (NEUTROPENIC)
	1248	Public	National Referral	KAMPALA	Central	Nurse	Registered Nurse	Female	25	SEVERE MUCOSITIS IN PATIENTS WHO GOT 5-FLUOROURACIL (ANTICANCER AGENT)
	1246	Public	National Referral	KAMPALA	Central	Nurse	Registered Nurse Midwife	Female	45	PATIENT AFTER CHEMOTHERAPHY GOT SEVERE SKIN REACTION AFTER 4WEEKS OF TREATMENT. SITE WAS LEFT HARD
	1223	Public	National Referral	KAMPALA	Central	Nurse	Registered Nurse Midwife	Female	45	PATIENT WITH BURNT LEG WHEN PUT ON CHEMOTHERAPHY,HE GOT PAIN & LEG HAD WOUNDS
429	425	Private Not-for-Profit	Private Hospital	GULU	Other/NK	Nurse	Enrolled Comprehensive N	Male	26	13YR/MALE ON PREDNISOLONE ORAL ROUTE GOT SEVERE EPIGASTRIC PAIN WHICH WAS MANAGED AS SEVERE STEROID-INDUCED GASTRITIS
104	983	Public	National Referral	KAMPALA	Central	Nurse	Registered Nurse Midwife	Female	54	ALCOHOL 95% ADMINISTERED IN THE EYE OF A PATIENT
	833	Public	National Referral	KAMPALA	Central	Nurse	Registered Nurse Midwife	Female	31	SEVERE PALPITATIONS DUE TO OVER DOSE OF OMEPRAZOLE
234	978	Private For-Profit	Other	KAMPALA	Central	Doctor		Male	32	35YR/MALE ON IV LIGNOCAINE & ADRENALINE. GOT SWOLLEN & WAS FAILING TO BREATH-SEVERE
	13	Public	National Referral	KAMPALA	Central	Doctor		Female	25	SJS SECONDARY TO CARBAMAZEPINE
167	61	Private For-Profit	Private Hospital	KAMPALA	Central	Doctor		Male	30	30YR PRIME GRAVID GIVEN MAGNESIUM SULPHATE TO PREVENT FITS-COMPLAINED OF EXCESSIVE HEAT & STARTED SWEATING WITH CHANGE IN HAEMODYNAMIC STABILITY
704	602	Private For-Profit	Pharmacy	KAMULI	Eastern	Nurse	Enrolled Nurse	Male	32	24YR OLD BOY TOOK OVERDOSE OF CHLORPHENIRAMINE & FELL ASLEEP FOR A FULL DAY.

1007	1038	Private Not-for-Profit	Private Hospital	TORORO	Eastern	Nurse	Enrolled Mental Health Nu	Female	20	27YR/MALE WITH PROTRUDING TONGUE AFTER ORAL CHLORPROMAZINE - MILD
89	1628	Public	National Referral	KAMPALA	Central	Doctor		Female	43	14YR OLD GIRL ORALLY ADMINISTERD HALOPERIDOL -GOT EXTRAPYRAMIDAL SIDE EFFECTS, MODERATE. I REDUCED DOSE AND GAVE BENZHEXOL
		**No Medication Reported**								
	1247	Public	National Referral	KAMPALA	Central	Nurse	Registered Nurse	Female	27	SJS, MUCOSITIS (WHERE PATIENT CANNOT PUT ANYTHING IN THE MOUTH), DROP IN BLOOD COUNTS
	844	Public	National Referral	KAMPALA	Central	Doctor		Male	26	SJS
	1632	Private For-Profit	Private Hospital	KAMPALA	Central	Doctor		Male	27	SJS
	816	Public	National Referral	KAMPALA	Central	Doctor		Male	26	SJS
	830	Public	National Referral	KAMPALA	Central	Doctor		Female	39	MUSCLE RIGIDITY AND EXCESSIVE SALIVATION
20	839	Public	National Referral	KAMPALA	Central	Other		Male	56	EXTRAPYRAMIDAL SIDE EFFECTS E.G. TREMORS,TARDIVE DYSKINESIA, AKATHISIA ETC
	1228	Public	National Referral	KAMPALA	Central	Other	Registered Comprehensive	Female	34	ROLLING EYES,BODY TREMORS, SALIVA THROUGH THE MOUTH,TONGUE OUT,BODY WEAKNESS
	1229	Public	National Referral	KAMPALA	Central	Other	Other	Male	32	TREMORS OF THE HANDS & ENTIRE BODY,COMING OUT OF TONGUE,ROLLING EYES,BODY WEAKNESS
	1203	Public	National Referral	KAMPALA	Central	Doctor		Female	26	SULPHUR-ASSOCIATED SKIN BULLOUS ERUPTIONS (FDR KIND)
	838	Public	National Referral	KAMPALA	Central	Doctor		Male	46	HEPATOTOXICITY IN TB PATIENT*
	1222	Public	National Referral	KAMPALA	Central	Nurse	Registered Nurse	Male		OTOTOXITY
	1243	Public	National Referral	KAMPALA	Central	Nurse	Registered Mental Health N	Female	40	EXTENSIVE ORAL SORES WITH SEVERE DIARRHOEA &GENERALISED BODY RASH
247	1259	Public	National Referral	KAMPALA	Central	Doctor		Male		25YR WITH SEVERE ADR THAT THE SKIN & MUCOUS MEMBRANES WITH REDDENING OF EYES
679	577	Private For-Profit	Drug Shop	KAMULI	Eastern	Nurse	Nursing Assistant	Female	29	ANAPHYLAXIS,MENTAL EFFECT,ALLERGIC REACTION,HEADACHE,ABDOMINAL DISCOMFORT
	6	Public	National Referral	KAMPALA	Central	Pharm		Female	24	BLOOD IN URINE
933	1225	Public	Health Centre III	MBRA	Other/NK	Other		Male	47	BODY SWELLING WITH ANEMIA
	602	Public	National Referral	KAMPALA	Central	Nurse	Registered Nurse	Female	36	SKIN PEELING WHICH COULD LEAVE SCARS
	1218	Public	National Referral	KAMPALA	Central	Nurse	Registered Nurse	Female	26	SEVERE NEUTROPENIA WITH VERY HIGH UNCONTROLLED TEMPERATURE, SEVERE MUCOSITIS, EXCESSIVE VOMITING, EXCESSIVE WEIGHT LOSS & MALNUTRITION,TUMOR LYSIS SYNDROME

KEY

ADR = ADVERSE DRUG REACTION
ART = ANTIRETROVIRAL THERAPY
AZT = ZIDOVUDINE
HAART = HIGHLY ACTIVE ANTIRETROVIRAL THERAPY
FLAGYL = METRONIDAZOLE
EFV = EFAVIRENZ
3TC = LAMIVUDINE
ISS = IMMUNOSUPRESSED SYNDROME

IV = INTRAVENOUS
NVP = NEVIRAPINE
MEPHAQUINE = MEFLOQUINE
QNN = QUININE
SEPTRIN = COTRIMOXAZOLE
SJS = STEVENS-JOHNSON SYNDROME
TDF = TENOFOVIR
* = CHECK APPENDIX - 2 OR MORE ADVERSE REACTIONS DESCRIBED

= MAY BE THE SAME CHILD AS IN THE 3 FATAL DESCRIPTIONS INVOLVING QUININE & SEPTRIN EXCEPT THAT SEPTRIN WAS NOT MENTIONED
