# Supplementary material for: Rare, Serious, and Comprehensively Described Suspected Adverse Drug Reactions Reported by Surveyed Healthcare Professionals in Uganda
Source: PLoS One. 2015 Apr 23;10(4):e0123974. doi: 10.1371/journal.pone.0123974 (PMC4408100; doi:10.1371/journal.pone.0123974)
Supplement: S7 Appendix — (PDF) [file pone.0123974.s007.pdf]

| Appendix S7: Survey-descriptions of 59 severe suspected Adverse Drug Reactions (ADRs) by Healthcare Professionals (HCPs) who suspected ADRs in the past 4 weeks | | | | | | | | | | |
|---|---|---|---|---|---|---|---|---|---|---|
| file | id | Level H/Facility | Type H/Facility | District | Region | Cadre | Nurse-cadre | HCP-Gender | HCP-Age | Severe ADR Description |
| | | **Antibacterials Only** | | | | | | | | |
| 304 | 1653 | Public | District Hospital | KAYUNGA | Central | Nurse | Registered Midwife | Female | 42 | 28YRS, COTRIMOXAZOLE, SEVERE |
| 466 | 464 | Private For-Profit | Health Centre III | GULU | Other/NK | Other | | Male | 26 | 32YR/FEMALE KNOWN ISS PATIENT ON COTRIMOXAZOLE CAME WITH SEVERE BODY RASHES & SLOUGHING* |
| 1040 | 1071 | Private For-Profit | Private Hospital | TORORO | Eastern | Doctor | | Female | 28 | A MAN DIAGNOSED WITH HIV TOOK COTRIMOXAZOLE AND GOT STEVENS-JOHNSON SYNDROME - SEVERE |
| 919 | 1211 | Private For-Profit | Health Centre III | MBRA | Other/NK | Doctor | | Male | 47 | 32YR OLD HIV+ MALE STARTED ON COTRIMOXAZOLE ORALLY. IT WAS SEVERE.RECOVERED |
| 738 | 637 | Public | Health Centre III | JINJA | Eastern | Nurse | Enrolled Nurse | Female | 20 | 26YR/MALE HIV+ ON ORAL SEPTRIN,GOT BURNT FACE & LIPS-GIVEN ORAL DEXAMETHASONE FOR FIVE DAYS - SEVERE |
| 991 | 1023 | Private Not-for-Profit | Private Hospital | TORORO | Eastern | Nurse | Enrolled Comprehensive | Male | 20 | A MAN 35YRS WITH GENERALIZED SKIN RASH AFTER TAKING ORAL SEPTRIN - SEVERE |
| 1061 | 1092 | Public | District Hospital | TORORO | Eastern | Nurse | Nursing Assistant | Female | 50 | A 20YR OLD TAKING SEPTRIN ORALLY GOT SEVERE SKIN RASH ALL OVER THE BODY |
| 349 | 86 | Private For-Profit | Health Centre IV | BUIKWE | Central | Nurse | Enrolled Comprehensive | Male | 25 | 58YR OLD ON ORAL COTRIMOXAZOLE GOT SEVERE URTICARIA AND SKIN RASHES ALL OVER THE BODY |
| 417 | 1692 | Private For-Profit | Health Centre IV | KAMPALA | Central | Nurse | Enrolled Nurse | Male | 25 | ADULT WITH UTI GIVEN ORAL CIPROFLOXIN GOT ARTHRALGIA, MODERATE ABDOMINAL PAIN,VOMITING & RESTLESSNESS. SEVERE ADR. |
| 758 | 657 | Public | Health Centre III | JINJA | Eastern | Pharm | | Female | 26 | 25YR OLD FEMALE ON CIPROFLOXACIN IV. GOT SJS.IT WAS SEVERE |
| 608 | 826 | Public | District Hospital | MASINDI | Other/NK | Other | | Male | | 28YR/MALE - ALLERGIC REACTION TO ORAL CIPROFLOXACIN - SEVERE URTICARIA - SUBSTITUTED FOR CEPHALEXIN & CETIRIZINE* |
| 325 | 2034 | Private For-Profit | Private Hospital | WAKISO | Central | Nurse | Enrolled Comprehensive | Female | | 18YR/FEMALE REACTED TO INTRAVENOUS CEFTRIAXONE BY SWELLING OF HAND THROUGH WHICH DRUG WAS GIVEN & SEVERE BACK PAIN-MILD |
| 1008 | 1039 | Private For-Profit | Private Hospital | TORORO | Eastern | Nurse | Nursing Assistant | Female | 20 | 18YR OLD GIVEN IV CEFTRIAXONE. DRUG INJECTED VERY FAST & THE PATIENT DEVELOPED SEVERE VOMITING |
| | | **Antiretrovirals Only** | | | | | | | | |
| 183 | 818 | Public | National Referral | KAMPALA | Central | Nurse | Registered Nurse | Female | 32 | 50YR FEMALE PATIENT GOT NEVIRAPINE (ORAL) HYPERSENSITIVITY INVOLVING ALL MUCUS MEMBRANES,SEVERE BUT RESOLVED |
| 191 | 2004 | Public | National Referral | KAMPALA | Central | Doctor | | Female | 32 | 36YR MALE ADMITTED WITH SJS FOLLOWING INITIATION OF ORAL NEVIRAPINE,SEVERE EVENT* |
| | 2019 | Private Not-for-Profit | Private Hospital | WAKISO | Central | Doctor | | Male | 27 | HIV+/FEMALE NEWLY ENROLLED ON HAART WITH NVP-BASED REGIMEN DEVELOPED SJS WHICH WAS SEVERE |
| 409 | 1368 | Private For-Profit | Private Hospital | KIRYANDON | Other/NK | Other | | Female | 27 | 35YR/FEMALE, WITH CD4 100 CELLS/DL ON AZT/3TC/NVP,AFTER 2WEEKS SHE CAME BACK WITH A SEVERE GENERALIZED BODY RASH |
| 1057 | 1088 | Public | District Hospital | TORORO | Eastern | Nurse | Registered Nurse | Female | 48 | 32YR/FEMALE ISS PATIENT ON AZT/3TC/NVP FOR 3 MONTHS GOT SKIN RASH ALL OVER THE BODY - STEVEN JOHNSON SYDROME - SEVERE |
| 495 | 863 | Private Not-for-Profit | Private Hospital | KAMPALA | Central | Doctor | | Male | 24 | 38YR OLD FEMALE WITH SEVERE NEVIRAPINE HYPERSENSITIVITY |
| 910 | 1202 | Private For-Profit | Private Hospital | MBRA | Other/NK | Pharm | | Male | 27 | 24 YR-OLD WITH SKIN PEELING OFF DUE TO NEVIRAPINE, REACTION WAS SEVERE |
| 563 | 781 | Public | District Hospital | MASINDI | Other/NK | Other | | Male | 25 | 27YR OLD WITH 237 CD4 CELLS/ML ON NEVIRAPINE ORAL ROUTE GOT SEVERE RASH |
| 1158 | 883 | Public | Regional Referral | MASAKA | Other/NK | Nurse | Registered Comprehensi | Female | | ORAL NEVIRAPINE, SEVERE HEADACHE, PATIENT STABILIZED |

| | | | | | | | | | | |
|---|---|---|---|---|---|---|---|---|---|---|
| 951 | 136 | Public | Health Centre IV | | Other/NK | Pharm | | Female | 27 | 35 YR-OLD ISS PATIENT ON AZT/3TC/NVP GOT SKIN RASH MORE SEVERE ON LOWER LIMBS & SKIN STARTED PEELING OFF,DID NOT RESPOND TO STEROIDS OR ANTIFUNGALS |
| 612 | 830 | Public | District Hospital | MASINDI | Other/NK | Nurse | Registered Nurse | Female | 33 | 45YR PATIENT ON ORAL TDF/3TC/NVP GOT SEVERE ABDOMINAL PAIN WHICH WAS IN COLICKY FORM |
| 1067 | 1098 | Public | District Hospital | TORORO | Eastern | Doctor | | Female | 28 | 4YR HIV+ CHILD ON PROTEASE INHIBITORS SWITCHED TO ORAL EFV - SEVERE BEHAVIORAL DISTURBANCE INCLUDING AGGRESSION, INSOMNIA & HYPERACTIVITY |
| 423 | 419 | Private Not-for-Profit | Regional Referral | GULU | Other/NK | Pharm | | Female | 26 | 22YR/FEMALE LADY NURSE WHO GOT NEEDLE STICK INJURY & WAS INITIATED ON PEP WITH AZT/3TC-12 DAYS LATER GOT SEVERE RASH (SJS) & WAS ADMITED AND TREATED |
| 603 | 821 | Public | District Hospital | MASINDI | Other/NK | Other | | Male | 38 | 35YR ON AZT-CONTAINING REGIMEN ORAL ROUTE DEVELOPED SEVERE ANAEMIA LEADING TO ADMISSION & BLOOD TRANSFUSION |
| 987 | 1019 | Private Not-for-Profit | Private Hospital | TORORO | Eastern | Nurse | Enrolled Comprehensive | Female | 22 | 38YR OLD IMMEDIATELY AFTER STARTING ORAL ARVS FELT LIKE HOT WATER HAD BEEN POURED ON HIM - SEVERE |
| 69 | 922 | Public | National Referral | KAMPALA | Central | Nurse | Registered Nurse Midwif | Female | | 30YR PATIENT REACTED TO ART ADMINISTERED ORALLY. PATIENT LOST SKIN & MUCUS MEMBRANES-SEVERE |
| | | **Antimalarials Only** | | | | | | | | |
| 986 | 1017 | Private Not-for-Profit | Private Hospital | TORORO | Eastern | Nurse | Enrolled Comprehensive | Male | 24 | 2YR OLD GIVEN IM QUININE DEVELOPED HYPOGLYCAEMIA - IMPROVED AFTER MANAGEMENT - SEVERE |
| 914 | 1206 | Private Not-for-Profit | Private Hospital | MBRA | Other/NK | Nurse | Enrolled Nurse | Female | 60 | 17YR-OLD ON IM QNN WITH POST INJECTION PARALYSIS, IT WAS SEVERE |
| 26 | 1810 | Private For-Profit | Health Centre III | KAMPALA | Central | Doctor | | Male | 35 | TINNITUS IN A 27YR-OLD AFTER IV QUININE. SEVERE (LED TO ALTERED CONSCIOUSNESS). |
| 888 | 1180 | Private Not-for-Profit | Private Hospital | MBRA | Other/NK | Nurse | Enrolled Nurse | Female | 50 | PATIENT ON ORAL QNN GOT SKIN RASH,TINNITUS,ABORTION,VERTIGO, NAUSEA, VOMITING, BLURRED VISION - SEVERE |
| 570 | 788 | Private For-Profit | Health Centre IV | MASINDI | Other/NK | Doctor | | Male | 62 | 62YR/FEMALE ON ORAL MEPHAQUINE GOT SEVERE HEADACHE WITH MENTAL CONFUSION & INSOMNIA |
| 49 | 902 | Public | Health Centre IV | KAMPALA | Central | Doctor | | Male | | SEVERE HYPOGLYCEMIA IN ADULT-DRUG WAS ARTESUNATE/AMODIAQUINE |
| 359 | 97 | Private Not-for-Profit | Other | BUIKWE | Central | Doctor | | Male | 52 | PATIENT 52YR B/S +VE FOR MALARIA PARASITES. GIVEN COARTEM FOR THREE DAYS' TREATMENT, AFTER 1 DAY GOT SKIN ITCHING & SEVERE RASH.STOPPED DRUG, GAVE IV QNN & PATIENT IMPROVED |
| | | **Antibacterials & Antimalarials** | | | | | | | | |
| 160 | 1874 | Private For-Profit | Health Centre IV | KAMPALA | Central | Doctor | | Female | | 19YR FEMALE PATIENT WITH MALARIA & COUGH REACTED SEVERELY TO COMBINATION OF QNN-IV & ORAL SEPTRIN. MANAGED WITH PARENTERAL HYDROCORTISONE |
| 996 | 1027 | Private Not-for-Profit | Private Hospital | TORORO | Eastern | Nurse | Enrolled Comprehensive | Female | 30 | 5YR OLD GIRL FROM A CLINIC WHERE SHE WAS PUT ON IV QUININE & SEPTRIN. GOT BLISTERS ALL OVER THE BODY & DIED ON ADMISSION - SEVERE |
| 988 | 1020 | Private Not-for-Profit | Private Hospital | TORORO | Eastern | Nurse | Enrolled Comprehensive | Female | 27 | 8YR OLD GIRL REFERRED FROM A CLINIC AFTER RECEIVING IV QUININE & SEPTRIN DEVELOPED BLISTERS ALL-OVER THE BODY & DIED ON ADMISSION - SEVERE |
| 1047 | 1078 | Private Not-for-Profit | Private Hospital | TORORO | Eastern | Nurse | Nursing Assistant | Female | 29 | 5YR OLD GIRL WAS REFERRED FROM A CLINIC AFTER TAKING IV QUININE & SEPTRIN WITH BLISTERS ALL-OVER THE BODY. SHE DIED ON ADMISSION - SEVERE |
| 253 | 994 | Public | National Referral | KAMPALA | Central | Nurse | Registered Nurse Midwif | Female | 60 | ADULT SEVERELY REACTED TO SEPTRIN AND TB DRUGS TAKEN ORALLY |
| | | **Analgesics Only** | | | | | | | | |
| 278 | 2029 | Private For-Profit | Private Hospital | KAMPALA | Central | Doctor | | Male | 28 | ORAL DICLOFENAC 50MG, HAEMOPTYSIS AFTER 2 DAYS - WAS SEVERE* |

| | | | | | | | | | | |
|---|---|---|---|---|---|---|---|---|---|---|
| 332 | 1672 | Private For-Profit | Private Hospital | KAMPALA | Central | Other | | Male | 30 | ORAL ROUTE OF TRAMADOL RESULTED INTO SEVERE ITCHY SORES ALL-OVER THE BODY THAT PERSISTED |
| | | **Other Medications** | | | | | | | | |
| 1162 | 887 | Public | Health Centre IV | MASAKA | Other/NK | Pharm | | Male | 30 | 40YR OLD SEVERE HEADACHE AFTER ORAL LOSARTAN |
| 429 | 425 | Private Not-for-Profit | Private Hospital | GULU | Other/NK | Nurse | Enrolled Comprehensive | Male | 26 | 13YR/MALE ON PREDNISOLONE ORAL ROUTE GOT SEVERE EPIGASTRIC PAIN WHICH WAS MANAGED AS SEVERE STEROID-INDUCED GASTRITIS |
| | 833 | Public | National Referral | KAMPALA | Central | Nurse | Registered Nurse Midwif | Female | 31 | SEVERE PALPITATIONS DUE TO OVER DOSE OF OMEPRAZOLE |
| | 1241 | Public | National Referral | KAMPALA | Central | Nurse | Registered Nurse | Female | 45 | PATIENT RECEIVED IV METHOTREXATE, ADRIANYCIN & VINCRISTINE GOT SEVERE DIARRHOEA, ORAL STOMATITIS (NEUTROPENIC) |
| 234 | 978 | Private For-Profit | Other | KAMPALA | Central | Doctor | | Male | 32 | 35YR/MALE ON IV LIGNOCAINE & ADRENALINE. GOT SWOLLEN & WAS FAILING TO BREATH-SEVERE |
| 498 | 1004 | Private For-Profit | Private Hospital | KAMPALA | Central | Nurse | Enrolled Nurse | Female | 29 | LADY IN LATE TWENTIES GIVEN IV HYDROCORTISONE,GOT SEVERE BURNING SENSATION IN PRIVATE PARTS IMMEDIATELY AFTER ADMINISTRATION OF THE DRUG |
| 1056 | 1087 | Public | District Hospital | TORORO | Eastern | Nurse | Registered Mental Healtl | Female | 32 | 18YR/MALE MAN TAKING ORAL PHENORBABITONE GOT RASHES ALL OVER THE BODY SEVERE |
| | 1248 | Public | National Referral | KAMPALA | Central | Nurse | Registered Nurse | Female | 25 | SEVERE MUCOSITIS IN PATIENTS WHO GOT 5-FLUOROURACIL (ANTICANCER AGENT) |
| | 1246 | Public | National Referral | KAMPALA | Central | Nurse | Registered Nurse Midwif | Female | 45 | PATIENT AFTER CHEMOTHERAPHY GOT SEVERE SKIN REACTION AFTER 4WEEKS OF TREATMENT. SITE WAS LEFT HARD |
| | | **No Medication Reported** | | | | | | | | |
| | 406 | Public | National Referral | KAMPALA | Central | Doctor | | Male | 26 | SEVERE PRURITUS |
| 228 | 972 | Public | National Referral | KAMPALA | Central | Doctor | | Male | 38 | 80YR/FEMALE WITH GENERALIZED SKIN ERUPTIONS & WOUNDS, NOT SURE OF DRUG BUT IT WAS SEVERE. ROUTE - ORAL. |
| | 1243 | Public | National Referral | KAMPALA | Central | Nurse | Registered Mental Healtl | Female | 40 | EXTENSIVE ORAL SORES WITH SEVERE DIARRHOEA &GENERALISED BODY RASH |
| 247 | 1259 | Public | National Referral | KAMPALA | Central | Doctor | | Male | | 25YR WITH SEVERE ADR THAT THE SKIN & MUCOUS MEMBRANES WITH REDDENING OF EYES |
| 174 | 68 | Private Not-for-Profit | Private Hospital | KAMPALA | Central | Nurse | Enrolled Midwife | Female | 28 | PATIENT WAS COMPLAINING OF SEVERE HEADACHE & I REFERED HIM TO A MEDICAL OFFICER |
| 504 | 1010 | Private Not-for-Profit | Private Hospital | WAKISO | Central | Other | | Male | | GENERALISED BODY RASH WITH SORES IN 30YR OLD MALE. REACTION WAS SEVERE |
| | 1218 | Public | National Referral | KAMPALA | Central | Nurse | Registered Nurse | Female | 26 | SEVERE NEUTROPENIA WITH VERY HIGH UNCONTROLLED TEMPERATURE, SEVERE MUCOSITIS, EXCESSIVE VOMITING, EXCESSIVE WEIGHT LOSS & MALNUTRITION,TUMOR LYSIS SYNDROME |

**KEY**

ADR = ADVERSE DRUG REACTION
ART = ANTIRETROVIRAL THERAPY
ARVS = ANTIRETROVIRAL DRUGS
AZT = ZIDOVUDINE
EFV = EFAVIRENZ
3TC = LAMIVUDINE
ISS = IMMUNOSUPRESSED SYNDROME

IV = INTRAVENOUS
NVP = NEVIRAPINE
MEPHAQUINE = MEFLOQUINE
SEPTRIN = COTRIMOXAZOLE
SJS = STEVENS-JOHNSON SYNDROME
TDF = TENOFOVIR
UTI = URINARY TRACT INFECTION

* = CHECK APPENDIX - 2 OR MORE ADVERSE REACTIONS DESCRIBED
