# Supplementary material for: Rare, Serious, and Comprehensively Described Suspected Adverse Drug Reactions Reported by Surveyed Healthcare Professionals in Uganda
Source: PLoS One. 2015 Apr 23;10(4):e0123974. doi: 10.1371/journal.pone.0123974 (PMC4408100; doi:10.1371/journal.pone.0123974)
Supplement: S8 Appendix — (PDF) [file pone.0123974.s008.pdf]

Appendix S8: Case Reports of suspected ADRs

Rare suspected ADRs

Case reports of rare ADR descriptions include: 62-year-old female on oral cotrimoxazole within two days got multiple skin patches, and developed sores on mucous membranes with high temperature. She was given steroids and she recovered (reported by a 32-year-old male HCP from the National Referral hospital, Kampala, Central Uganda); 26-year-old HIV-positive male patient on oral cotrimoxazole got severe burnt face and lips and was given oral dexamethasone for five days (reported by 20-year-old female enrolled nurse in a public health centre III, Jinja, Eastern Uganda); a patient got severe palpitations due to overdose of omeprazole (reported by a 31-year-old female registered nurse midwife at the National Referral hospital, Kampala, Central Uganda); a child on intravenous ciprofloxacin got body itch, and swelling around the face and site of the injection (reported by a 30-year-old female doctor from a public health centre III in Wakiso, Central Uganda); a patient on ciprofloxacin got swollen hands (reported by a 26-year-old female registered nurse at the National Referral hospital, Kampala, Central Uganda); 62-year-old female on oral mephaquine (mefloquine) got severe headache with mental confusion and insomnia (reported by a 62-year-old male doctor in a private not-for-profit health centre IV in Masindi, Western Uganda). Many rare ADRs also appear in subsequent sub-sections on quinine, artemisinin-based combinations, and post-exposure prophylaxis, among others.

Quinine-associated suspected ADRs (co-administrations in *italics*)

Two miscarriages by expectant mothers who had received quinine were described by HCPs as follows: a 28-year-old female receiving intravenous quinine miscarried (reported by a 30-year-old male registered midwife, in a private-for-profit drug shop in Eastern Uganda); a patient on oral quinine experienced an abortion (reported by a 50-year-old female enrolled nurse in a private not-for-profit hospital in southern Uganda). No details about trimester of pregnancy were provided. *Two cases of 5-year-old girls, and one of an 8-year-old girl, referred from a clinic after receiving intravenous quinine and septrin developed blisters all over the body and died on admission (as reported by a) a 27-year-old female enrolled comprehensive nurse in a private not-for-profit hospital in Tororo district-Eastern Uganda, b) a 30-year-old female enrolled comprehensive nurse in a private not-for-profit hospital in Tororo district-Eastern Uganda, and c) a 29-year-old female nursing assistant in a private not-for-profit hospital in Tororo district-Eastern Uganda).*

Ten non-fatal suspected ADRs also implicated quinine: hypoglycaemia after intravenous quinine in a 3-year-old child and hypoglycaemia after intramuscular quinine in a 2-year-old child (reported respectively by a 32-year-old female registered nurse in a regional referral hospital (public) in Jinja district, Eastern Uganda and by a 24-year-old male enrolled comprehensive nurse in a private not-for-profit hospital in Tororo district, Eastern Uganda); a 17-year-old receiving intramuscular quinine developed severe post injection paralysis (reported by 60-year-old female enrolled nurse in a private-not-for-profit hospital in Mbarara district, Southern Uganda); a 27-year-old patient on intravenous quinine developed tinnitus and altered consciousness (reported by 35-year-old male doctor in a private-for-profit health centre III in Kampala district, Central Uganda); body itching after oral quinine developed in a 38-year-old female and a 55-year-old male patient (reported respectively by 32-year-old male clinical officer in a private-for-profit health centre III in Kampala, Central Uganda

and by 40-year-old male nursing assistant in a drug shop in Kamuli district, Eastern Uganda). In addition, a 35-year-old female pharmacist in a private community pharmacy in Jinja, Eastern Uganda reported a patient who developed body itching after taking quinine (route not given) and who was treated with cetirizine, an oral antihistamine; a patient with unspecified age and gender vomited and became irritable after intravenous quinine (reported by 26-year-old female registered midwife in a private-not-for-profit health centre IV in Kampala, Central Uganda); *19-year-old patient with malaria and cough who reacted severely to a combination of intravenous quinine and oral septrin* and was managed with parenteral hydrocortisone (reported by a female doctor in a private for-profit health centre IV in Kampala district-Central Uganda). Finally, a 21-year-old female nursing assistant in a private for profit health centre IV in Kamuli, Eastern Uganda incompletely described the case of a 25-year-old who reacted to oral quinine and was told to take a lot of fluids.

Co-administered drugs-associated ADRs

All five ADR descriptions involving simultaneous administration of two drugs from the most frequently occurring medication classes were severe and four of them involved co-administration of an antibacterial and an antimalarial, see **Table 1**. Descriptions of the four antibacterial-antimalarial co-administration cases were given above – *Italics*. One case with antibacterial-antiretroviral co-administration involved a 30-year-old woman on nevirapine and cotrimoxazole who developed a skin rash (reported by a male registered nurse at a public health centre IV).

Artemisinin-based Combination Therapy (ACT)-associated suspected ADRs

Vomiting by a 9-month-old baby on oral artemether-lumefantrine (coartem) was reported by a 27-year-old female doctor in a public district hospital in Tororo, Eastern Uganda; moderate severity swelling of the face in a 16-year-old patient after oral coartem was reported by a 34-year-old male HCP in a private not-for-profit health centre IV in Kampala, Central Uganda; 26-year-old male on oral coartem got rash on both hands, itching and swelling (reported by a 27-year-old female registered nurse in a private for-profit hospital in Lira, Northern Uganda); 52-year-old female on oral coartem developed sores on the whole body (reported by a 24-year-old male doctor in a public health centre IV in Jinja, Eastern Uganda); and severe hypoglycaemia in an adult on artesunate-amodiaquine reported by a male doctor in a public health centre IV in Kampala, Central Uganda; among others.

Post-exposure prophylaxis (PEP)-associated suspected ADRs

A 22-year-old female nurse got a needle-stick injury and was initiated on post-exposure prophylaxis (PEP**)** with zidovudine-lamivudine. She developed Stevens-Johnson syndrome (severe rash) 12 days later and was admitted and treated (reported by a 26-year-old female pharmacist in a private not-for-profit hospital in Gulu, Northern Uganda). A 27-year-old female on post-exposure prophylaxis got blisters with burning sensation all over the body three days later (reported by a female 36-year-old registered nurse in a private not-for-profit hospital in Gulu, Northern Uganda). A patient on PEP with zidovudine-lamivudine-efavirenz presented with euphoria and decreased sleep; efavirenz-induced neuropsychiatric mood (reported by a 28-year-old male doctor at the National Referral Hospital in Kampala, Central Uganda).

Nevirapine-associated Hepatotoxicity

A 45-year-old female developed deep jaundice (hepatotoxicity) after receiving oral nevirapine. She was given efavirenz as alternative drug and she improved (reported by a 32-year-old HCP at a private for-profit health facility in Tororo, Eastern Uganda); oral administration of nevirapine in patient leading to hepatotoxicity (reported by a female doctor in a private for-profit hospital in Wakiso, Central Uganda).

Analgesics-associated suspected ADRs

Severe haemoptysis (coughing up of blood) in a patient after two days on oral diclofenac (reported by a 28-year-old male doctor in a private for-profit hospital in Kampala, Central Uganda); 28-year-old female given intramuscular morphine got generalized skin itching of moderate severity (reported by a 38-year-old female registered nurse-midwife in a private not-for-profit hospital in Kampala, Central Uganda); patient on oral tramadol experienced persistent severe itchy sores all over the body (reported by a 30-year-old male HCP in a private for-profit hospital in Kampala, Central Uganda; among others.

Nifedipine-associated suspected ADR

A male adult developed erectile dysfunction due to nifedipine (reported by a 24-year-old male HCP in a private for-profit health centre III in Kampala, Central Uganda).
